# Role of disordered bipolar complexions on the sulfur embrittlement of nickel general grain boundaries

**DOI:** 10.1038/s41467-018-05070-2

**Published:** 2018-07-17

**Authors:** Tao Hu, Shengfeng Yang, Naixie Zhou, Yuanyao Zhang, Jian Luo

**Affiliations:** Department of NanoEngineering; Program of Materials Science and Engineering, University of California San Diego, La Jolla, CA 92093-0448 USA

## Abstract

Minor impurities can cause catastrophic fracture of normally ductile metals. Here, a classic example is represented by the sulfur embrittlement of nickel, whose atomic-level mechanism has puzzled researchers for nearly a century. In this study, coupled aberration-corrected electron microscopy and semi-grand-canonical-ensemble atomistic simulation reveal, unexpectedly, the universal formation of amorphous-like and bilayer-like facets at the same general grain boundaries. Challenging the traditional view, the orientation of the lower-Miller-index grain surface, instead of the misorientation, dictates the interfacial structure. We also find partial bipolar structural orders in both amorphous-like and bilayer-like complexions (a.k.a. thermodynamically two-dimensional interfacial phases), which cause brittle intergranular fracture. Such bipolar, yet largely disordered, complexions can exist in and affect the properties of various other materials. Beyond the embrittlement mechanism, this study provides deeper insight to better understand abnormal grain growth in sulfur-doped Ni, and generally enriches our fundamental understanding of performance-limiting and more disordered interfaces.

## Abstract (web summary)

Sulfur at nickel grain boundaries can cause catastrophic failure, but the mechanisms behind that embrittlement remain poorly understood. Here, the authors image and model bipolar sulfur–nickel structures at amorphous-like and bilayer-like facets of general grain boundaries that cause embrittlement.

## Introduction

Most solid materials are polycrystals consisting of numerous crystallites bonded by grain boundaries (GBs). Virtually all engineered polycrystalline materials contain impurities, the adsorption (a.k.a. segregation) of which, at the GBs, can significantly alter the performance of the materials. This can sometimes occur drastically via phase-like interfacial transitions^1–8^ that lead to catastrophic failures^9–17^. A classic example is the sulfur (S) embrittlement of nickel (Ni), for which the underlying atomistic mechanism has remained elusive after scrutiny and debate for nearly a century^13–16,18^. In a broader context, GB embrittlement (GBE), which can be defined as impurity-induced intergranular brittleness of normally ductile polycrystals^19^, is one widely observed and technologically important, but poorly understood, phenomenon in materials science.

Materials scientists have long recognized that GBs can be treated as interfacial phases that are thermodynamically two-dimensional^4,7^; they have recently been termed complexions^2,5,6,8,20,21^ to differentiate them from thin layers of Gibbs bulk (thermodynamically three-dimensional) phases that wet the GBs. Recent advancements in aberration-corrected scanning transmission electron microscopy (AC STEM), along with density function theory (DFT) calculations, revealed unprecedented atomic-level details of ordered interfacial structures or complexions^9,11,12,17,18,22–25^. For example, highly ordered bilayers of Bi adsorbates have been identified at GBs in Ni and Cu to be the root cause of liquid metal embrittlement (a special form of GBE)^9,11,17,26^. Ordered interfacial structures have also been observed at ceramic GBs^23–25^. In contrast, more disordered interfaces, despite their widespread existence and technological importance, are still poorly understood, because it is more challenging to characterize and model them. One relatively well-characterized case is represented by the nanometer-thick intergranular films (IGFs) at ceramic GBs^27–30^ and ceramic–metal interfaces^21^, where the AC STEM also revealed partial structural orders in these so-called glassy IGFs. In general, the atomic-level structures of the omnipresent disordered general GBs that often limit the properties of engineering polycrystalline materials remain elusive (albeit general GBs in certain metallic systems can sometimes become highly ordered via reconstruction^11,17,26^). Furthermore, although both metallic^9,11,17,22,26^ and ceramic^23–25,27,28^ interfaces have been well-characterized, the adsorption of S, O, H, P, and other nonmetal elements at metallic GBs is much less understood.

It has long been recognized that the GB adsorption of S can cause catastrophic brittle failures of the otherwise ductile Ni, and other metallic alloys, at low stress levels^13,18^. This is not only a classic example of GBE in physical metallurgy, but also of great technological importance. An early study published in Nature in 1961 attributed GBE to the equilibrium GB adsorption^10^. Rice and Wang^19^ further provided a thermodynamic framework for understanding GBE, but the atomistic mechanisms underpinning GBE have remained elusive.

In a simpler metal-metal system, both electronic^9^ and strain^12^ effects have been proposed to explain the GBE of Cu–Bi, based on the analysis of special symmetric tilt GBs. Recent AC STEM studies further revealed, unexpectedly, highly ordered bilayers of Bi adsorbates at general GBs in both Cu–Bi and Ni–Bi^11,17,26^, providing yet a new perspective.

For Ni–S, DFT calculations^15^ have suggested that the S–S overlap repulsion can lead to GBE. On the other hand, molecular dynamics (MD) simulations^16^, supported by an Auger study^14^, attributed GBE to S induced interfacial amorphization at Ni GBs. Both modeling studies were based on the simplified symmetric Σ5 GB and assumed the GB structures a priori. Furthermore, to our knowledge, no atomic-resolution observation of the actual GB structures has yet been made experimentally.

This study reconciles these two leading theories of S–S overlap repulsion^15^ or interfacial amorphization^14,16^ in an unexpected way via characterizing the equilibrium random-selected general GBs. We examined the GBs in Ni–S via a combination of AC STEM imaging, quantitative energy-dispersive X-ray spectroscopy (EDXS), electron energy loss spectroscopy (EELS), hybrid Monte Carlo and MD (hybrid MC/MD) simulations, and MD tensile testing, as well as several other complementary methods (see the Methods section). Our approach differs from prior studies with advantages in the following two areas. First, our study focused on the asymmetric general GBs randomly selected from polycrystals, since they control GBE in real-world alloys and their behaviors can significantly differ from the special symmetric GBs in the artificial bicrystals studied previously. Second, we determined the equilibrium interfacial structures (instead of a priori selections in virtually all prior modeling studies of GBE), via using a reactive force field (ReaxFF) potential^16^ that integrates quantum-mechanical accuracies into semi-grand-canonical-ensemble simulations. Combining modeling and experiments, we found that the universal formation of two types of bipolar, yet largely disordered, interfacial structures, namely Type A amorphous-like and Type B bilayer-like complexions, at faceted general GBs in S-doped Ni is the root cause for GBE in S-doped Ni.

## Results

### Three types of interfacial structures

In this study, polycrystalline Ni samples saturated with S were isothermally annealed via four routes to achieve thermodynamic equilibria and were quenched (see Methods). Two distinct interfacial structures (Type A amorphous-like and Type B bilayer-like GB facets) that often co-exist in the same general GBs, along with several nominally clean Type C GBs, were identified based on the characterization of the 34 GB facets randomly selected from the Ni–S polycrystals. The detailed experimental observations are summarized in Table 1, as well as Supplementary Fig. 1 and Supplementary Note 1. To briefly summarize, our observations include 18 independent Type A amorphous-like GB facets that all have one lower-index terminal grain surface of the (100) plane with another higher-index matching grain surface; 11 Type B bilayer-like GB facets that have one lower-index grain surface of the (310), (311), (211), or (110) plane with another higher-index matching grain surface; and 5 Type C nominally clean GBs, including four Σ3 symmetric twin boundaries and one low-angle GB.

**Table 1:** Summary of experimental observations and comparisons with simulated results. Grain boundaries were extracted and analysed from seven different samples.

	GB (×Facets)	Annealing *T* (*T*_eutectic_ = 650 °C)	Observed type	Orientation relationship	Thickness *h* (nm)	Measured adsorption *Γ* (S atoms/nm^2^)	Simulation GB model	Simulated type	Simulated adsorption *Γ* (S atoms/nm^2^)	Simulated results shown in
	#1A × 8	675 °C	A	(100)//~(532)_~1° off_	0.92 ± 0.17	34.9	(100)//(926)	A	23.9	Fig. 3
	#1B × 2	675 °C	B	(310)//~(744) _~1° off_	<0.3	12.6	(310)//(457)	B	12.1	Supp. Fig. 42
	#1B × 2						(310)//(3$\bar 1$0)	B	12.2	Supp. Fig. 43
	#2A × 2	675 °C	A	(100)//~(7 11 11)	0.78 ± 0.06		(100)//(926)	A	23.9	Fig. 3
	#3A × 5	675 °C + 575 °C	A	(100)//~(211) _~1° off_	0.89 ± 0.14	15.4				
	#3B × 6	675 °C + 575 °C	B	(311)//~(511) _~3° off_	<0.4	12.6				
	#4A × 3	575 °C	A	(100)//~(320) _~6° off_	0.80 ± 0.13	24.6	(100)//(926)	A	23.9	Fig. 3
	#4B × 2	575 °C	B	(211)//~(403) _~3° off_	<0.4	6.2				
	#5B × 4	500 °C	B	(110)//~(331) _~2° off_	<0.3	7.0	(110)//(345)	B	9.63	Fig. 4
	#6C	675 °C + 575 °C	C	(111)//(111) Twin	~0	~0	(111)//(111) Twin	C	~0	Supp. Fig. 44
	#7C	675 °C + 575 °C	C	(111)//(111) Twin	~0	~0	(111)//(111) Twin	C	~0	Supp. Fig. 44
	#8C	575 °C	C	Low-angle GB	~0	~0				
	#9C	500 °C	C	(111)//(111) Twin	~0	~0	(111)//(111) Twin	C	~0	Supp. Fig. 44
	#10C	500 °C	C	(111)//(111) Twin	~0	~0	(111)//(111) Twin	C	~0	Supp. Fig. 44

In this study, we examined 34 independent GBs or GB facets, including: (A) 18 independent GB facets that have one lower-index terminal grain surface of the (100) plane with another higher-index matching grain surface, which were all found to be Type A (amorphous-like); (B) 11 GB facets (and 14 independent locations) that have one lower-index grain surface of the (310), (311), (211), or (110) plane with another higher-index matching grain surface, which were all found to be Type B (bilayer-like); and (C) 5 GBs are nominally clean (Type C), where four were found to be Σ3 (111)//(111) symmetric twin boundaries and another was determined to be a low-angle GB. The results clearly suggest the formation of complexions correlated with the orientation of the lower-index grain terminating plane, instead of the misorientation. Thus, in the semi-grand-canonical-ensemble atomistic simulations, we selected one grain of the GB model to match exactly the lower-Miller-index plane observed in the experiment, while the other grain was chosen to be similar (but not identical) to that observed in the experiment to allow the application of a periodic condition. As an example of the notations used in Column 1, “#1A × 8” means that eight independent (disconnected), parallel facets on GB #1 were examined and all eight were Type A. Noting that GB #5B is flat and long; we examined four different locations that were far apart, all of which were Type B. See Supplementary Note 20 and Supplementary Table 3 for a critical comparison of simulation and experiments.

### GB faceting

**Fig. 1:**
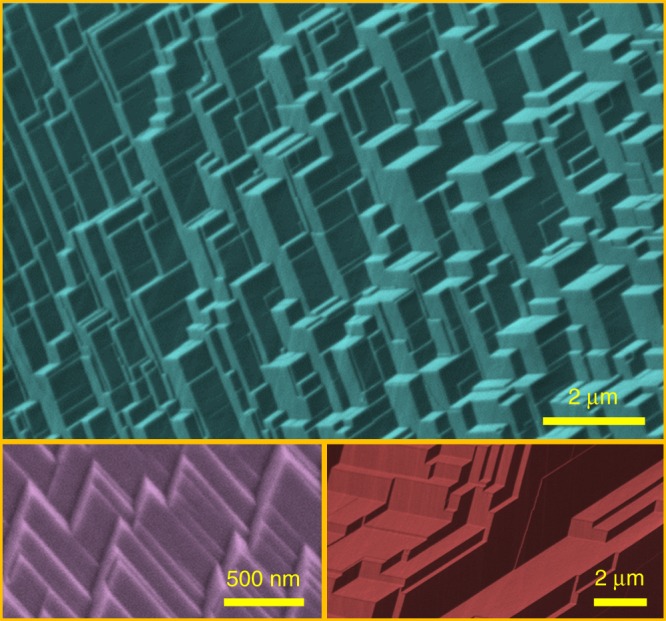
Representative faceted grain boundaries (GBs) in S-doped Ni. Selected scanning electron micrographs of fractured grain surfaces, showing GB faceting. Additional images and specific conditions for preparing specimens are shown in Supplementary Figs. 45–48

The majority of GBs in S-doped Ni are highly faceted (Fig. 1); please also see Supplementary Table 1 for the statistics of faceted GBs (e.g., ~54% faceted GBs at 500 °C, which increased to ~84% at 675 °C) and Supplementary Figs. 45–48 for additional images.

### Type A and B facets coexisting at the same general GB

**Fig. 2:**
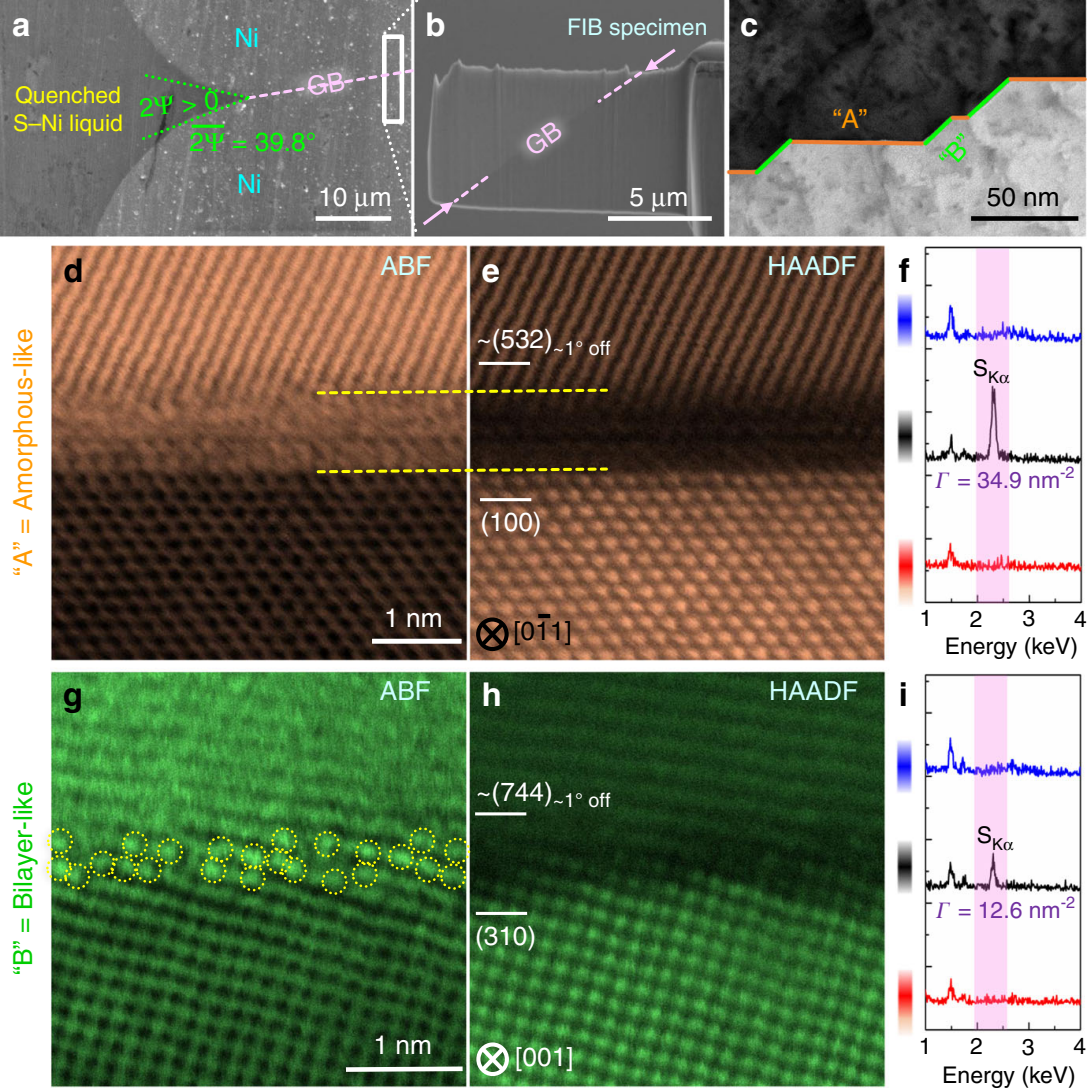
A representative general (randomly selected asymmetric high angle) grain boundary (GB) in S-doped Ni with coexisting Type A and B facets. **a** Scanning electron micrograph of an Ni–S liquid interacting a GB with a non-zero dihedral angle in a specimen quenched from an equilibrium at 675 °C. **b** A TEM specimen containing a GB extracted by focused ion beam. **c** A low-magnification STEM image showing the alternating Type A and B facets. A pair of synchronized (**d**) annular bright field (ABF) and (**e**) high-angle annular dark field (HAADF) images, along with (**f**) the corresponding EDX spectra, of a representative Type A amorphous-like facet (out of eight independent Type A facets examined along this same GB; see others in Supplementary Figs. 2 and 17). The excess adsorption of S measured by box scanning to be: *Г* = 34.9 nm^−2^. A pair of synchronized (**g**) ABF and (**h**) HAADF images, along with (**i**) the corresponding EDX spectra, of a Type B facet. This facet is also S-enriched, but it has a smaller *Г* of 12.6 nm^−2^

Figure 2 illustrates a representative general (i.e., randomly selected, high-angle, asymmetric) GB, wherein two sets of alternating facets exhibited distinct characteristics. On the one hand, the Type A (100)//~(532) facets exhibit amorphous-like, nanometer-thick IGFs (Fig. 2d, e). On the other hand, the Type B (310)//~(744) facets of the same GB are more crystalline (Fig. 2g, h) than the Type A amorphous-like IGFs; subsequent simulations and analyses show that they are disordered bilayer-like (Type B) complexions (that are much more disordered than the highly ordered bilayers observed in Ni–Bi and Cu–Bi^11,17,26^). The EDXS (Fig. 2f, i) and EELS (Supplementary Fig. 13) showed that both facets are S-enriched. Using an STEM box scanning method (Supplementary Note 5), the GB excess of S was measured to be ~34.9 S atoms/nm^2^ for this Type A facet/complexion, being significantly higher than the measured value of ~12.6 S atoms/nm^2^ for the Type B facet/complexion with an identical GB misorientation.

Both Type A and B facets (complexions) have been observed in different general GBs in multiple specimens equilibrated at different temperatures (Table 1; Supplementary Figs. 1, 28–30, and 32–38). Through-focus images were recorded to confirm the edge-on conditions and verify the uniformity along the e-beam direction (Supplementary Figs. 14–15 and Supplementary Note 7). The observed alternating formation of the Type A and B facets (complexions) is consistent throughout an entire GB (Supplementary Figs. 29 and 30), implying a thermodynamic equilibrium. More specifically, we examined eight independent, parallel, (100)//~(532) facets along the GB shown in Fig. 2c. They are all Type A with similar structural characteristics (Supplementary Figs. 17 and 29).

### Factors determining the interfacial structure

In contrast to a traditional view, the orientation of the lower-Miller-index terminal grain surface of the GB facet, instead of the misorientation, dictates the interfacial width and the level of the disorder.

Here, we use the symbol (100)//~(532) _~1° off_ to denote a facet, where (100) is the lower-index terminal grain plane that is exact and dictates the facet orientation. The other matching grain surface has a higher or irrational index that is ~1° off the (532) orientation; the subscript is often omitted for brevity.

The Type A amorphous-like IGFs formed at (100)//~(532), (100)//~(7 11 11), (100)//~(211), and (100)//~(320) facets; all have a (100) surface as the dictating lower-Miller-index plane (Figs. 2 and 3; Table 1; Supplementary Figs. 1, 17, 28, 29, 32–35, and 37). The Type B complexions formed at (310)//~(744), (311)//~(511), (211)//~(403), and (110)//~(331) facets (Figs. 2 and 4; Table 1; Supplementary Figs. 1, 28, 30, 32–36, and 38).

**Fig. 3:**
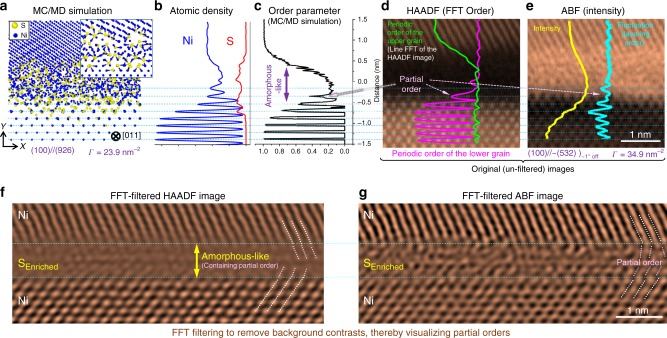
Atomic structure of the Type A (amorphous-like) complexion or IGFs of an equilibrium thickness. **a** Hybrid MC/MD simulated (100)//(926) GB, where the inset is an enlarged and thinner section. The corresponding (**b**) Ni and S density profiles and (**c**) order parameter profile. A pair of experimental (**d**) HAADF image (with overlapping periodic order profiles obtained from the line-by-line FFT) and (**e**) ABF image of the (100)//~(532)_~1° off_ GB facet (with overlapping intensity profile and its fluctuation, illustrating the layering order). FFT-filtered (**f**) HAADF and (**g**) ABF images to remove the background contrasts to reveal a bipolar distribution of partial structural orders within this amorphous-like intergranular film (IGF)

**Fig. 4:**
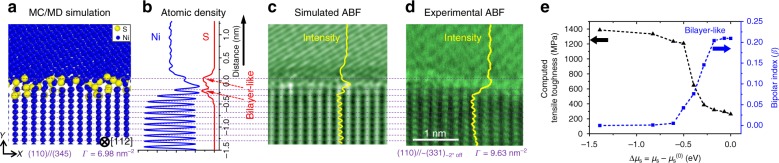
Atomic structure of the Type B (bilayer-like) complexion. **a** Hybrid MC/MD simulated (110)//(345) GB. The corresponding (**b**) Ni and S density profiles. **c** Simulated ABF image using the atomic structure obtained by the hybrid MC/MD simulation. **d** Experimental ABF image of a (110)//~(331)_~2°off_ GB facet. See two additional examples of Type B complexions in Supplementary Figs. 33 and 34. **e** Computed tensile toughness and bipolar index *β* curves with increasing S chemical potential, suggesting a strong correlation between the formation bipolar interfacial structures and embrittlement. See additional analyses in Supplementary Fig. 9 and further discussion about the bipolar index *β* in Supplementary Notes 2 and 16, as well as discussion of embrittlement and computed tensile toughness in Supplementary Notes 17 and 18

The stabilization of the nanometer-thick IGFs on the Type A (100) facets can be explained by the low liquid-Ni (100) interfacial energy (stabilizing a liquid-like IGF), which was confirmed experimentally via observing the Wulff shape of the Ni particles in an S-enriched liquid (Supplementary Fig. 4).

The fact that the Type A and B facets (complexions) often co-exist at one GB (with the same two abutting grains, thereby having the identical misorientation; see, e.g., Fig. 1 and Supplementary Fig. 1) directly proved that the misorientation is not the controlling factor here.

### Type A amorphous-like IGFs with an equilibrium thickness

A narrow distribution of measured interfacial widths of 0.92 ± 0.17 nm (Supplementary Figs. 3B, 17; Supplementary Note 12) suggests the existence of a thermodynamically determined or equilibrium thickness^29,30^. This is akin to those IGFs widely observed at ceramic GBs^29,30^ and it represents, to our knowledge, the first such observation in a metal–nonmetal system. It is worth noting that amorphous-like IGFs or glassy-like GBs have also been discovered in several metallic systems^31–33^, where they can significantly affect the materials processing^31^, microstructural evolution^32^, and mechanical properties^33^.

These amorphous-like (or liquid-like) IGFs formed both above and below the bulk eutectic temperature of the Ni–S system (Table 1). The stabilization of liquid-like IGFs at Type A GB facets below the bulk eutectic temperature (where the bulk liquid phase is no longer stable) is analogous to the well-known phenomenon of surface melting or premelting, originally proposed by Faraday^34^.

Moreover, the liquid-like IGFs persist above the bulk eutectic temperature with a nanoscale equilibrium thickness (against unconstrainted thickening), in equilibrium with a bulk liquid phase at grooves with non-zero dihedral angles (mean ≈ 39.8° at 675 °C; see Supplementary Figs. 3C, D, as well as Fig. 2a); this result unequivocally demonstrates that the interfacial energy of an IGF (*γ*_IGF_, which is the GB energy *γ*_gb_ at a thermodynamic equilibrium) is less than that of two crystal-liquid interfaces (*γ*_gb_ ≡ *γ*_IGF_ < 2*γ*_cl_). Thus, these Type A amorphous-like IGFs are not wetting films, but a true thermodynamically two-dimensional interfacial phase or complexion^21^.

### Partial structural order in amorphous-like IGFs

The line-by-line FFT analyses of the STEM images revealed partial structural orders in the Type A amorphous-like IGFs (the overlapping profiles on Fig. 3d; elaboration in Supplementary Note 13 with additional examples in Supplementary Figs. 18–20), which are consistent with the atomic density (Fig. 3b) and order parameter (Fig. 3c) profiles predicted by the hybrid MC/MD simulation. The FFT filtering (Supplementary Note 8) of the STEM images (Fig. 3f, g) further illustrates a bipolar distribution of partial orders, with an abrupt change in crystalline orientation in the middle of the IGF, concurring with the prediction of a diffuse-interface model^5,6^.

### Semi-grand-canonical-ensemble atomistic simulation

Hybrid MC/MD simulations in semi-grand canonical ensembles with a DFT-derived ReaxFF potential were used to obtain the equilibrium interfacial structures using several GB models selected to resemble the GB facets characterized in the experiments (see Supplementary Note 9 for the calibration of the potential).

While there is in general no lattice match between the two abutting grains in the randomly selected GBs in experiments, periodic boundary conditions must be used for simulations. Fortunately, our experiments suggested that the lower Miller index of one of the grains determined the interfacial width and the level of disorder (regardless of the misorientation), enabling an approach to select only the left grain of the GB model to match exactly the low-Miller-index plane observed in the experiment for a valid comparison. This approach has been further validated by subsequent modeling and comparing GBs of different misorientations but identical low-index dictating grain surface; see Supplementary Notes 10 and 19 for further detailed discussion and justification.

We further computed the STEM images based on the simulated GB structures to compare them with the experimental images (Fig. 4c vs. Fig. 4d; Supplementary Figs. 5  and 42–44). The simulated GB excesses of S (the Γ values labeled in Figs. 2–4 and Supplementary Fig. 5; also summarized for more cases in Table 1 and Supplementary Table 3 and further discussed in Supplementary Note 20) and the disorder parameter profiles (Fig. 3c and Supplementary Fig. 41) agree with the experiments for Type A and B complexions.

### Type B disordered bilayer-like complexions

Simulated (110)//(345) (Fig. 4b), (310)//(457) (Supplementary Fig. 42B), and (031)//(03$\bar 1$) (Supplementary Fig. 43B) GBs show that S adsorbates are largely distributed within two atomic layers in these Type B complexions.

**Fig. 5:**
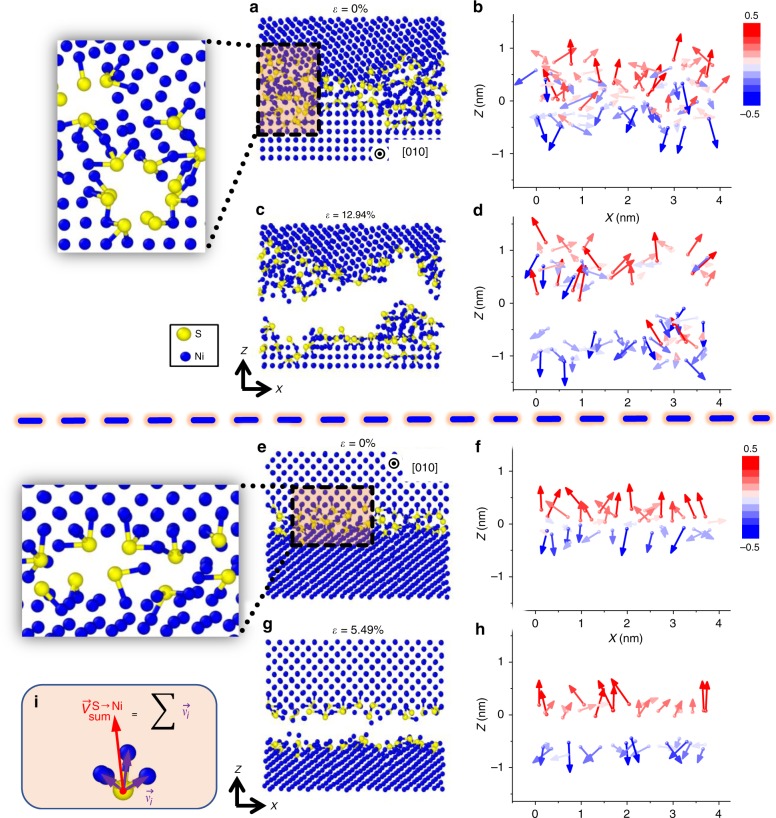
Atomistic simulations revealing disordered bipolar interfacial structures and the embrittlement mechanism. **a**–**d** Type A amorphous-like (100)//(926) GB. **e**–**h** Type B bilayer-like (110)//(345) GB. Images on the left are close-ups of the GB atomic structures. Images in the middle column illustrate the atomic structures before and during the molecular dynamics tensile testing (with the applied strains labeled). **i** The definition of the $\vec V_{{\mathrm{sum}}}^{{\mathrm{S}} \to {\mathrm{Ni}}}$ vector. Images in the right column show the bipolar distributions of $\vec V_{{\mathrm{sum}}}^{{\mathrm{S}} \to {\mathrm{Ni}}}$ vectors that lead to intergranular fracturing during tensile testing; furthermore, a bipolar index (*β*) is defined (in Methods and Supplementary Note 16) and quantified from the simulated results; this *β* correlates with the computed tensile toughness (Fig. 4e; Supplementary Fig. 9C and 9D). The arrows illustrate the projections of the $\vec V_{{\mathrm{sum}}}^{{\mathrm{S}} \to {\mathrm{Ni}}}$ vectors pointed from each S atom in the *x*–*z* plane and the color contour shows the *z* component of the vectors

To better illustrate their bilayer-like nature and further reveal any hidden bipolar interfacial structures, the polar S–Ni structures in the simulated GBs are identified. For each S atom, we define a vector, $\vec V_{{\mathrm{sum}}}^{{\mathrm{S}} \to {\mathrm{Ni}}}$, as the sum of all vectors pointing from this S atom to all bonded Ni atoms (Fig. 5i; Supplementary Fig. 6 and Supplementary Note 16). Figure 5f and Supplementary Fig. 8D display the bipolar distributions of the $\vec V_{{\mathrm{sum}}}^{{\mathrm{S}} \to {\mathrm{Ni}}}$ vectors (mostly) within the two layers, largely pointing in opposite directions.

These bilayer-like Type B complexions appear to be more crystalline than the Type A amorphous-like IGFs (Figs. 2–4), but it is important to note that they are much more disordered than the highly ordered bilayers of Bi adsorbates observed at the general GBs in Ni and Cu^11,17,26^. Thus, we consider the Type B complexions as bilayer-like but disordered (i.e., with roughly two layers of polar S–Ni structures that are largely, but not perfectly, aligned in opposite directions, as shown in Fig. 5f and Supplementary Fig. 8D), representing a type of complexion that has not been recognized, characterized, and understood previously.

### Bipolar interfacial structures in Type A amorphous-like IGFs

The bipolar distributions of the $\vec V_{{\mathrm{sum}}}^{{\mathrm{S}} \to {\mathrm{Ni}}}$ vectors were also found in a Type A amorphous-like IGFs (Fig. 5b and Supplementary Fig. 8B), but they are more disordered and spanned over a wider nanoscale thickness, supporting the experimental observations illustrated in Fig. 3f, g (as well as Supplementary Figs. 32–37). However, the bipolar index *β* deceases with increasing interfacial disorder/width (e.g., during the transition from a bilayer-like to an amorphous-like interfacial structure at the (100)//(926) GB with increasing bulk chemical potential of S, as shown in Supplementary Fig. 9D). See Supplementary Fig. 41 for a further comparison of both the simulated atomic density and order profiles of Type A vs. Type B GBs.

### MD tensile simulations

To better understand the GBE mechanisms, we performed additional MD simulations of tensile testing on both the S-doped and pure Ni GBs at 300K (Fig. 5; Supplementary Figs. 7, 8, 25 and 26; Supplementary Note 17). The results reveal that the GB S adsorption reduces the strength and ductility in all cases. The polar S–Ni structures remained mostly intact after the fracture, presumably due to the strong S–Ni bonds. Thus, the intergranular decohesion mainly occurred as a result of the separation between the bipolar S–Ni structures that were aligned head-to-head (S-to-S). This is evident in the distributions of the $\vec V_{{\mathrm{sum}}}^{{\mathrm{S}} \to {\mathrm{Ni}}}$ vectors in the snapshots in Fig. 5 (and Supplementary Fig. 8).

### Correlation between a bipolar index *β* and embrittlement

Figure 4e and Supplementary Fig. 9 further illustrates the variations of the computed tensile toughness (defined in computation to represent the relative extent of GBE, which is not the fracture toughness; see Supplementary Note 18 for the definition and further discussion) and several computed GB parameters with the increasing S chemical potential for both Type A and B GBs. In each case, an abrupt decrease in the computed toughness occurs at a critical chemical potential, concurrently with the increase in a bipolar index *β* (defined in Methods), as shown Fig. 4e and Supplementary Fig. 9C, D. Therefore, it is inferred that the formation of the bipolar interfacial structures, induced by the S adsorption, lead to GBE.

### Type C clean GBs

Among 34 independent GBs or GB facets randomly selected from the Ni–S polycrystals and examined in this study (Table 1), five of them are nominally clean (Type C) with no measurable S adsorption (below the detectable limits of EDXS): four were found to be Σ3 (111)//(111) symmetric twin boundaries and another was determined to be a low-angle GB (Supplementary Figs. 31, 39 and 40). Thus, all the Type C GBs observed are low-energy GBs with little driving force for S adsorption. Low adsorption and no structural transformation at low-energy interfaces are well expected in interfacial thermodynamics. See elaboration in the last paragraph in Supplementary Note 20.

## Discussion

We report the co-existence of two bipolar and largely disordered interfacial phases (complexions) at faceted general GBs in Ni–S, as well as related unforeseen interfacial phenomena that enrich the understandings of interfacial complexions. Both the S–S repulsion^15^ and S-induced interfacial amorphization^14,16^, suggested previously by the modeling of a simplified symmetric Σ5 GB, play roles resulting in GBE. However, these effects mingle in an unexpected way at asymmetric, large-angle, general GBs via interplays among interfacial adsorption, ordering vs. disordering (premelting), faceting, and decohesion.

In conclusion, the ubiquitous formation of two types of disordered bipolar complexions at faceted general GBs in S-doped Ni causes brittle intergranular fractures between the polar S–Ni structures that are aligned in opposite directions. Both Type A amorphous-like IGFs and Type B bilayer-like complexions, which often form, in an equilibrium one another, at two alternating facets of the same general GBs, are considered as disordered bipolar complexions and result in severe GBE.

Based on our findings, potential strategies to mitigate GBE include designing heat treatments or co-doping strategies to destabilize bipolar interfacial complexions that are the root cause of GBE; see general discussion about utilizing interfacial phase-like transformations to tailor GB properties and optimize materials fabrication processing, guided by GB complexion (phase) diagrams, in refs. ^2,35–42^. We may also introduce a co-segregating alloying element that may frustrate the bipolar interfacial structures at general GBs to remediate embrittlement.

Similar disordered bipolar interfacial complexions and GBE mechanisms may also exist in other materials systems, particularly metal–nonmetal systems including the S and O induced embrittlement of various metals and metallic alloys of great practical importance, where the atomic-level mechanistic understanding is particularly lacking. Similar interfacial complexions may play roles in GB-controlled oxidation and corrosion, as well as hydrogen embrittlement.

An important merit of this study is its focus on disordered and asymmetric general GBs (of mixed tilt and twist character), which are often the performance-limiting weak links chemically and mechanically, but are poorly understood. Current literature often focuses on symmetric tilt or twist GBs in artificial bicrcystals, for the experimental and computational convenience. Thus, this study supplements and enriches the literature by investigating randomly selected general GBs in polycrystals.

Our simulations showed that the asymmetric nature (mixed tilt and twist character) of the GB is more important than the Σ value. For example, an asymmetric (310)//(457) Σ3 GB exhibits a Type B bilayer-like complexion (Supplementary Table 3 and Supplementary Fig. 42, as well as discussion in Supplementary Note 20) despite its low Σ value, which differs drastically from the symmetric (111)//(111) Σ3 twin boundary (that is a Type C clean GB with little S adsorption). Here, the orientation of grain surfaces, instead of the misorientation between the two abutting grains (being identical for both asymmetric vs. symmetric Σ3 GBs), dictates the interfacial structure.

In general, an important finding of this study is that the orientation of the lower-Miller-index grain surface, instead of the misorientation as commonly believed, determines faceting, interfacial width, and the level of interfacial disorder (Table 1), in contrast to the traditional view.

Beyond the GBE, the above-discussed finding of anisotropic complexion formation determined by lower-Miller-index terminal grain surface plane orientation also provided a new insight about the mysterious origin of abnormal grain growth in S-doped Ni (including electrodeposited nanocrystalline Ni with S contamination). As it is known that the abnormal grains in S-doped Ni are often cubic with (001) terminal surface planes^43,^ this current study showed that such GB facets with the (001) plane as the lower-Miller-index dictating grain surface plane exhibit Type A amorphous-like IGFs that are more disordered with higher levels of S absorption than other (Type B or C) GB facets (Fig. 1; Table 1; Supplementary Note 20). Thus, we propose that abnormal grain growth in S-doped Ni is likely a result of enhanced GB mobility of the more disordered Type A amorphous-like GBs, akin to that observed and proposed by Dillon et al.’s for doped Al_2_O_3_ and other systems^2,20,44.^ Here, the abnormal grain growth can be resulted from the highly anisotropic nature of complexion formation in S-doped Ni (and may depend less on the occurrence of a complexion transition). See Supplementary Note 21 for detailed discussion.

In general, the current work advances our fundamental knowledge of more disordered interfaces that are both scientifically interesting and technologically important, and that are currently much less understood than the well-characterized ordered interfaces^9,11,12,17,18,22–25^.

## Methods

### Sample preparation

To prepare the S-doped Ni specimens, Ni plates (99.9945% purity) were ground and polished to mirror surfaces. The polished Ni plates were cleaned ultrasonically in acetone and ethanol sequentially. Sandwich structures, which consisted of two Ni plates with S powder (99.98% purity) between them, were constructed and subsequently annealed in a tube furnace. First, the tube furnace was evacuated by a vacuum pump and subsequently purged with a mixed gas of Ar and 5 vol% H_2_. A constant flow of the gaseous mixture was maintained throughout the heat treatment.

The S-saturated Ni specimens were equilibrated using four different annealing routes, i.e., (I) isothermal annealing at 675 °C for 5 h (675 °C × 5 h); (II) isothermal annealing at 675 °C for 5 h, followed by isothermal annealing at 575 °C for 10 h (675 °C × 5 h + 575 °C × 10 h); (III) isothermal annealing at 575 °C for 10 h (575 °C × 10 h); and (IV) isothermal annealing at 500 °C for 168 h (500 °C × 168 h). Note that Sample I was equilibrated with a S-enriched liquid above the eutectic temperature (*T*_eutectic_ = ~650 °C^45^), and Samples II–IV were equilibrated below the eutectic temperature. The experiments were designed so that Sample II and Sample III approached the same solid-state equilibrium temperature of 575 °C from higher (*T*  > *T*_eutectic_) and lower temperatures.

After annealing was completed, all of the samples were water quenched (within ~3 s) to preserve the high-temperature structures. All specimens should be S-saturated at the equilibrium conditions; the bulk compositions of the Ni-based FCC phase should be on the solidus or solvus line at the corresponding (final) equilibrium temperatures. The S contents in the Ni-based FCC phase in these specimens should be within the range of 10–100 ppm, which is well below the detection limits of EDXS and EELS. Please refer to Supplementary Note 11 for further discussion.

### TEM and STEM characterization

Transmission electron microscopy (TEM) specimens were prepared using a focused ion beam (FIB) system (Scios, FEI). A TEM lamellar (12 μm × 4 μm × 1.5 μm) containing a GB was extracted by a lift-out technique and thinned down to ~300 nm by the FIB at 30 kV. Then, it was thinned down to 20–50 nm at 5 kV with a low-beam current. Final cleaning was conducted at 2 kV, using a current of 27 pA, for 3 min for each side of the TEM foil to reduce the surface damage caused by Ga ion beam with high energy.

The characterizations of the microstructures, GB faceting, and atomic-level interfacial structures and compositions were conducted by using high-resolution TEM (HRTEM) and aberration-corrected scanning TEM (AC STEM). The HRTEM images were taken on a Titan 80–300 (FEI) TEM operated at 300 kV. STEM high-angle annular dark field (HAADF) and annular bright field (ABF) images were taken by using a 200 kV STEM (ARM-200F, JEOL) equipped with a probe Cs corrector (CEOS Gmbh) with a sub-ångström resolution. HAADF images were taken via adopting a probe convergence angle of ~22 mrad and using a detector with inner semi-angles of >60 mrad. The ABF images were taken with a detector with semi-angles of 12–23 mrad.

EDXS was used to quantitatively measure the GB compositions. The EELS was recorded using a Gatan Enfinium spectrometer (equipped on the ARM-200F STEM) with an energy resolution (full-width at half maximum) of ~0.5 eV.

### Measurements of the GB excess of S

We used an EDXS-based box scanning method (in STEM, JEOL ARM200F) to quantify the GB excess adsorption of S per unit area (Γ). This was done by measuring the solute concentration in a well-defined volume containing GB, from which the excess concentration per unit area relative to the grain interior (as reference) was determined. The details of this box scanning method can be found elsewhere^46^, and it is explained with an example in Supplementary Note 5 and Supplementary Fig. 12. The measured GB excesses of S per unit area for different GB facets equilibrated at different conditions were summarized in Table 1.

### Indexing of GB terminal planes

We used a lattice imaging method to determine the crystal orientation of the two terminal planes of a GB. Once the grain was tilted into the low-index zone axis, the terminal planes could be indexed unambiguously. The detailed information and an example are presented in Supplementary Note 3 and Supplementary Fig. 10.

### Edge-on conditions of GBs in STEM

An “edge-on” condition for GBs is required to clearly image the GB phases in TEM/STEM^2^. When a GB is set to an edge-on condition, it is parallel to the electron beam in the microscope. To ensure that the GB was set to an edge-on condition, we used a through-focus series of ABF micrographs to examine through the thickness of the TEM specimen.

Through-focus series of ABF images were used to verify that the GBs were uniform along the direction of the TEM specimen’s thickness (i.e., edge-on conditions plus no step and other curvature along the direction of the specimen’s thickness). Examples are shown in Supplementary Figs. 14 and 15, where there was no significant change in thickness and morphology for both Type A and B GBs when the defocus varied. See elaboration and further discussion in Supplementary Note 7.

### Analysis of the partial crystalline order

We selected a rectangular frame in which the width was equal to the lattice spacing. Then, the frame was moved upwards pixel by pixel, where the Fast Fourier Transformation (FFT) was performed, resulting in the “line-by-line FFT” analysis of the (partial) crystalline order. See elaboration and further discussion in Supplementary Note 13. Examples are shown in Supplementary Figs. 18–20.

### STEM imaging simulation

STEM images were simulated by using the QSTEM program^47^, which was based on a multi-slice method^48^. The interfacial structures of various GBs with S segregation obtained from atomistic simulations were used for imaging simulation. The thickness of the simulated sample was 20 nm. The scattering semi-angle for ABF imaging ranged from 15 to 22 mrad, while that for HAADF imaging was >60 mrad. The convergence angle was 22 mrad. The spherical aberration coefficient was 0 μm.

### Semi-grand-canonical-ensemble simulations

Hybrid Monte Carlo (MC) and MD simulations of semi-grand canonical ensembles were conducted using a first-Principles derived ReaxFF potential to investigate the equilibrium interfacial structures. The atomic structural relaxations in the S-doped Ni GBs were modeled by MD steps, while the MC scheme sampled the semi-grand canonical ensemble to enable the determination of the equilibrium concentration and distribution of S for a given chemical potential difference, i.e., Δ*µ* ≡ *µ*_S_ − *µ*_Ni_. The introduction of MC steps also can overcome the problem of the slow diffusion of pure MD simulations. In the semi-grand-canonical-ensemble hybrid MC/MD simulation, the total number of atoms and Δ*µ* were fixed, while the number of S atoms varied. The open-source software, LAMMPS^49^, was used.

A first-principles derived ReaxFF potential^16^ was used in this work to describe interatomic interactions between Ni and Ni, S and S, and Ni and S. See Supplementary Note 9 for testing and calibration of this ReaxFF that was derived from quantum-mechanical density functional theory calculations. Mechanical equilibrium was achieved by using an isothermal–isobaric (NPT) ensemble with zero hydrostatic pressure. During the simulation, five MC attempts were performed after each MD step. In all simulations, the time step was set to 0.1 fs.

GB models with different orientation relationships, as shown below, were created to mimic the observed GBs in TEM. After relaxing the initial GB structures via MD simulation, the hybrid MC/MD method was used to simulate the GBs at the conditions mimicking the experiments (at constant temperature and chemical potential or in a grand canonical ensemble). The GB excess of S per unit area was computed from the equilibrated atomic structures.

### Selection of GB models for simulations

Several GB models with different orientation relationships were used to mimic the GBs observed in experiments. Approximately 10,000 atoms are contained in each GB model for conducting hybrid MC/MD simulation in a semi-grand canonical ensemble using the first-principles derived ReaxFF potential^16^, which is computationally expensive. Because of the use of periodic boundary conditions in the simulation, it is infeasible to exactly match the orientations of both grains observed experimentally. The experimental observations indicate that all GBs have one grain surface with a low Miller index, which dictates the structure and chemistry of the GBs. Therefore, we always selected the left grain of the GB model to match exactly the lower-Miller-index plane observed in the experiment, while the right grain was chosen to be similar (but not identical) to that observed in the experiment to allow the application of a periodic condition. Specifically, a ∑11 (100)//(926) GB facet was selected to represent the Type A GB facets, while ∑15 (110)//(345), ∑11 (310)//(3$\bar 1$0), and ∑3 (310)//(457) GB facets were selected to represent Type B GB facets (Supplementary Table 3). Note that sometimes the modeled GBs are called GB facets because they correspond to the Type A and Type B facets observed in experiments. This is done even though the modeling typically is conducted for stand-alone GBs in bicrystals with periodic boundary conditions (instead of faceted GBs). Our simulation results (discussed in detail in Supplementary Note 20 and summarized in Supplementary Table 3) further supported the hypothesis that the GB structure and chemistry are dictated by the orientation of the lower-Miller-index terminating grain surface plane, instead of the commonly believed misorientation, thereby validating our selection of model GBs. See Supplementary Note 10 for a further justification of selecting these model GBs. See Supplementary Note 20 and Supplementary Table 3 for a critical comparison of the simulated results and experimental observations.

### Calculation of atomic density profiles

To compare the simulated interfacial structures with the experimental results in a more quantitative way, we used a coarse-grained method to compute one-dimensional (1-D) atomic density profiles from simulations. A 1-D Gaussian distribution function^50^ was assigned to each atom. Then, the overall density distribution function can be computed by summing these individual 1-D Gaussian functions to obtain a 1-D atomic density profile:

1 $$\rho \left( x \right) = \frac{1}{{L_YL_Z}}\frac{1}{{\sqrt {2\pi } \sigma }}\mathop {\sum}\limits_{i = 1}^N {\exp \left[ { - \frac{{\left( {x - x_i} \right)^2}}{{2\sigma ^2}}} \right]}$$

where *σ* is the width of the Gaussian distribution (set to 0.3 Å to obtain smooth profiles), *x*_*i*_ is the coordinate of atom *i*, *N* is the number of atoms, and *L*_*Y*_/*L*_*Z*_ is the ratio of the lengths of the simulation box in the *y*/*z* directions.

At equilibrium conditions, it was observed that the Type B GB (Fig. 4b in the main article) had two major peaks in the profile of S density, while the Type A GB (Fig. 3b in the main article) had a wider and more diffuse distribution of S in the atomic density profile.

These atomic density profiles calculated from simulations subsequently were compared with the intensity profiles obtained from experiments (in STEM HAADF and ABF images) for both types of GBs in Fig. 3 and Fig. 4 in the main article, respectively, and in Supplementary Figs. 41–43.

### Calculating structural order parameter and profiles

In order to characterize the structural order (or disorder) of GBs, a bond-orientational order parameter^51^ was calculated for each atom. This order parameter is equal to one for an atom situated in a perfect lattice, and it is equal to zero for an atom in the liquid phase. The 1-D distribution of order parameter was obtained by averaging the order parameter of atoms in the planes that were parallel to the GB plane. It was observed that the order parameter profile of the Type A GB had a broader disordered region than the Type B GB. Figure 3 in the main article compares the order parameter profile from the simulation with the experiments. Supplementary Fig. 41 compares the order parameter profiles of Type A vs. Type B GBs.

### Calculating GB excess in S and GB excess disorder

In simulations, various amounts of S adsorption at GBs were obtained by changing the chemical potential difference between Ni and S (Δ*µ* ≡ *µ*_S_ − *µ*_Ni_). The GB excess in S per unit area (i.e., the amount of S adsorption, *Γ*) was calculated for each case after equilibrium was achieved. In addition, the GB excess in the disorder parameter^51^ was also calculated following ref. ^35^.

### Defining the ${\vec{\bf{V}}}_{{\bf{sum}}}^{{\bf{S}} \to {\bf{Ni}}}$ vector for a polar S–Ni cluster

For each S atom, we defined a $\vec V_{{\mathrm{sum}}}^{{\mathrm{S}} \to {\mathrm{Ni}}}$ vector (to represent the associated polar S-Ni cluster) as the sum of all vectors pointing from this S atom to all bonded Ni atoms. See Supplementary Fig. 6 and Supplementary Note 16 for further elaboration.

### Defining the GB bipolar order index *β*

Subsequently, we defined an interfacial bipolar order index (*β*) as the sum of all dot products of $\hat z \cdot \vec V_{{\mathrm{sum}}}^{{\mathrm{S}} \to {\mathrm{Ni}}}$ associated with each of the S atoms at/near the GB per unit area, where $\hat z$ is a unit vector along the +*z* or −*z* direction if the S atom is located above or below the *z* = 0 GB dividing plane, which was selected so that there were equal numbers of S atoms above and below this *z* = 0 plane. Thus:

2 $$\beta = \frac{{{\sum} {\left( {\vec z \cdot V_{{\mathrm{sum}}}^{{\mathrm{S}} \to {\mathrm{Ni}}}} \right)} }}{A}$$

where *A* is the GB area. See elaboration and further discussion in Supplementary Note 16.

### MD tensile testing

Tensile tests of GBs were conducted by performing MD simulation on the equilibrium GBs. The GB structures obtained from hybrid MC/MD simulations were quenched to and equilibrated at 300 K. The tensile displacement was applied by deforming the simulation box in the direction perpendicular to GB plane. Periodic boundary conditions were applied in the two directions along the GB plane. The MD simulations of tensile tests were conducted at 300 K using the isothermal–isobaric (NPT) ensemble with zero pressure. The strain rate that was applied was about 1.3 × 10^9^ s^−1^. To study the effect of S on the GBs, we also performed tensile tests on the pure Ni GBs that were obtained under the same conditions. The results of the MD tensile tests are presented in Fig. 5, as well as Supplementary Figs. 7, 8, 25, 26. The computed tensile toughness is calculated from integrating the stress–strain curve obtained in MD simulations, which correlates well with the bipolar index *β* to reveal the important mechanistic role of bipolar interfacial structures in GBE. See additional MD tensile simulation results, the definition of this computed tensile toughness, and further discussion in Supplementary Notes 17, 18.

### Data availability

The data that support the findings of this study are available in Supplementary Information accompanies this paper, which includes Supplementary Notes 1–25, Supplementary Tables 1–3, and Supplementary Figures 1–48 and from the authors upon request.

## Electronic supplementary material

Supplementary Information
