# Supplementary material for: Role of disordered bipolar complexions on the sulfur embrittlement of nickel general grain boundaries
Source: Nat Commun. 2018 Jul 17;9:2764. doi: 10.1038/s41467-018-05070-2 (PMC6050256; doi:10.1038/s41467-018-05070-2)
Supplement: Supplementary file 1 — Supplementary Information [file 41467_2018_5070_MOESM1_ESM.pdf]

Supplementary Information for

Role of disordered bipolar complexions on the sulfur embrittlement of nickel general grain boundaries

Tao Hu, Shengfeng Yang, Naixie Zhou, Yuanyao Zhang & Jian Luo (University of California, San Diego), *Nature Communications* 2018
Correspondence should be addressed to J. Luo (email: jluo@alum.mit.edu)

Table of Contents

Supplementary Note 1: Summary of the key experimental observations (*Supplementary Figs. 1-4*) 3
Supplementary Note 2: Summary of the key simulation results (*Supplementary Figs. 5-9*) 8
Supplementary Note 3: Indexing grain boundary (GB) terminal planes (*Supplementary Fig. 10*) 14
Supplementary Note 4: Comparison of different imaging modes: HRTEM, STEM HAADF *vs.* STEM ABF imaging (*Supplementary Fig. 11*) 16
Supplementary Note 5: EDXS measurements of the GB adsorption of S (*Supplementary Fig. 12*) 17
Supplementary Note 6: EELS mapping (*Supplementary Fig. 13*) 19
Supplementary Note 7: GB edge-on conditions and verification with a through-focus series of ABF images (*Supplementary Figs. 14-15*) 20
Supplementary Note 8: FFT filtering of pair STEM HAADF/AFB images (for Fig. 3f & Fig. 3g in the main article only) 22
Supplementary Note 9: Testing and calibration of the first-principles derived reactive force field (ReaxFF) potential for Ni-S (*Supplementary Fig. 16*) 23
Supplementary Note 10: Selection of model GBs for hybrid MC/MD simulation 24
Supplementary Note 11: Solubilities of S in Ni based FCC phase and alloy compositions 26
Supplementary Note 12: An exemplar to verify the thermodynamic equilibrium of Type A (amorphous-like) IGFs (*Supplementary Fig. 17*) 27
Supplementary Note 13: Analyses of partial structural orders in STEM images of Type A (amorphous-like) IGFs (*Supplementary Figs. 18-20*) 29
Supplementary Note 14: Intergranular fracture of S-doped Ni specimens (*Supplementary Table 1 and Fig. 21*) 32
Supplementary Note 15: Adsorption and excess disorder *vs.* $\Delta\mu$ for Type A *vs.* Type B GB facets (*Supplementary Figs. 22-23*) 34
Supplementary Note 16: Bond analysis, polar Ni-S structures, and bipolar interfacial structures (*Supplementary Fig. 24*) 37
Supplementary Note 17: Tensile properties from MD testing (*Supplementary Figs. 25-26*) 38
Supplementary Note 18: Further discussion of GB embrittlement and computed tensile toughness 40
Supplementary Note 19: Kikuchi patterns and measured GB disorientation angles (*Supplementary Table 2 and Fig. 27*) 42
Supplementary Note 20: Three types of GBs: critical comparisons of simulations and experiments (*Supplementary Table 3*) 44
Supplementary Note 21: Beyond embrittlement: abnormal grain growth in Ni-S 46
Supplementary Note 22: Summary of TEM/STEM observations (*Supplementary Figs. 28-31*) 47
Supplementary Note 23: Additional TEM/STEM images of individual observations: a case-by-case list (*Supplementary Figs. 32-40*) 51
Supplementary Note 24: Additional simulation results (*Supplementary Figs. 41-44*) 60
Supplementary Note 25: SEM micrographs of fractured faceted grain surfaces (*Supplementary Figs. 45-48*) 64
Supplementary References 68

Supplementary Note 1: Summary of the key experimental observations

In this study, we examined 34 independent GB facets in 10 randomly-selected boundaries in seven samples annealed at four different conditions, where:

(A) Eighteen independent GB facets all have one lower-index terminal grain surface of the (100) plane with another higher-index matching grain surface: all 18 facets are Type A (amorphous-like).

(B) Eleven GB facets (and 14 independent locations) have one lower-index grain surface of the (310), (311), (211), or (110) plane with another higher-index matching grain surface: all 11 facets are Type B (bilayer-like).

(C) Five GBs are nominally "clean" (Type C): four are Σ3 (111)//(111) symmetric twin boundaries and another is a low-angle GB.

The results are summarized in Table 1 in the main article and Supplementary Fig. 1. Additional TEM/STEM images of all 34 independent GB facets are documented in Supplementary Notes 22-23 and Figs. 28-40.

A representative Type A amorphous-like intergranular film (IGF) with a uniform nanometer thickness is shown in Supplementary Figs. 2 and 3 further illustrates that the amorphous-like IGFs formed on a parallel set of Type A facets exhibit a nanoscale thermodynamically-determined or "equilibrium" thickness of little variation from facet-to-facet (instead of an arbitrary thickness for a wetting film), as shown in Supplementary Fig. 3B (with additional images in Supplementary Fig. 17). See further elaboration in Supplementary Note 12.

Moreover, the interfacial energy of a nanometer-thick, liquid-like IGF is less than that of two crystal-liquid interfaces ($\gamma_{gb} \equiv \gamma_{IGF} < 2\gamma_{cl}$), because the dihedral angle at the groove, where the liquid-like IGF meets the bulk liquid phase, is non-zero (Supplementary Figs. 3C and 3D).

Thus, they are a true interfacial phase that are thermodynamically two-dimensional, which are also termed as "complexion" to differentiate them from the thin layers of the wetting bulk (3-D) phases at the interfaces[1-3].

The stabilization of the Type A amorphous-like (liquid-like) IGFs on the (100) facets can be explained by the low liquid-Ni (100) interfacial energy, which was confirmed experimentally by the Wulff shape of the Ni crystals in contact with the S-rich liquid (Supplementary Fig. 4).

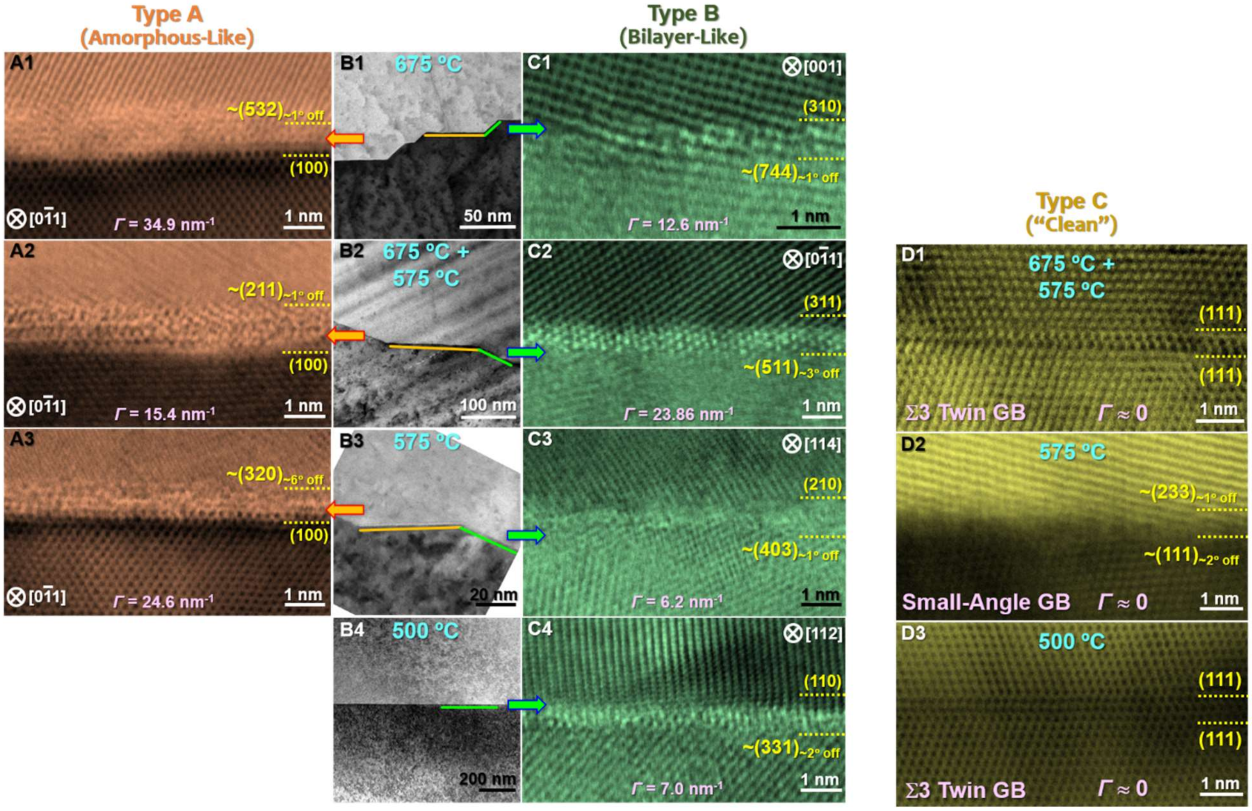

Supplementary Figure 1. Selected STEM ABF images of three types of characteristic GB structures. (A1, A2, A3) STEM images of representative Type A (amorphous-like) GB facets. **(B1, B2, B3, B4)** low-resolution images of the GBs equilibrated at four different temperatures. **(C1, C2, C3, C4)** STEM images of representative Type B GB facets. **(D1, D2, D3)** STEM images of two twin boundaries and a low-angle GB in S-doped Ni samples without detectable S adsorption (*i.e.*, Type C or nominally "clean" GBs). The measured GB excesses of S atoms (Γ) are labeled on the bottom of STEM images. Additional STEM images of all 34 GB facets examined can be found in Supplementary Figs. 28-40.

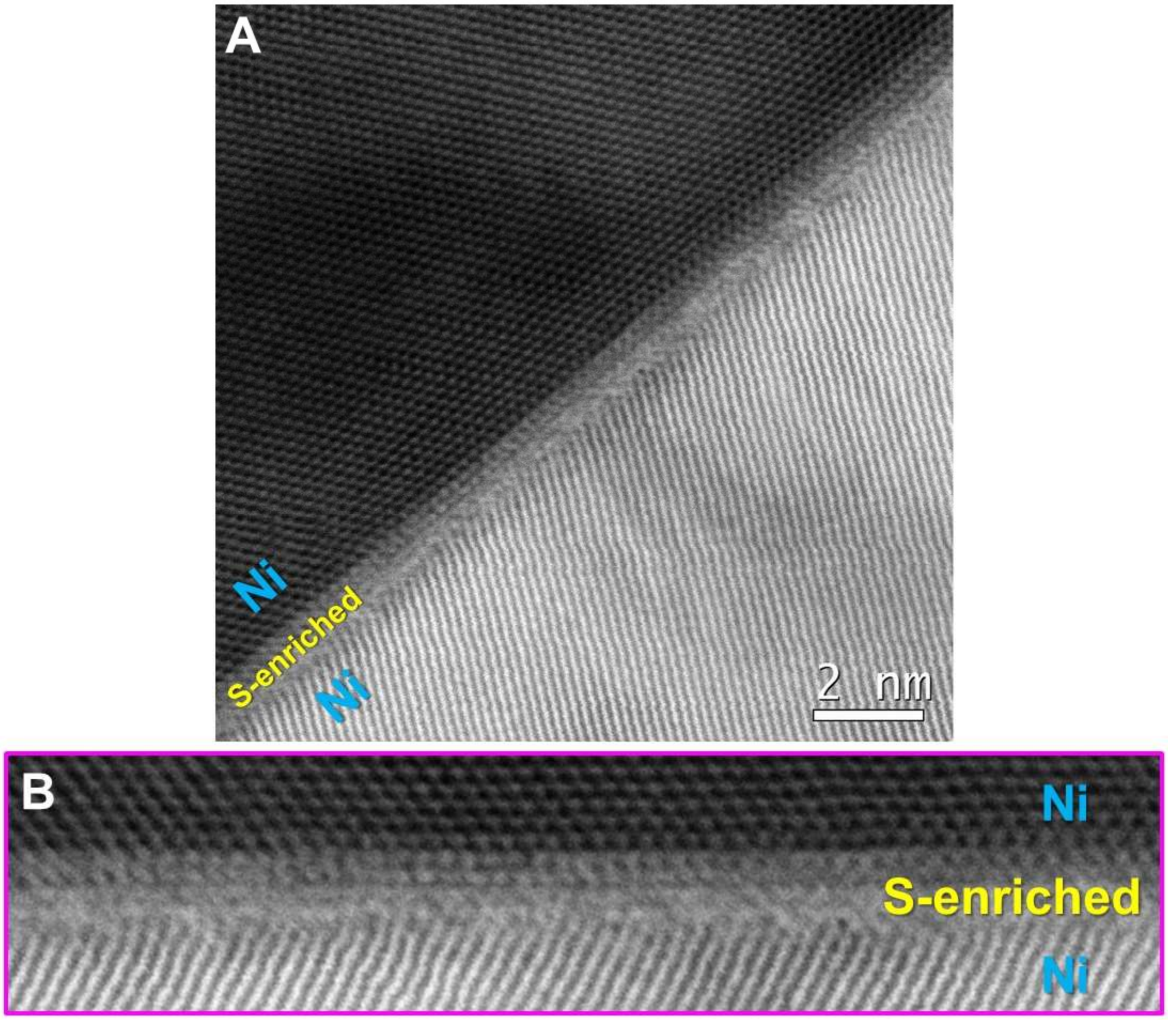

Supplementary Figure 2. Atomic resolution STEM ABF image of a Type A GB facet, which is a nanometer-thick, amorphous-like, intergranular film (IGF, yet exhibiting partial structural order). This specific GB was randomly selected from a sample quenched from 675 ºC. **(A)** STEM ABF image showing a Type A GB facet. The mean width (± one standard deviation from multiple measurements along the GB) of this interfacial phase (*i.e.,* equilibrium-thickness intergranular film) was measured to be 0.98 ± 0.15 nm. **(B)** is an enlarged segment of the Type A amorphous-like IGF shown in panel (A). See other examples of the Type A complexions (amorphous-like IGFs) in Fig. 2 and Fig. 3 in the main article and Supplementary Figs. 1, 3, 17, 28, 29, 32-35 and 37.

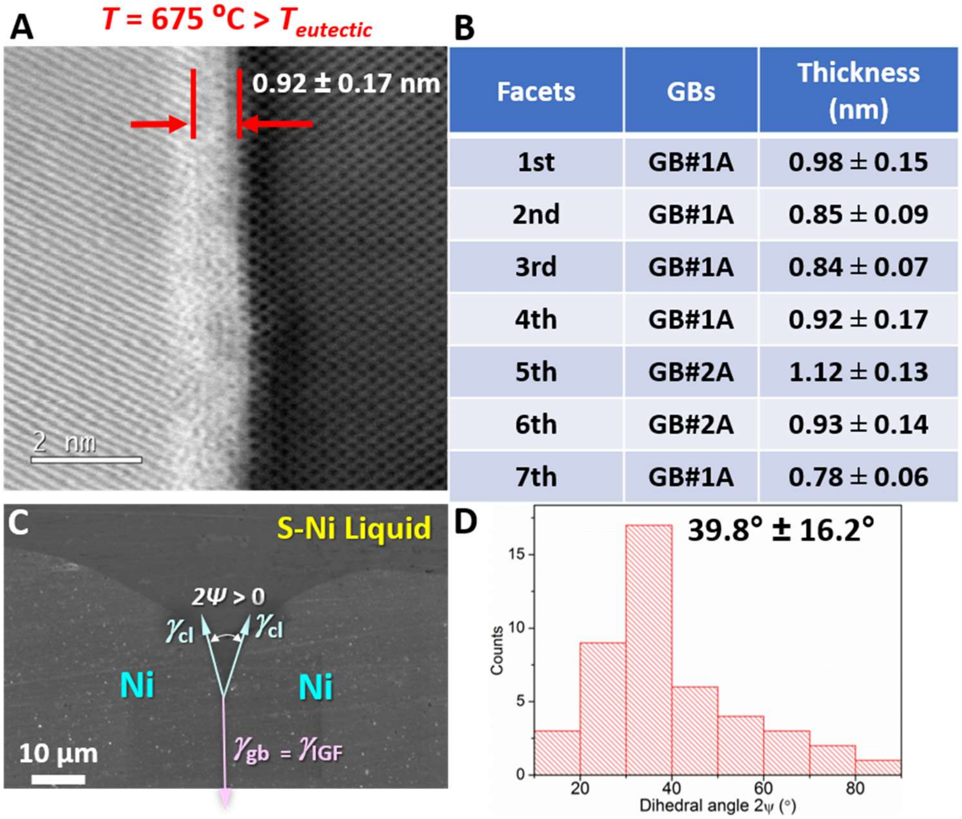

Facets	GBs	Thickness (nm)
1st	GB#1A	0.98 ± 0.15
2nd	GB#1A	0.85 ± 0.09
3rd	GB#1A	0.84 ± 0.07
4th	GB#1A	0.92 ± 0.17
5th	GB#2A	1.12 ± 0.13
6th	GB#2A	0.93 ± 0.14
7th	GB#1A	0.78 ± 0.06

Supplementary Figure 3. Uniformity and (narrow) distribution of the measured thicknesses of the Type A complexion, *i.e.*, equilibrium-thickness, amorphous-like, intergranular films (IGFs), and the associated non-zero dihedral angles, suggesting the formation of a true (thermodynamically 2-D) interfacial phase (instead of thin wetting layers of a bulk 3-D phase). (A) Representative 0.92 ± 0.17 nm thick amorphous-like IGF observed in a S-doped Ni sample annealed and quenched from 675 °C (above the eutectic temperature, $T_{\text{eutectic}} \approx 650$ °C). **(B)** Measured thicknesses (± standard deviations) of the IGFs formed on seven different facets in two different TEM specimens. **(C)** Cross-sectional SEM micrograph showing the penetration of the S-Ni liquid into a GB with a non-zero dihedral angle 2Ψ. **(D)** Histogram of the dihedral angles measured at 45 triple junctions, where the measured average projected dihedral angle of 39.8° on cross sections should be identical to the mean of the true dihedral angles in 3-D (since the cross sections are random). These narrow thickness distributions, as well as the non-zero average dihedral angle, demonstrated that these IGFs exhibits a nanoscale thermodynamically-determined or "equilibrium" thickness (instead of an arbitrary thickness for a wetting film) and its interfacial energy is less than that of two crystal-liquid interfaces ($\gamma_{gb} \equiv \gamma_{IGF} < 2\gamma_{cl}$); thus, they are a true interfacial phase that are thermodynamically two-dimensional, which is also termed as "complexion" to differentiate it from a thin layer of a bulk (thermodynamically three-dimensional) phase at an interface.

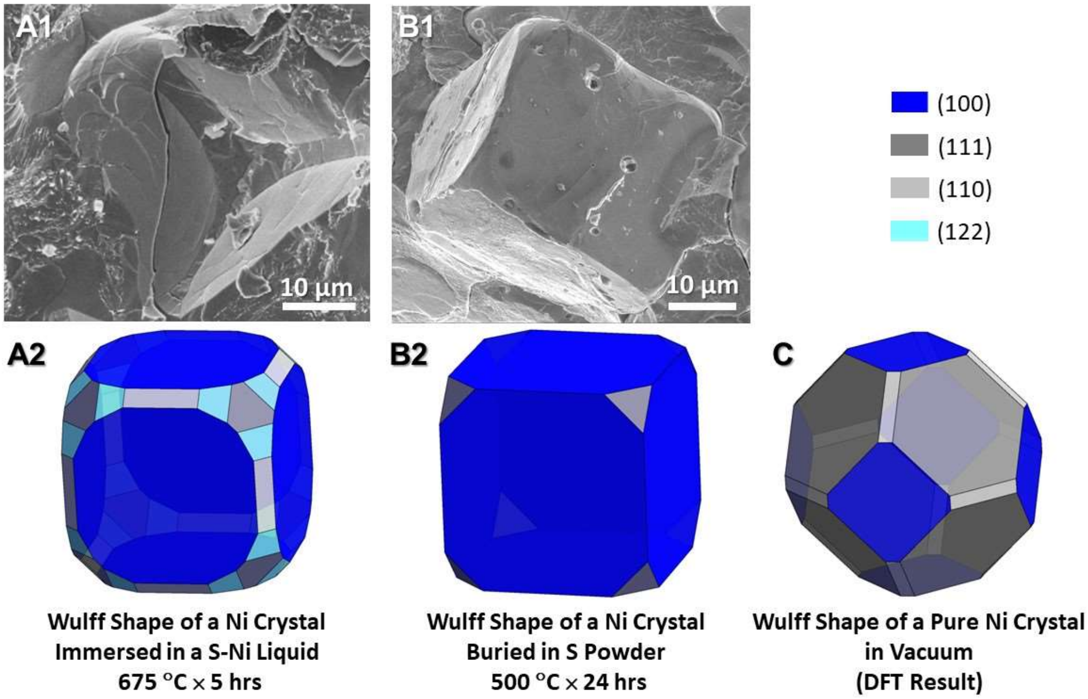

Supplementary Figure 4. Wulff shapes of Ni crystals saturated in S. Specimens were made by mixing Ni crystals (powder) with S, annealing at 675 °C (> T_{eutectic} = 650 °C) and 500 °C (< T_{eutectic}), respectively, followed by quenching. **(A1)** SEM images of nearly cubic Ni crystals that were equilibrated by immersing them in an equilibrium S-Ni liquid at 675 °C. **(B1)** SEM images of nearly cubic Ni crystals that were equilibrated by burying them in S powder at 500 °C. **(A2)** Wulff shapes of the S saturated Ni crystals at 675 °C. **(B2)** Wulff shapes of the S saturated Ni crystals at 500 °C. The shapes in A2 and B2 that match the SEM images shown in panels A1 and B1. **C**, Wulff shape of a pure Ni crystal in vacuum [from DFT calculations reported in Tran *et al.*, *Scientific Data*, 2016, 160080]. These results suggest that surface adsorption of S stabilizes (100) facets of Ni. Moreover, the interface between Ni (100) crystalline facets and the equilibrium S-enriched liquid should have the lowest interfacial energy, thereby promoting the formation of Type A GB complexions on (100) facets, *i.e.*, stabilizing S-enriched, nanometer-thick, liquid-like (amorphous-like), interfacial phases on (100) facets both above and below the bulk eutectic temperature.

Supplementary Note 2: Summary of the key simulation results

We simulated the equilibrium interfacial structures of the Type A, B, and C GB facets in semi-grand canonical ensembles. The simulated GB interfacial structures are critically compared to the experimental results in terms of the excess adsorptions of S, interfacial width, the level of structural disorder, and the simulated STEM images (Fig. 3 and Fig. 4 in the main article; Supplementary Figs. 5, 42 and 43; Supplementary Table 3).

Supplementary Fig. 5 illustrates a comparison of the GB interfacial structures obtained from the simulation and the experiments for one Type A GB and two Type B GBs. Additional modeling-experimental comparisons are presented in Fig. 3 and Fig. 4 in the main article and in Supplementary Fig. 42 and 43. The critical modeling-experimental comparisons are summarized in Supplementary Table 3.

From the simulation results, we identified polar S-Ni clusters (defined in "Methods" and further discussed in Supplementary Note 16), as illustrated in Supplementary Fig. 6. We also discovered the bipolar distributions of the polar S-Ni clusters (Supplementary Figs. 6D and 8).

We further conducted an MD tensile simulation on the pure and S-doped Ni GBs of Type A and B (Supplementary Fig. 7; elaboration in Supplementary Note 17). We found that the S-doped Ni GBs exhibit a brittle fracture, whereas the dislocations initiate and propagate in the pure Ni GBs.

Supplementary Fig. 8 shows the evolution of the atomistic structures of a Type A and B GB during the tensile simulation. The evolutions of the polar S-Ni clusters indicate that the fractures of the S-doped Ni GBs are caused by the separation among those polar clusters that disorderly aligned in opposite directions (S-to-S) (*i.e.*, with bipolar distributions of the $\vec{V}_{\mathrm{sum}}^{\mathrm{S}\rightarrow\mathrm{Ni}}$ vectors defined in Supplementary Fig. 6). The polar S-Ni clusters remain mostly intact after the fracture in the Type A and B GBs, as shown in Supplementary Fig. 8 and Fig. 5 in the main article. Further illustrations and discussion about the polar Ni-S clusters and their bipolar distributions can be found in Supplementary Note 16.

By changing the chemical potential of S in the semi-grand canonical ensemble atomistic simulations, we modeled the equilibrium GB interfacial structures with different amounts of S adsorption at Ni GBs. Supplementary Figs. 9A and 9B show a comparison of GB adsorption and the structural disorder of the GB structures obtained at different chemical potential differences for the (100)//(926) Type A GB and the (110)//(345) Type B GB.

Moreover, the tensile simulations showed that the computed tensile toughness (see Supplementary Note 17 for definition and discussion) decreases with the increasing bipolar index β (defined in "Methods") in both Type A and B GBs (Supplementary Figs. 9C and 9D). Thus, the formation of bipolar interfacial structures induced by the S adsorption causes brittle intergranular fractures between the polar S-Ni structures disorderly aligned in opposite directions, through a GB embrittlement (GBE) mechanism that is also displayed visually in Supplementary Fig. 8, as well as Fig. 5 in the main article.

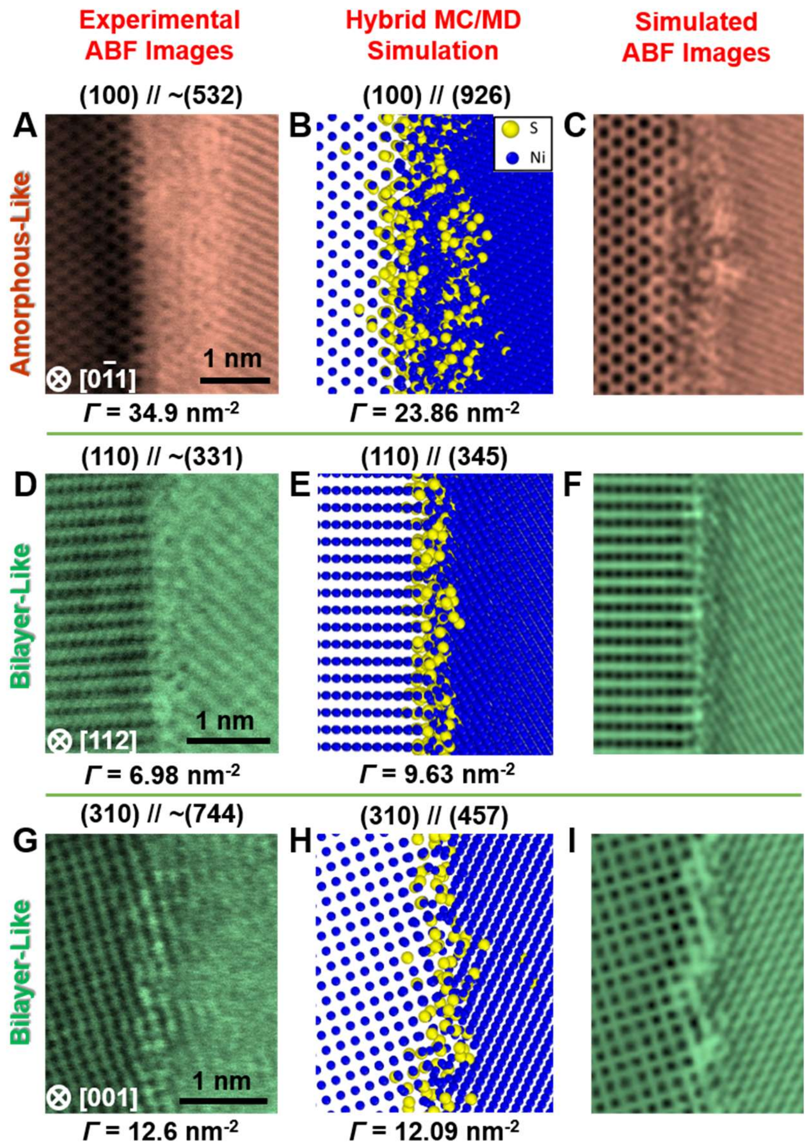

Supplementary Figure 5. Comparison of GB interfacial structures obtained from experiments and modeling. Atomic resolution STEM ABF images of **(A)** one Type A (amorphous-like) and **(D, G)** two Type B (bilayer-like) GBs. **(B, E, H)** Snapshots of interfacial structures obtained by semi-grand-canonical-ensemble hybrid MC/MD simulations of the equilibrium atomic structures of three model GB facets that resemble those observed in experiments. Specifically, (100), (110), and (310) planes are selected based on the TEM observations, whereas (926), (345), and (457) planes are selected to ensure periodic conditions to enable simulations; since STEM observations suggest that the low-index facet determines the general character of the interfacial structures, comparisons between modeling results and experiments can be made. The computed and experimentally-measured excess adsorptions of S (Γ) for each case are indicated, which agree well with each other in general. **(C, F, I)** Simulated STEM ABF images using the atomic structures generated by the hybrid MC/MD simulation shown in panels (B, E, H). The additional modeling-experimental comparisons (with additional simulation results) are summarized in Supplementary Table 3 and further discussed in Supplementary Note 20.

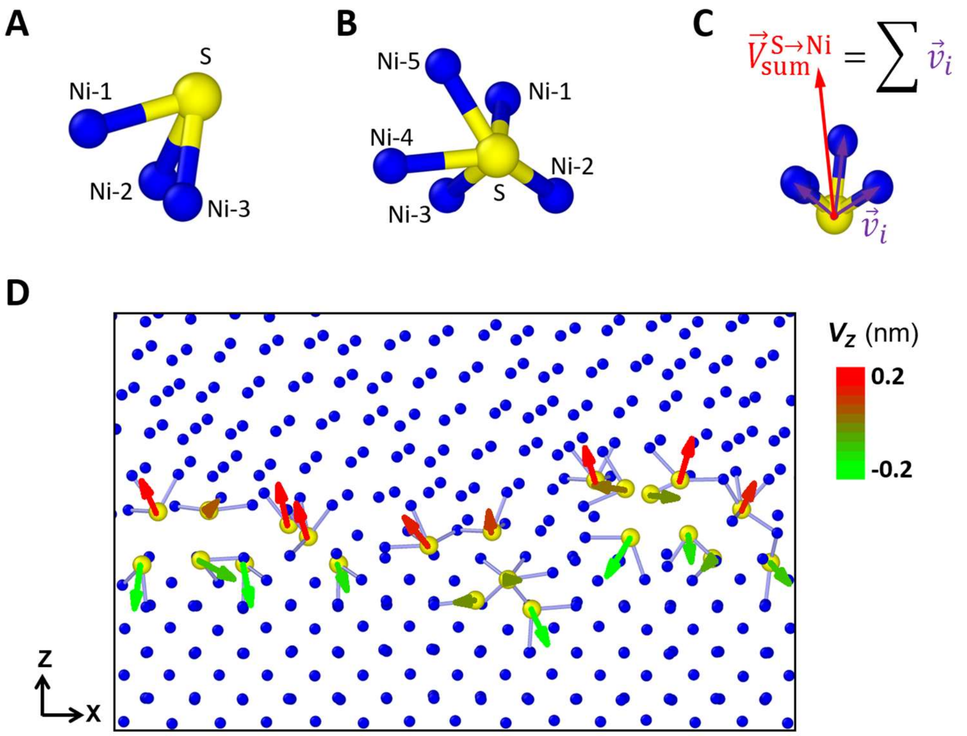

Supplementary Figure 6. Schematic illustrations of polar S-Ni clusters and bipolar interfacial structures that lead to GB embrittlement. (A, **B)** Two typical polar S-Ni structures found in S-doped Ni GBs. **(C)** Definition of the $\vec{V}_{\mathrm{sum}}^{\mathrm{S\to Ni}}$ vector in a polar S-Ni structure. **(D)** A representative snapshot of a bipolar distribution of polar S-Ni structures, where the color contour shows the *z* component of the $\vec{V}_{\mathrm{sum}}^{\mathrm{S\to Ni}}$ vector on each S atom.

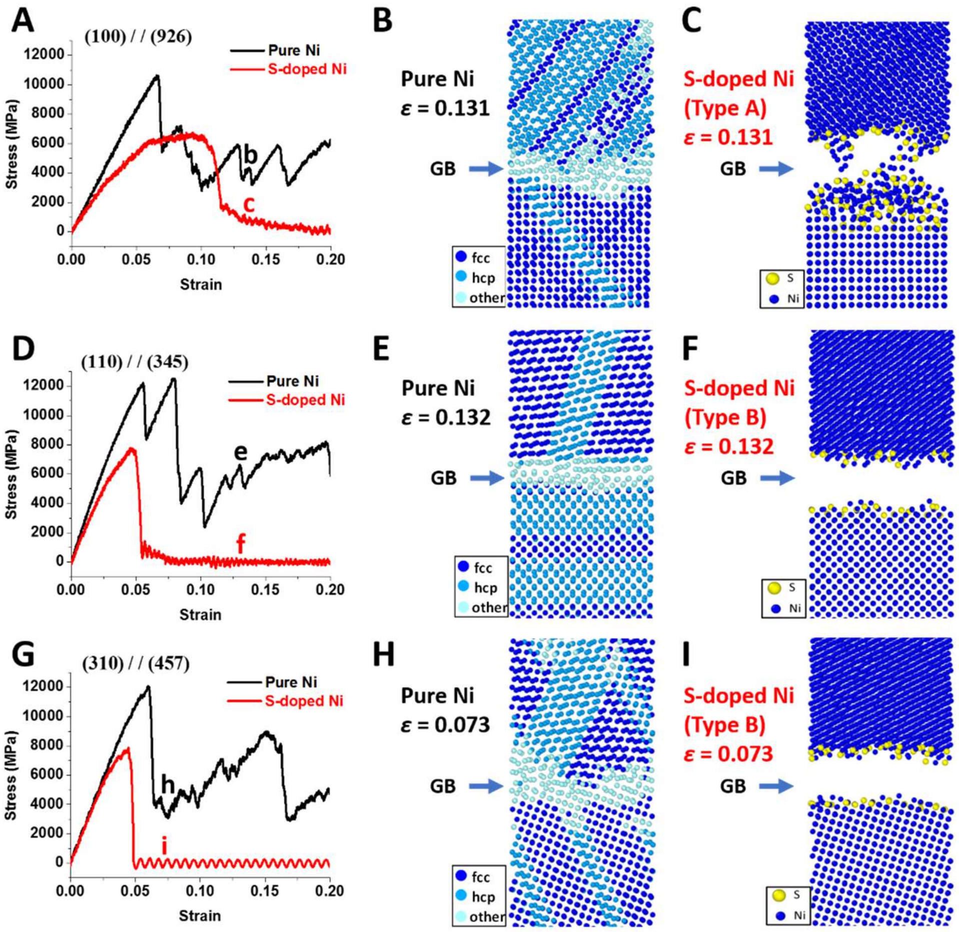

Supplementary Figure 7. Representative MD tensile simulations of GBs in pure and S-doped Ni. **(A, D, G)** Stress-strain curves for tensile testing of (100)//(926), (110)//(345), and (310)//(457) GBs, respectively. **(B, E, H)** Snapshots of pure Ni GBs during tensile, where the atoms are color-coded according to common neighbor analysis, showing ductile deformation. **(C, F, I)** Snapshots of S-doped Ni GB during tensile testing, where the blue and yellow dots represent Ni and S atoms, respectively, showing brittle intergranular fracture.

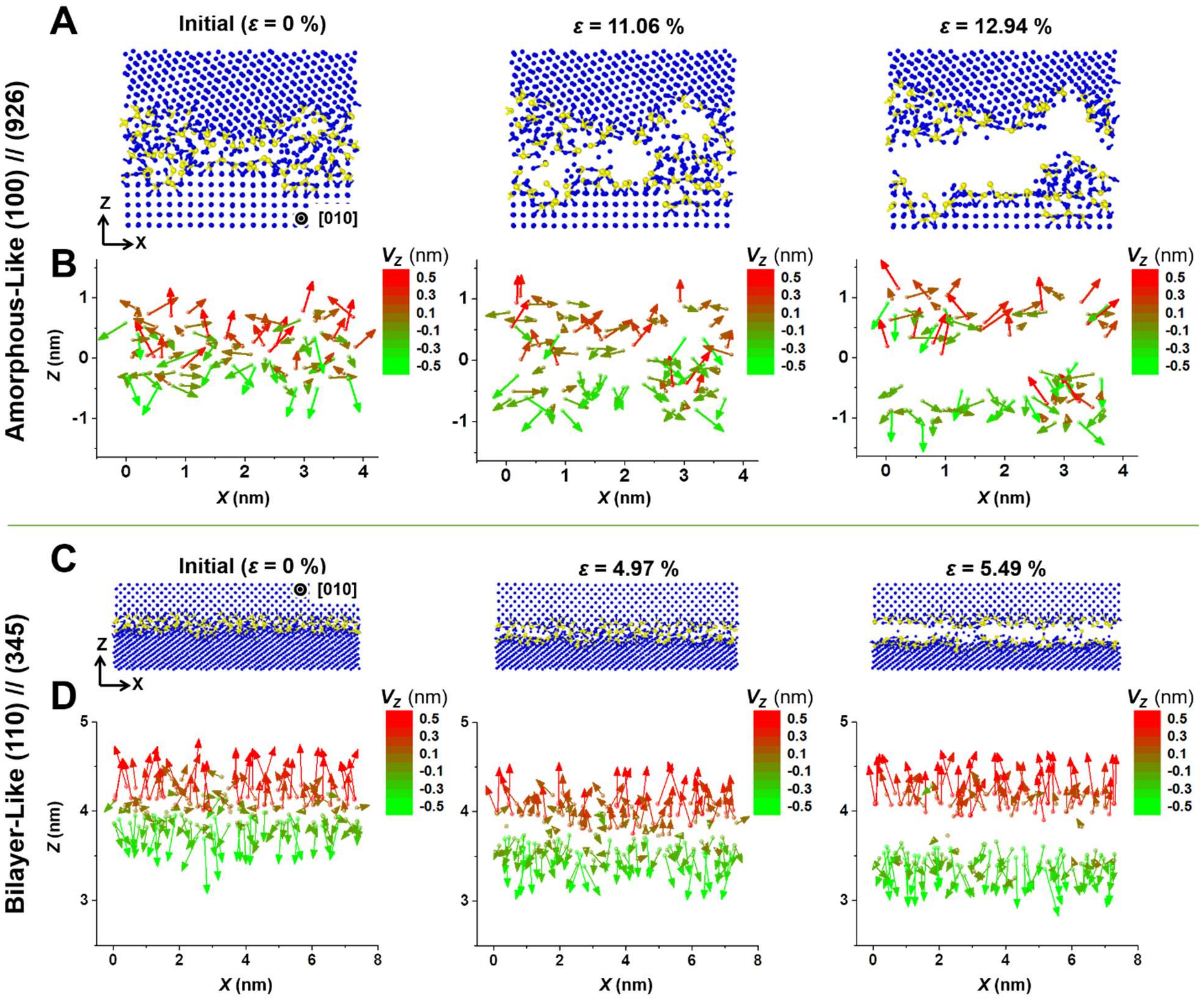

Supplementary Figure 8. Evolution of GB structures during tensile simulations of a Type A (100)//(926) GB facet and a Type B (110)//(345) GB facet. (A, C) Close-ups of the GB atomic structures under three different strains. **(B, D)** Distributions of $\vec{V}_{\mathrm{sum}}^{\mathrm{S}\rightarrow\mathrm{Ni}}$ vectors that represent the polar S-Ni structures, suggesting that bipolar interfacial structures lead to brittle intergranular factures. The arrows show the actual $\vec{V}_{\mathrm{sum}}^{\mathrm{S}\rightarrow\mathrm{Ni}}$ vectors pointed from each S atom, projected in the *x*-*z* plane. The color contour shows the *z* component of the $\vec{V}_{\mathrm{sum}}^{\mathrm{S}\rightarrow\mathrm{Ni}}$ vector on each S atom.

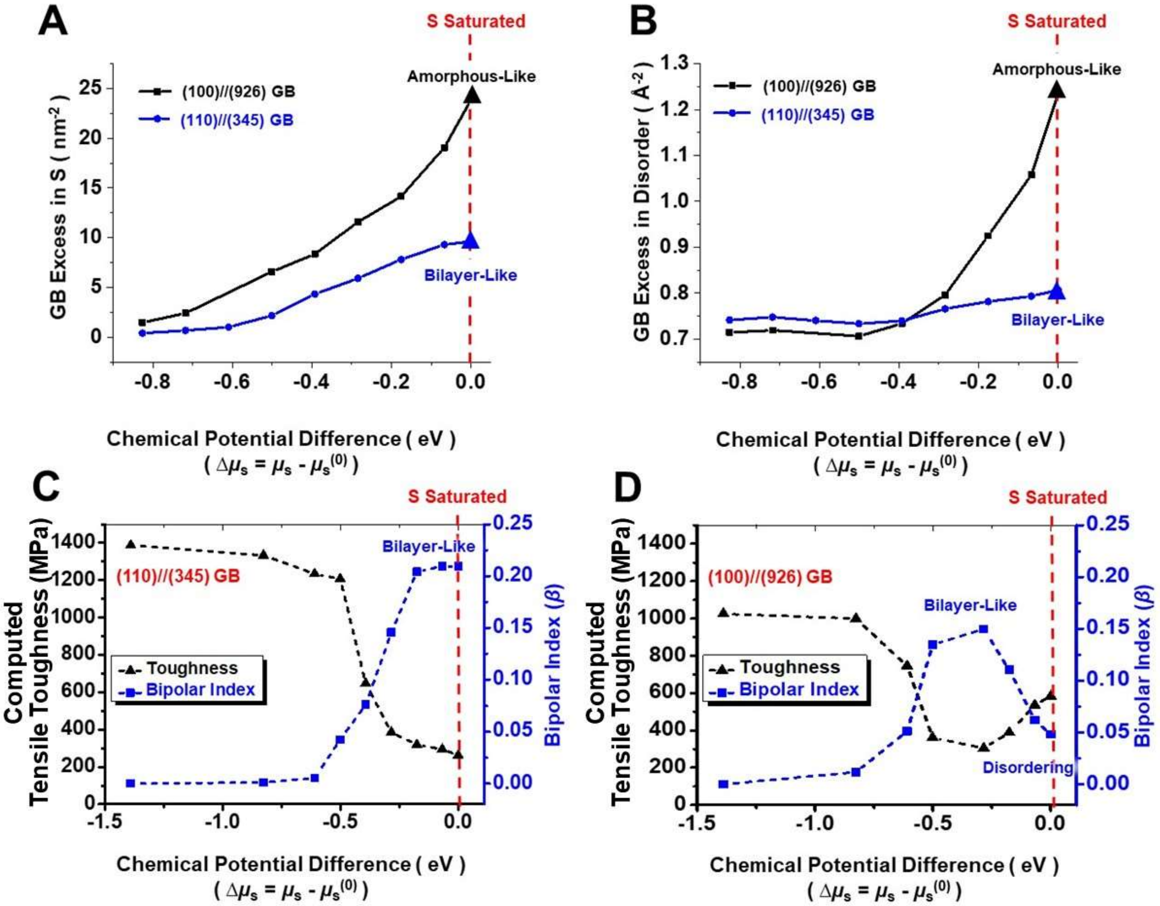

Supplementary Figure 9. Comparison of GB adsorption, disordering, bipolar (order) index β, and computed tensile toughness (defined in Supplementary Note 17) *vs.* S chemical potential curves for a (110)//(345) GB facet and a (100)//(926) GB facet. (A) Curves of GB excess in S *vs.* chemical potential difference of sulfur ($\Delta\mu_s \equiv \mu_s - \mu_s^{(0)}$, where $\mu_s^{(0)}$ is the sulfur chemical potential of the S-saturated FCC Ni phase). **(B)** Curves of GB excess in disorder *vs.* $\Delta\mu_s$. **(C)** Curves of bipolar (order) index and (GB fracture) toughness obtained by MD simulations *vs.* $\Delta\mu_s$ for the (110)//(345) GB facet. **(D)** Curves of bipolar (order) index β and computed tensile toughness obtained by MD simulations *vs.* $\Delta\mu_s$ for the (100)//(926) GB facet. Noting the bipolar (order) index β decreases after the (100)//(926) GB becomes disordering when $\Delta\mu_s >\sim -0.2$ eV, which correlates well with the GB excess disorder shown in (B). The corresponding equilibrium Type A and B GB structures at the experimental conditions (in S-saturated Ni specimens) are indicated by the large triangle symbols and vertical red dashed lines. The hybrid MC/MD simulations were conducted at T/T_M = 0.549 to simulate the Type A (100)//(926) GB facet and T/T_M= 0.447 to simulate the Type B (110)//(345) GB facet, respectively, corresponding to the experimental temperatures of 675 ºC and 500 ºC, respectively.

Supplementary Note 3: Indexing grain boundary (GB) terminal planes

To determine the crystal orientation of the two terminal planes of a grain boundary (GB), we used a lattice imaging method. Once the grain was tilted into the low-index zone axis, the terminal planes could be unambiguously indexed.

For example, to index the two terminal planes of the long faceting plane as shown in Supplementary Fig. 10, *Grain 1* was first tilted into [110] zone axis (and set to an edge-on condition). An atomic resolution lattice image was thus obtained. Based on the fast Fourier transformation (FFT) pattern shown in the inset of Supplementary Fig. 10B, three lattice planes were indexed to be $(11\bar{1})$, $(1\bar{1}1)$ and (200), respectively. Thus, the terminal plane for *Grain 1* was exactly the (200) plane. Similarly, we tilted *Grain 2* (on the other side of the same GB) into $[11\bar{4}]$ zone axis. The atomic resolution lattice image and corresponding FFT pattern were obtained and are shown in Supplementary Fig. 10C. Three planes corresponding to the diffraction spots could thus be determined as $(31\bar{1})$, $(1\bar{3}\bar{1})$ and $(\bar{2}20)$, respectively. The terminal plane of *Grain 2* was then calculated based on three vectors to be close to (532) with 1° off. The two parallel terminal planes of this facet were determined to be (200) and (532). Therefore, the orientation relationship for this GB was (200)//~(532). This orientation relationship is labeled as (100)//~$(532)_{\sim 1^\circ\ \mathrm{off}}$, which means that the actual plane on the right side is 1° off the exact (532) plane; noting that (200) lattice fringes in TEM represent the crystalline (100) planes of the FCC crystal. To maintain consistency, we always kept the lower Miller index, *e.g.,* (100) and (110), on the left.

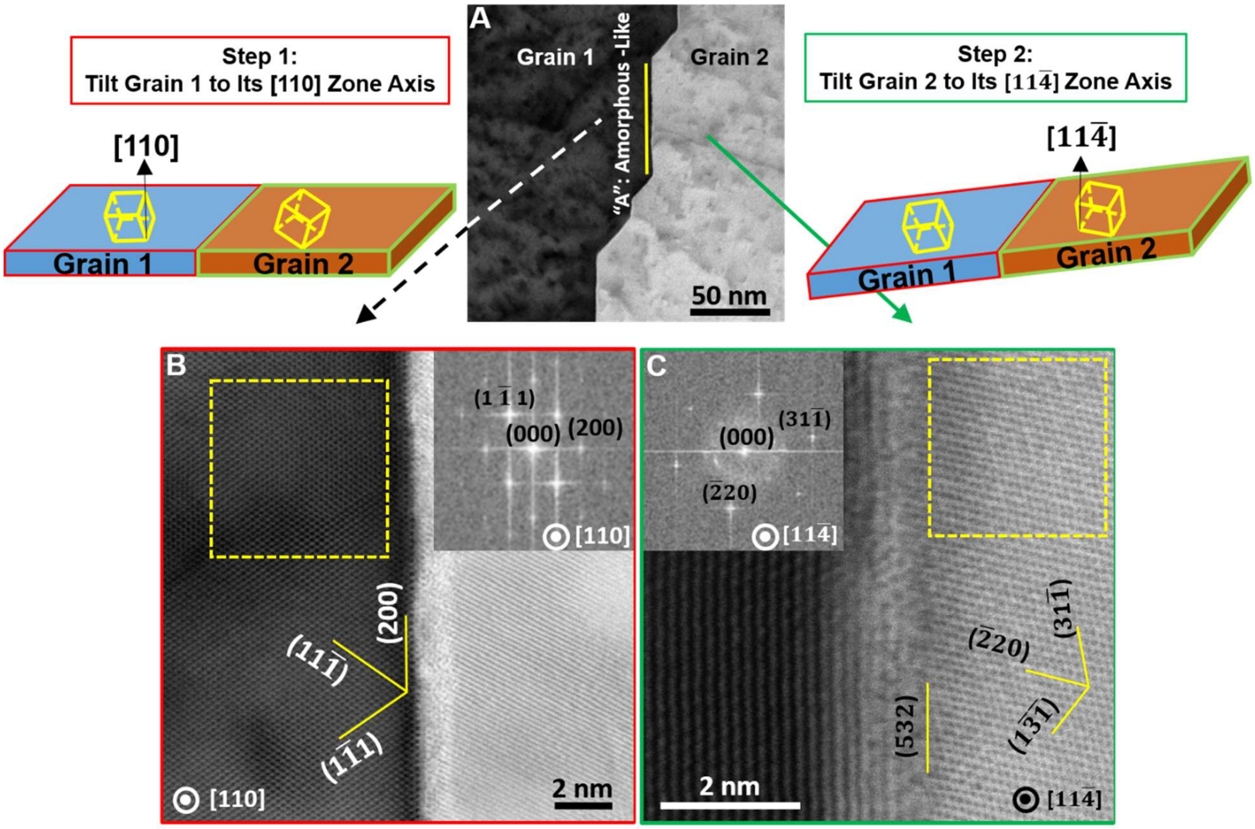

Supplementary Figure 10. Determination of the orientation relationship of a GB by using a lattice imaging method. An example of a GB in S-doped Ni annealed at 675 ºC. **(A)** STEM image showing that the GB exhibits two types of facets. The two adjacent crystals were labeled as *Grain 1* and *Grain 2*. **(B)** In Step 1, we tilted *Grain 1* to its [110] zone axis, which was also an edge-on condition. An atomic-resolution lattice image was obtained and is shown in panel (b). The inset is the FFT pattern obtained from the selected area highlighted by the yellow frame. The FFT pattern was indexed as the standard diffraction pattern for the FCC structure in its [011] zone axis and the terminal plane of *Grain 1* on the GB was determined to be (200). **(C)** In Step 2, we tilted *Grain 2* to its [11$\bar{4}$] zone axis. An atomic resolution lattice image was then obtained and is shown in panel (c). The inset is the FFT pattern obtained from the selected area highlighted by the yellow frame. The FFT pattern was indexed as the standard diffraction pattern for the FCC structure in [11$\bar{4}$]. The terminal plane for *Grain 2* was determined to be approximately ~1° off the (532) plane. Therefore, the GB orientation relationship is $\{100\}_{\text{Grain 1}} // \sim \{532\}_{\text{Grain 2}}$, approximately 1° off, which is also denoted as "(100)//~(532)$_{\sim 1° \text{ off}}$". Here we use (100) to label the grain surface, instead of (200), which is typically used to label lattice fringes or a diffraction pattern; noting that (100) and (200) refer to the same crystalline surface orientation while (100) planes of an FCC lattice does not show up in diffraction because of the symmetry.

Supplementary Note 4: Comparison of different imaging modes: HRTEM, STEM HAADF *vs.* STEM ABF imaging

As shown in Supplementary Fig. 11, the amorphous-like and bilayer-like GB structures observed in this study cannot be clearly discerned in phase-contrast lattice imaging using conventional HRTEM imaging. Supplementary Figs. 11A1 and 11A2 are synchronized STEM ABF and STEM HAADF images of an amorphous-like GB facet. The conventional HRTEM image of the same GB is shown in Supplementary Fig. 11A3.

A similar pair of STEM ABF and HAADF images of a Type B GB facet, as well as the conventional HRTEM image of this Type B GB facet, were also taken and are shown in Supplementary Figs. 11B1, 11B2 and 11B3 for comparison. The application of STEM ABF enabled us to better visualize the interfacial structure composed of the light element sulfur, which is not clearly distinguished by conventional HRTEM and STEM HAADF. The adsorbed sulfur atoms on the GB facet, which was further confirmed by EELS and EDXS, gives the bright contrast in ABF images. In contrast, HAADF contrast is based on scattering amplitude and is known as atomic number Z contrast, whose sensitivity depends on the scattering power of the relevant atoms[4]. Thus, for detection of lighter atoms with extremely weak scattering, ABF phase-contrast imaging based on wave interference is preferred in the present case. This produces clearer atomic-resolution images because it requires that the atoms only alter the phase of a wave[5,6].

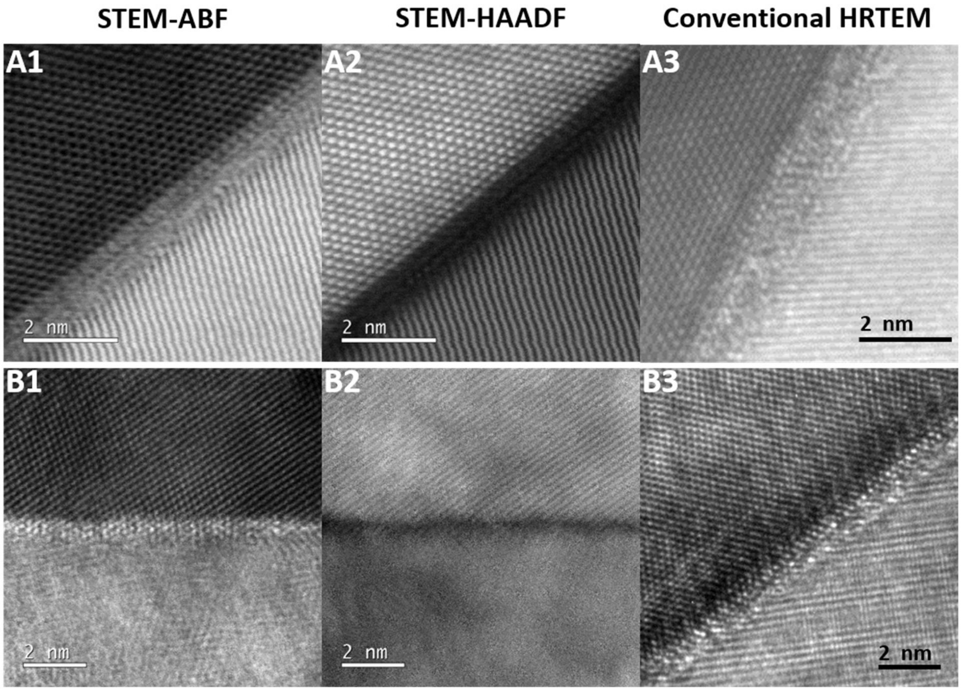

Supplementary Figure 11. Comparison of aberration-corrected STEM ABF, STEM HAADF and conventional HRTEM images of Type A and Type B GB structures. (A1-A3) A Type A GB facet or a nanometer-thick amorphous-like intergranular film (that was observed in a S-doped Ni sample treated at 675 ºC in this specific case). **(B1-B3)** A Type B GB facet (that was observed in a S-doped Ni sample pre-treated at 675 ºC and subsequently annealed at 575 ºC in this specific case). Here, (A1) and (A2), as well as (B1) and (B2), are a pair of synchronized STEM ABF and STEM HAADF images, respectively, while (A3), as well as (B3), is the conventional HRTEM image of the same GB facet. In both cases, STEM ABF images give better contrast and show the GB structures with the segregation of the light element S.

Supplementary Note 5: EDXS measurements of the GB adsorption of S

We used the energy dispersion X-ray spectroscopy (EDXS) based box scanning method (in STEM, JEOL ARM200F) to quantify GB excess adsorption of sulfur in the GBs. This was done by measuring the solute concentration in a well-defined volume containing GB, from which the excess concentration per unit area relative to the grain interior (as the reference) was determined. The details of this box scanning method can be found in Ref. 7 and is briefly explained with an example, as follows.

As illustrated in Supplementary Fig. 12A, the three identical rectangles represent the electron beam scanning area with a fixed size of 90.4 Å × 15.3 Å. The distance between the scanning area in the grain interior to the GB was fixed at 60 Å. Thus, the average thickness (scanned volume) of the scanned area in the grain interior should be similar to that of the GB. Each selected area was scanned by an electron beam with a spot size of 8C (the JEOL spot size number) for 70 seconds. The parameters of EDXS analysis were kept consistent for all the GBs in this study. Supplementary Fig. 12D shows the typical spectra collected from a Ni GB and adjacent grains.

The Cliff-Lorimer method was used to obtain relative concentrations of the elements.[8] Normalized to nickel, the sulfur concentrations are given by:

$$\frac{C_S}{C_{Ni}} = k_{SNi}\frac{I_S}{I_{Ni}} \ , \qquad (1)$$

where k_{SNi} is a calibration factor, and I_S and I_{Ni} are the integrated peak intensities for element sulfur and nickel, respectively. The database of calibration factors for this specific EDS detector in ARM200F was provided by the JEOL company. Taking the Si as a reference, the calibration factor is 1.783 for the Ni K line and 1.137 for the S K line. Thus, the calibration factor for the sulfur to the nickel is 0.64 and Eq. (1) can be rewritten as:

$$\frac{C_S}{1-C_S} = 0.64\frac{I_S}{I_{Ni}} \qquad (2)$$

By intergrading the peak intensities for the lines of interests ($S_{K\alpha}$, $Ni_{K\alpha}$) after background stripping, the S concentration can be obtained. The S concentration in the Ni grain interior is negligibly small because of the low solubility of S in Ni, which cannot be detected by EDXS. Therefore, the excess of S on the GB per unit area can be expressed as:

$$\Gamma_S = \frac{C_S^{excess}NV}{A} = \frac{C_S^{excess}Nw}{V_m}, \qquad (3)$$

where Γ_S is the GB excess of S per unit area, N is the atomic density of the S (0.065 mol/cm^3), V is the analyzed volume, A is the GB area, w is the width of the scanned area (15.3 Å in this study), and V_m is the molar volume (9.63 cm^3/mol).

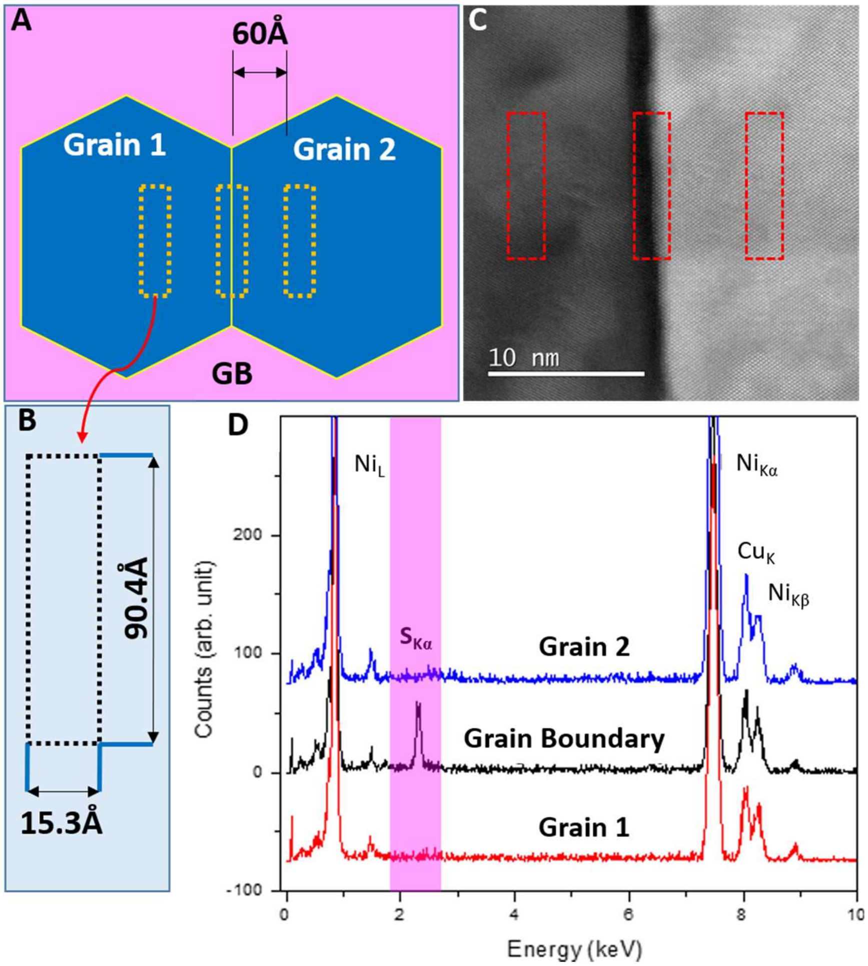

Supplementary Figure 12. Measurements of GB adsorption by energy dispersion X-ray spectroscopy (EDXS) in STEM. (A) Schematic diagram showing one scanning area/box on a GB and two scanning boxes in the adjacent grain interiors for a quantitative measurement of GB excess of solutes. The size and the position of these scanning boxes were fixed for all measurements. The duration of each box scanning was also fixed to be 70 s. **(B)** An enlarged sketch showing the size of the scanning box. **(C)** STEM image of a typical example of EDXS box scanning analysis of a GB in a S-doped Ni specimen treated at 675 ºC. **(D)** The corresponding EDX spectra obtained from the GB and the two adjacent crystalline grains in (c). The pink belt indicated the $S_{k\alpha}$ peak that is significant in the EDX spectrum obtained on the GB, while the EDX spectra from the two adjacent grain interiors have no detectable $S_{k\alpha}$ peak. The Cu_k peaks were from the TEM Cu grid. No detectable Ga peaks appeared, indicating that the FIB milling did not result in chemical contamination. The same EDXS analysis method and parameters were used for analyzing all the GBs in this study for consistency.

Supplementary Note 6: EELS mapping

Electron energy loss spectrum (EELS) was recorded using a Gatan Enfinium spectrometer (equipped on the ARM-200F STEM) with an energy resolution of (full-width at half maximum) ~0.5 eV. The S segregation at a Type A GB facet was analyzed by EELS mapping as shown in Supplementary Fig. 13. A nanometer-thick S-enriched region was evident in the EELS map, consistent with STEM and EDXS observations. The energy-loss range near the S $L_{2,3}$ edge shows the segregation of S at the GB. The energy-loss range near the Ni L_2 edge shows no significant change at the GB.

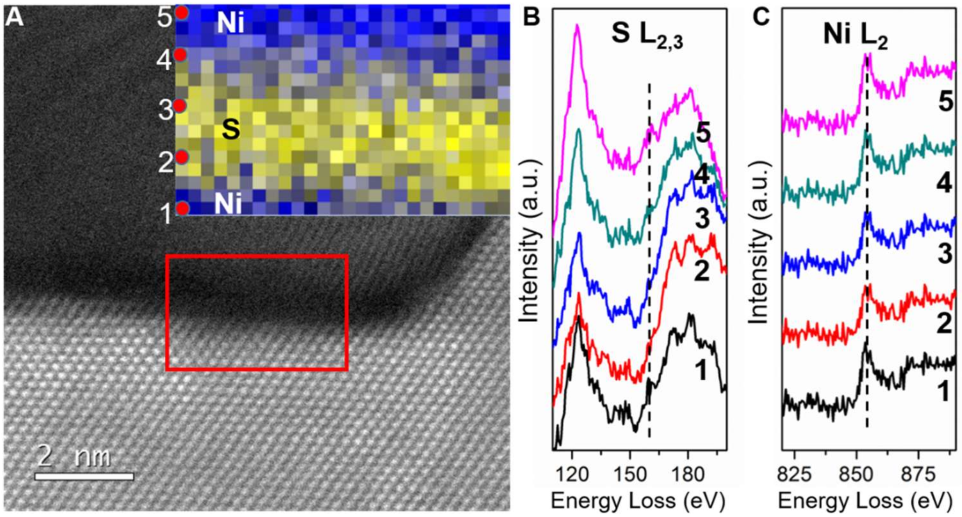

Supplementary Figure 13. Electron energy loss spectroscopy (EELS) analysis of a Type A GB facet. (A) STEM image of a faceted GB obtained by the annular detector in Gatan GIF. Inset is the EELS compositional map obtained from the area highlighted by the red rectangle. The intensity of yellow pixels represents the S distribution, while that of the blue pixels represents the Ni distribution. Five representative EELS spectra collected from the spots 1-5 labeled in the inset are shown. **(B)** Energy-loss range near the S $L_{2,3}$ edge showing the segregation of S at the GB. **(C)** Energy-loss range near the Ni L_2 edge showing no significant change at the GB.

Supplementary Note 7: GB edge-on conditions and verification with a through-focus series of ABF images

An "edge-on" condition for GBs is a requirement to clearly image the GBs in TEM/STEM. When a GB was set to an "edge-on" condition, the GB is parallel to the electron beam in the microscope. To ensure that the GB was set on an "edge-on" condition, we used a through-focus series of ABF micrographs to examine through the thickness of the TEM specimen.

An example is shown in Supplementary Fig. 14, where there was no significant change in thickness and the morphology for the amorphous-like GB when the focus varied from −6 nm to +6 nm, implying that the GB is edge-on. Another example is shown in Supplementary Fig. 15 for a bilayer-like GB, where the position of the GB did not shift when the defocus varies, indicating an "edge-on" condition.

The through-focus series of ABF micrographs also indicated that the GB structures are uniform in the through-thickness direction, and as such the STEM images truthfully represent the interfacial widths and structures in the projections.

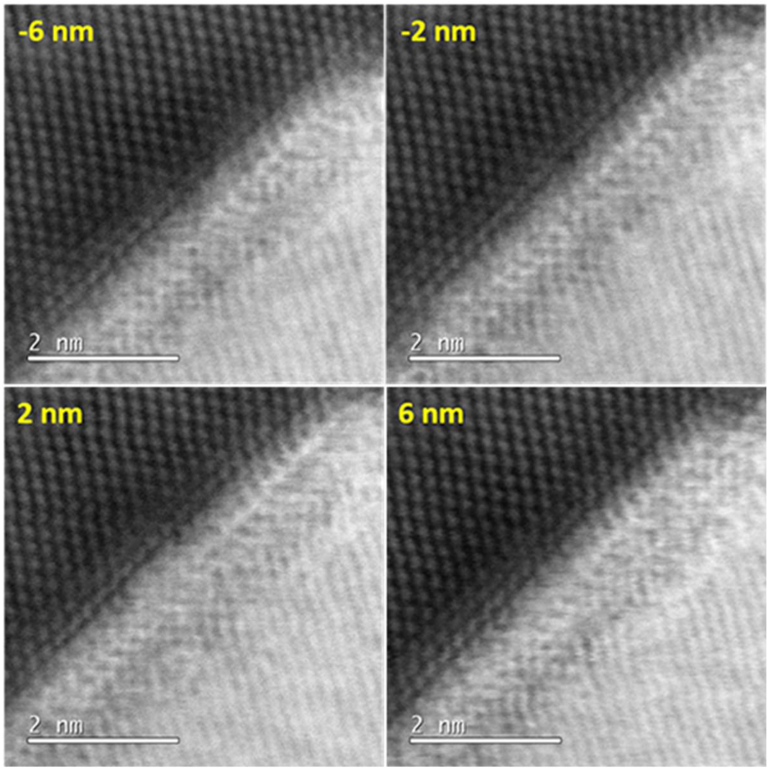

Supplementary Figure 14. A through-focus series of STEM ABF micrographs of a Type A (amorphous-like) GB facet. The defocus values are labeled. There is no significant change of the apparent interfacial width, indicating the uniformity of this nanometer-thick, amorphous-like, intergranular film throughout the specimen thickness direction.

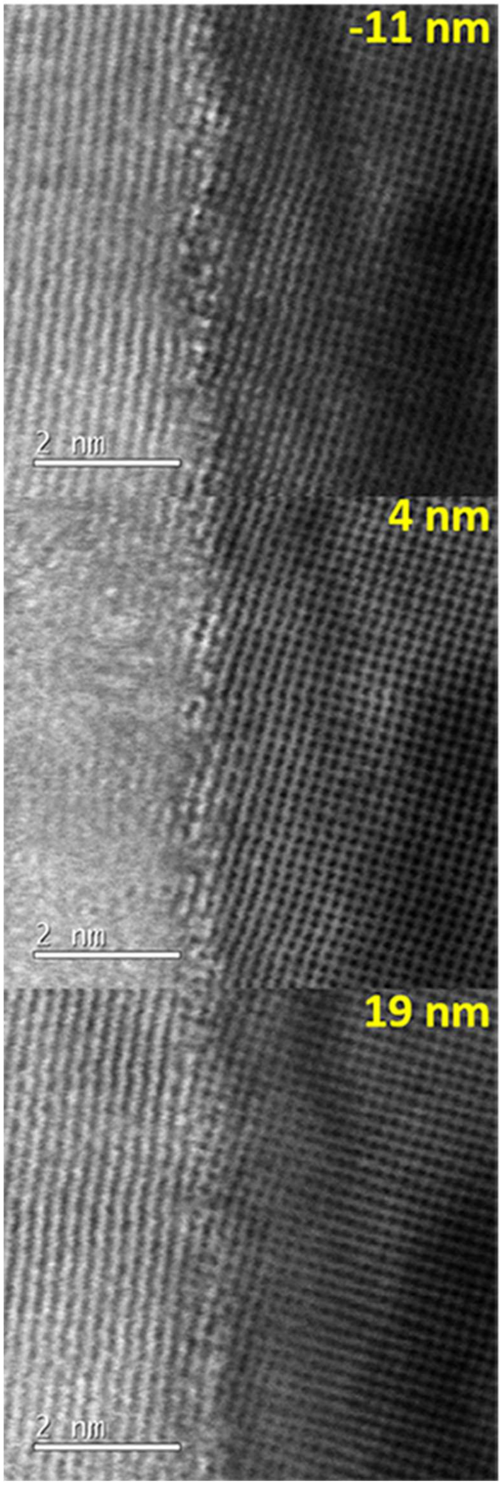

Supplementary Figure 15. A through-focus series of STEM ABF micrographs of a Type B GB facet. The defocus values were labeled. There is no significant change of the GB position and interface width/morphology, indicating that this GB facet is edge-on and it possesses the same character throughout the thickness direction.

Supplementary Note 8: FFT filtering of pair STEM HAADF/AFB images (for Fig. 3f & Fig. 3g in the main article only)

FFT filtering of STEM HAADF/ABF images (Fig. 3f and Fig. 3g in the main article) was implemented by performing FFT of the entire raw STEM image. The FFT pattern showed the periodic information of the lattice in the frequency domain. A concentric mask was applied to exclude the central spot and high frequency spots (while only first order spots were uncovered). The masked frequency information was then used to obtain the inverse FFT image.

FFT filtering was used to remove the background contrast to clearly show the partial structural orders in the amorphous-like GB structures.

We note that Fig. 3f and Fig. 3g in the main article are the only FFT filtered images reported (for the purpose of making the partial structural orders in the amorphous-like GB structures clearly visible); all other STEM and HRTEM images in the main article and the Supplementary Information are un-filtered images.

Supplementary Note 9: Testing and calibration of the first-principles derived reactive force field (ReaxFF) potential for Ni-S

The first-principles derived reactive force field (ReaxFF)[9] for Ni and S used in this work has been validated against experimental data and first-principles quantum-mechanical calculations based on the density functional theory at 0 K. In order to calibrate the ReaxFF for simulating S-doped Ni at high temperature, we first computed the melting temperature T_m of pure Ni using this ReaxFF. Pure molecular dynamics (MD) simulation (at a zero-pressure condition) was conducted on a supercell of pure Ni containing a solid-liquid interface. By calculating the volume of the supercell at various simulation temperatures, T_m was predicted to be 2227.5 K with an accuracy of ±5 K (Supplementary Fig. 16A). Therefore, all the simulated temperatures in this work were scaled based on the simulated melting temperature.

Subsequently, we used a hybrid Monte Carlo and molecular dynamics (hybrid MC/MD) method to determine the chemical potential difference that corresponds to the equilibrium condition (at each temperature), which should result in no motion of a solid-liquid interface. In hybrid MC/MD simulations, the total number of atoms and the chemical potential difference between Ni and S $\Delta\mu$ ($\equiv\mu_S - \mu_{Ni}$) were fixed while the number of S atoms was randomly varied. The direction of the solid-liquid interface motion was monitored by computing the rate of the volume change of the system as the chemical potential difference $\Delta\mu$ was varied in small increments at a fixed temperature. When $\Delta\mu$ was set to −0.474 eV, there was no motion of the solid-liquid interface observed at T = 1222.12 K (corresponding to a scaled temperature of 675 °C in the experiments), as shown in Supplementary Fig. 16B. At the liquid front, the equilibrium between solid and liquid. Subsequently, the same $\Delta\mu$ obtained from the solid-liquid equilibrium was used in simulating the equilibrium structure in S-doped Ni GBs.

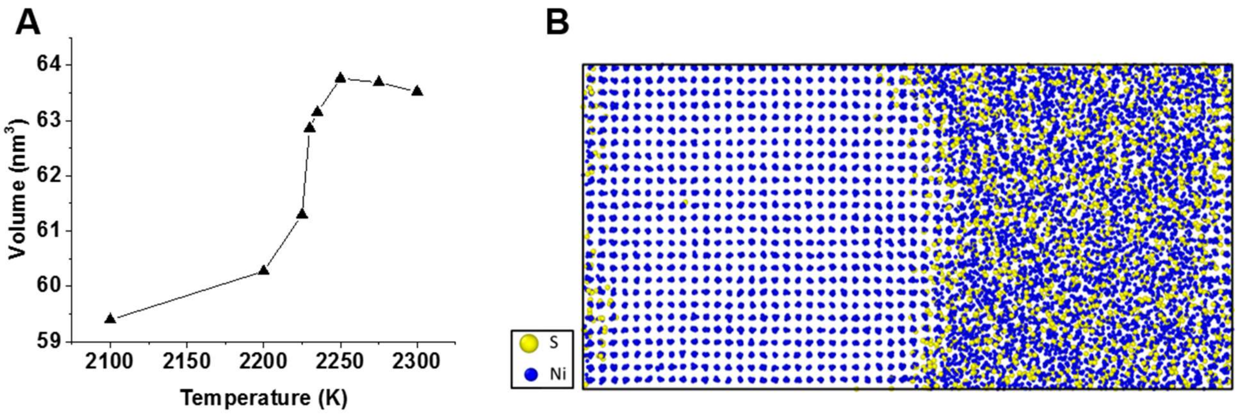

Supplementary Figure 16. Calculations of the melting temperature and equilibrium compositions of the Ni-S binary system using a first-principles derived reactive force field ReaxFF potential [9]. (A) The volume change of a supercell of pure Ni with increasing temperature, shows the melting of pure Ni at 2227.5±5 K with the ReaxFF potential. **(B)** A supercell with a solid-liquid interface that does not move at 1222.12 K; this and similar calculations were used to calculate liquidus composition to calibrate the ReaxFF potential.

Supplementary Note 10: Selection of model GBs for hybrid MC/MD simulation

Five GB models with different orientation relationships were created and simulated with hybrid MC/MD simulations to mimic the observed GBs in experiments. The sizes of the models must be significantly smaller than those in experiments to allow simulations in realistic timeframes. Periodic boundary conditions were used; thus, it is infeasible to exactly match the orientations of both the left and right grains observed experimentally nor adopt GBs of very large Σ values.

Fortunately, the experimental observations suggest that all GBs have one grain surface with a low Miller index that dictates the behavior of GBs (a hypothesis based on experimental observations, which has been further validated by modeling, as briefly discussed below and further elaborated in Supplementary Note 20).

Therefore, we selected the left grain of the GB model to match exactly that low-Miller-index plane observed in the experiment. For example, we selected Σ 11 (100)//(926) GB facet to represent Type A GB facets, and Σ 15 (110)//(345), Σ 3 (310)//(457), and Σ 11 (310)//($3\bar{1}0$) GB facets to represent Type B GB facets.

Simulation results discussed in Supplementary Note 20 further verified that the model GBs selected can indeed represent experiments, because the orientation of the lower-index grain terminating plane, instead of the misorientation, dictates the interfacial structures.

Specifically, the simulated results from model GBs agree well with experiments for complexions formed on lower index terminating surfaces of (100), (110) and (310) facets in terms of not only the types of complexions formed, but also the GB adsorption amounts of S and the levels of GB structural disorders (Supplementary Table 3; Fig. 3 and Fig. 4 in the main article; Supplementary Figs. 42 and 43).

A specific case to demonstrate that the orientation of the lower-index grain terminating plane, instead of the misorientation between the two abutting grains, dictates the interfacial behavior is represented by modeling results where both Σ11 (310)//($3\bar{1}0$) and Σ3 (310)//(457) facets exhibit Type B bilayer complexions with similar computed Γ's (12.2 and 12.1 S atoms per nm^2, respectively) that agree very well with the experimental observation of the Type B bilayer-like complexion at (310)//∼(744) with a measured Γ = 12.6 S atoms per nm^2. This supports our hypothesis based on the experiments (stated above) and justifying the validity of our approach to select model GBs. See Supplementary Note 20, Supplementary Table 3, Supplementary Figs. 42 and 43 for detail and further discussion.

We have further verified that the asymmetric GBs we selected have GB energies (γ_{GB}'s) that are comparable with those of general GBs.

- To confirm this, pure Ni GB models were created and relaxed at T = 300 K using MD simulations. The GB energies of these three models were obtained by first quenching them to T = 1 K and then calculating with the equation: $E_{GB} = (E_{all} - E_{bulk} * N_{Ni}) / A$, where E_{all} is the potential energy for the model including the GB, E_{bulk} is the potential energy for a Ni atom in the bulk, N_{Ni} is the number of Ni atoms, and A is the GB area. In order to compare the GB energy with results from the literature,[10] we used the same EAM potential [10] here to calculate the GB energy. The GB energies for the (100)//(926) GB, (110)//(345) GB, (310)//(457) GB, and (310)//($3\bar{1}0$) GB were computed to be 1.358 J/m^2, 1.314 J/m^2, 1.345 J/m^2, and 1.308 J/m^2, respectively.

- In comparison, Olmsted *et al.* [10] computed GB energies for a set of 388 Ni and found that the GB energies of general GBs are typically larger than those of special symmetric GBs. Compared to the results of Olmsted *et al.*, the GB energies of the four modeled GB facets, (100)//(926), (110)//(345), (310)//(457), and (310)//($3\bar{1}0$), in our work (1.3~1.4 J/m^2) are in the range of GB energies for general GBs in the literature results.[10]

This comparison justifies that the modeled GBs in our work at least have GB energies that are comparable with the typical values of general GBs.

Supplementary Note 11: Solubilities of S in Ni based FCC phase and alloy compositions

We used isothermal annealing and quenching to preserve high-temperature interfacial structures in the Ni specimens in equilibria with S-enriched secondary phases. Similar thermal treatments in a prior study[11] resulted in sulfur bulk concentrations lower than 100 ppm, which are below the detection limits of EDXS and EELS. Our TEM characterization for all the GBs studied in this work did not observe any precipitates at the GBs.

In this study, the alloy compositions of our specimens should be on the solidus/solvus line at the corresponding final equilibrium temperatures. The solubility limits of sulfur in nickel were estimated to be 20 ppm at 625 °C [11] and 50 ppm at 700 °C [12] in two separate studies. We estimated that the bulk compositions of the Ni-based FCC phase should be within a range of 10-100 ppm. More accurate data on the temperature-dependent solubility limits, which should represent our alloy compositions, are not available.

Supplementary Note 12: An exemplar to verify the thermodynamic equilibrium of Type A (amorphous-like) IGFs

To unequivocally demonstrate that the Type A GB complexions (*i.e.*, amorphous-like IGFs) are indeed in a thermodynamic equilibrium state, Supplementary Fig. 17 shows the character and thickness of the amorphous-like intergranular films (IGFs) at four different Type A facets at different locations in one GB (in a specimen equilibrated and quenched from 675 °C) as an exemplar.

As shown in Supplementary Fig. 17, all four parallel Type A facets of the $(100)//\sim(532)_{\sim 1° \text{ off}}$ orientation exhibited an amorphous-like IGF; furthermore, the measured means (± standard deviations) of the thicknesses of amorphous-like complexions on the four independent facets are 1.12 ± 0.13 nm, 0.98 ± 0.15 nm, 0.76 ± 0.06 nm, and 0.92 ± 0.17 nm, respectively. The narrow distributions of measured thicknesses throughout this long GB suggest the amorphous-like complexion represents a thermodynamic equilibrium state of this GB at this equilibrium temperature.

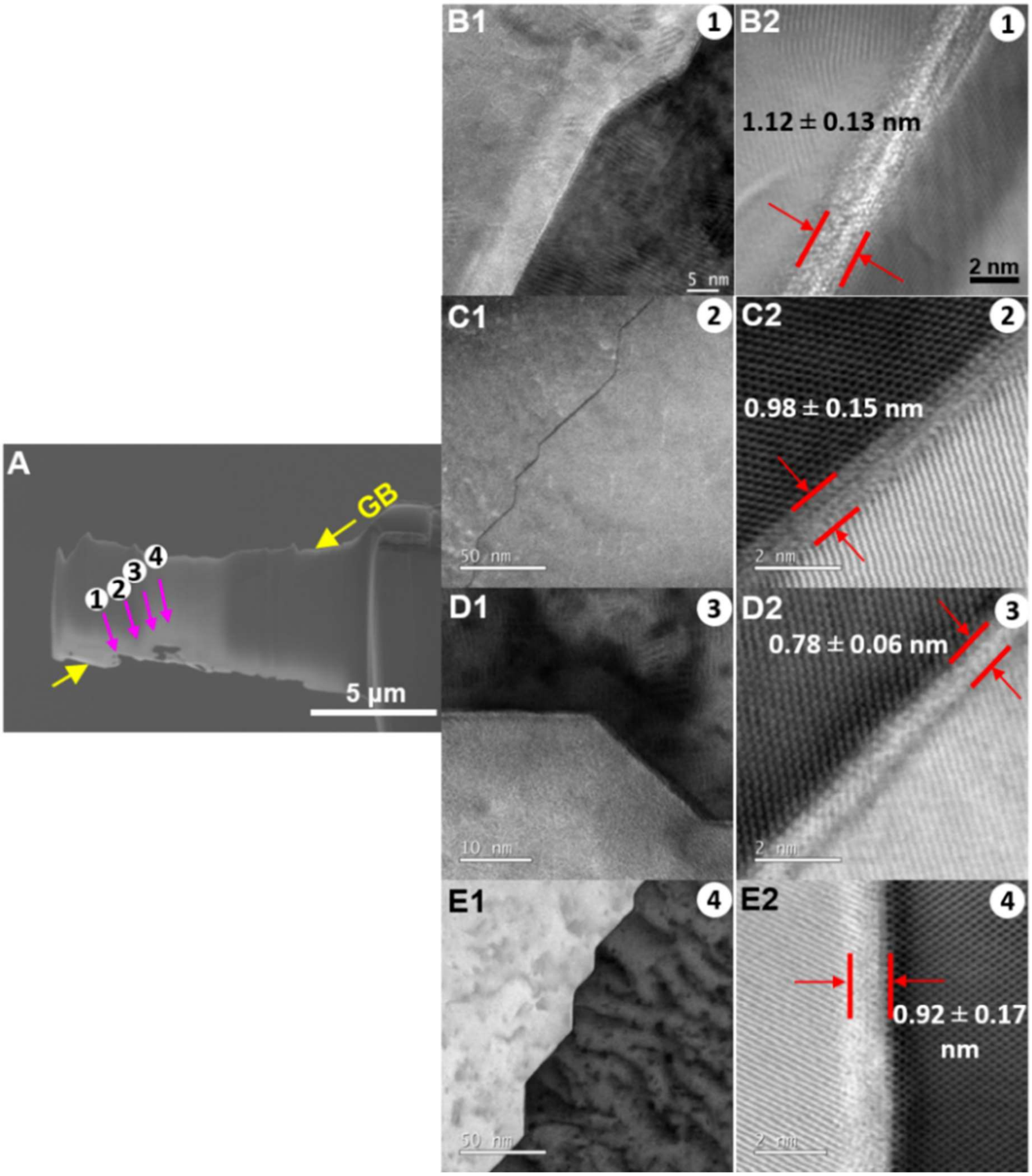

Supplementary Figure 17. TEM images of amorphous-like intergranular films (IGFs) observed at four parallel (but different and disconnected) Type A facets of an identical crystallographic character, (100)//~(532)$_{\sim 1^\circ \text{ off}}$, along the same GB #1 (in a specimen equilibrated and quenched from 675 °C). (A) Image of the TEM specimen made by FIB after low-beam-current thinning and cleaning. The numbers 1-4 denote four different regions that were examined with corresponding images shown in panels (B), (C), (D), and (E), respectively. **(B1, C1, D1, E1)** Low-magnification images showing the GB facets at these four different regions. **(B2, C2, D2, E2)** Corresponding atomic-resolution images showing the Type A GB facets with nanoscale amorphous-like IGFs, where (B2) is an HRTEM image, while (C2), (D2), and (E2) are the STEM ABF images.

Supplementary Note 13: Analyses of partial structural orders in STEM images of Type A (amorphous-like) IGFs

To reveal the partial structural (crystalline) orders in the Type A (amorphous-like) IGFs, we used the "line-by-line FFT" analysis of the (partial) crystalline order (see "Methods") and Periodically-averaged STEM images following the method proposed by Ref. 13. Selected results are shown in Supplementary Fig. 18-20 as well as Fig. 3d in the main article.

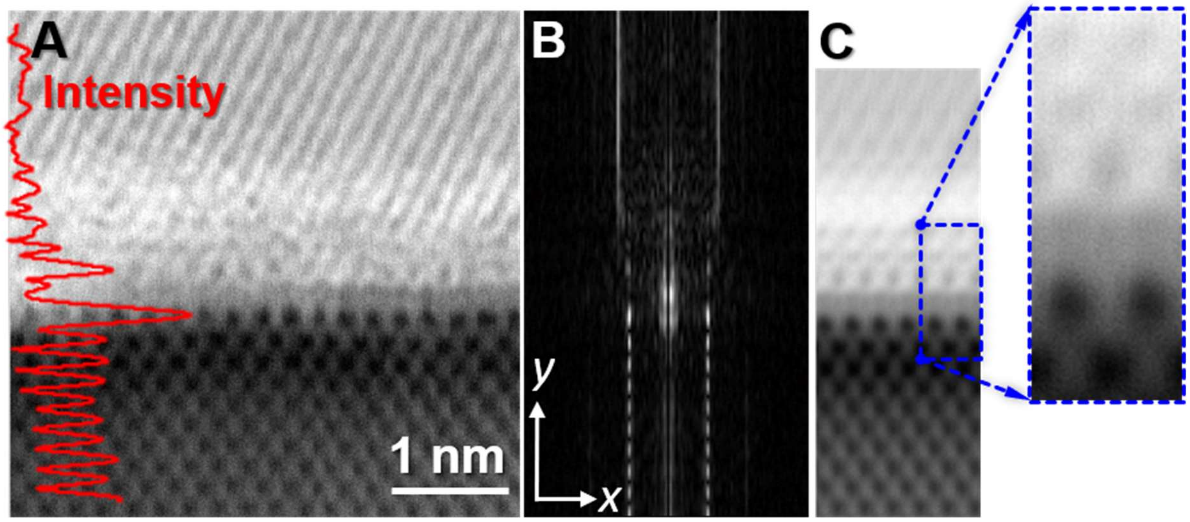

Supplementary Figure 18. Analysis of partial structural order in a Type A (amorphous-like) IGF. (A) Original atomic-resolution STEM ABF image obtained from a sample equilibrated at 675 °C. The red curve is the intensity profile averaged along the horizontal direction, showing the order and periodicity along the vertical direction. **(B)** Line-by-line FFT patterns, showing the partial structural order along the horizontal direction. **(C)** Periodically-averaged STEM ABF image to enhance partial structural orders in the amorphous-like GB core, following the method proposed by Ref. 13.

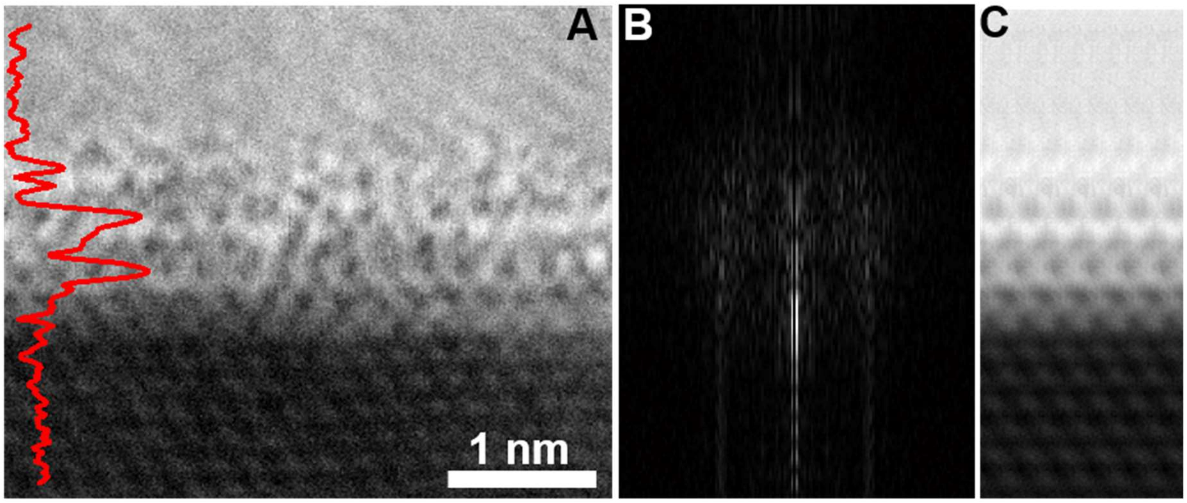

Supplementary Figure 19. Analysis of partial structural order in a Type A amorphous-like IGF – Case #2: (A) Original atomic-resolution STEM ABF image of this Type A GB facet obtained from a sample first annealed at 675 °C and subsequently equilibrated at 575 °C and quenched. The inset is the intensity profile averaged along the horizontal direction, showing the order and periodicity along the vertical direction. **(B)** Line-by-line FFT patterns, showing the order along the horizontal direction. **(C)** Periodically-averaged STEM ABF image to enhance structural details along the horizontal direction, following the method proposed by Ref. 13.

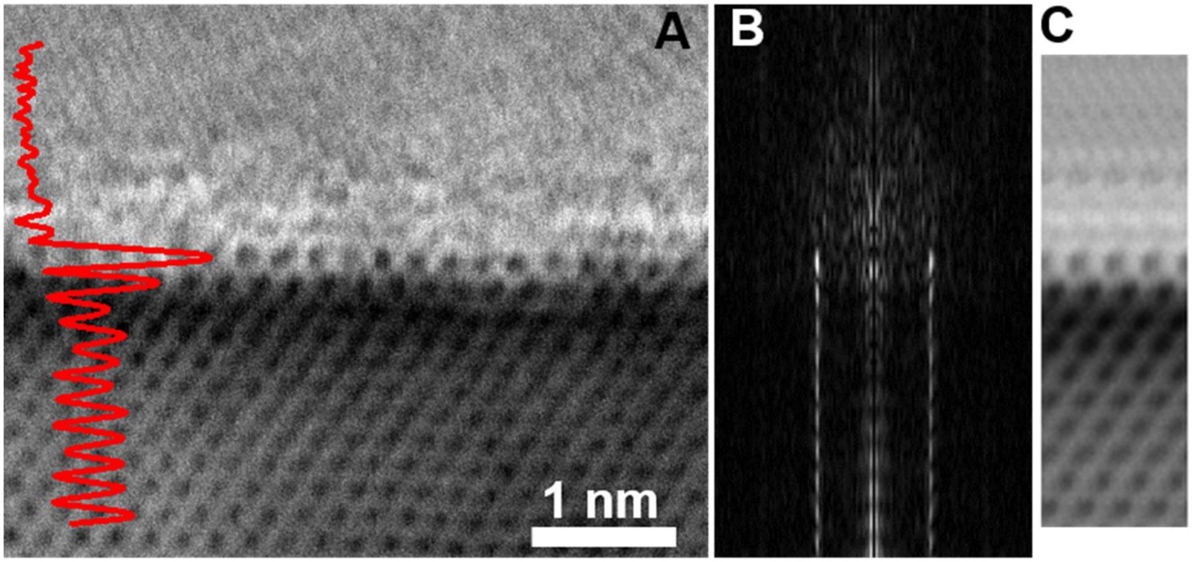

Supplementary Figure 20. Analysis of partial structural order in another Type A amorphous-like IGF – Case #3: (A) Original atomic-resolution STEM ABF image of this Type A GB facet obtained from a sample equilibrated and quenched at 575 °C. The inset is the intensity profile averaged along the horizontal direction, showing the order and periodicity along the vertical direction. **(B)** Line-by-line FFT patterns, showing the order along the horizontal direction. **(C)** Periodically-averaged STEM ABF image to enhance structural details along the horizontal direction, following the method proposed by Ref. 13.

Supplementary Note 14: Intergranular fracture of S-doped Ni specimens

S-doped Ni specimens were subjected to a bending test. As shown in Supplementary Fig. 21, the fracture surfaces of S-doped Ni specimens exhibited characteristic intergranular fracture features. The intergranular fracture occurred through the entire cross-section of the specimens. Faceting was observed on fractured surfaces for all specimens. Denser (and finer) facets were observed in specimens equilibrated (annealed) at higher temperatures.

We used SEM to image all fractured GB planes throughout the entire cross-section to count the fraction of the fractured GB planes without facets and the results are displayed in Supplementary Table 1. Higher fractions of fractured GB planes are faceted in specimens equilibrated (annealed) at higher temperatures. As the equilibration (annealing) temperature decreased, dimples on the fractured GB planes, which are indications of the occurrence of ductile fractures inside some pits of the bulk specimens, were observed in some regions, as shown in Supplementary Figs. 21C and 21D.

As summarized in Supplementary Table 1, higher equilibration (annealing) temperatures result in more brittle fractures (in contrast to normal cases where materials are typically more brittle, which is presumably related to the fact that these specimens were S-saturated Ni specimens, in equilibria with S-rich secondary phases, so that S content is higher at higher equilibrium temperatures) with higher fractions of facets. Some dimples indicated that more ductility was observed in specimens equilibrated (annealed) at lower temperatures.

Supplementary Table 1. Statistical analysis of the fractured surfaces.

Annealing Temperature	Total Number of Fractured Surfaces Examined	Fractured Surfaces without Faceting		Fractured Surfaces with Dimples	
		Number	Percentage	Number	Percentage
675 °C × 5 h	198	31	15.7%	0	0%
675 °C × 5 h + 575 °C ×10 h	397	91	22.9%	0	0%
575 °C × 10 h	188	61	32.4%	5	2.7%
500 °C × 168 h	328	152	46.3%	13	4%

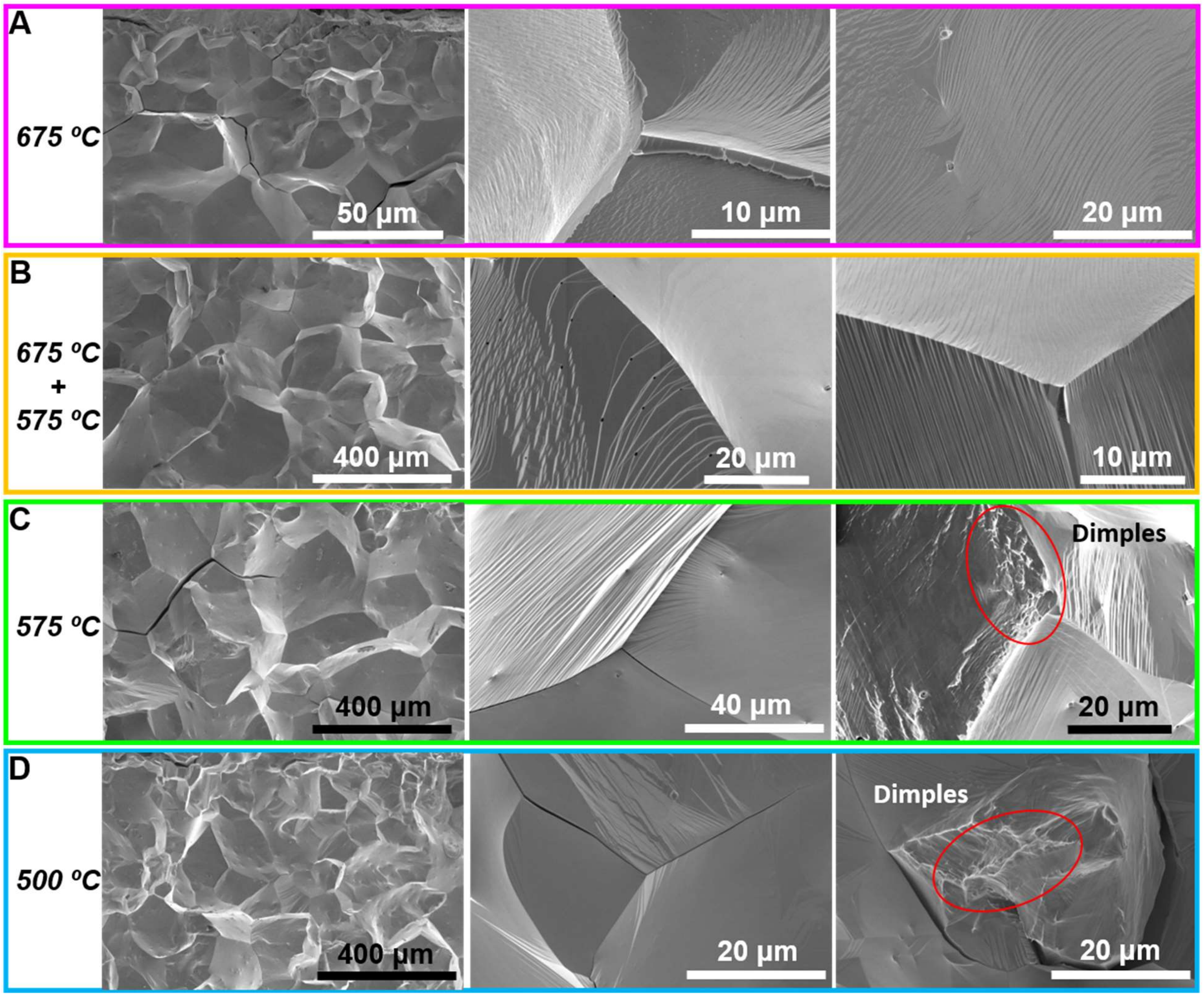

Supplementary Figure 21. Intergranular fractures for S-doped Ni samples annealed at various temperatures. (A) Fractured surfaces of a sample annealed at 675 °C, with characteristic intergranular fracture morphology. The facets on the fractured surfaces (GBs) are evident. **(B)** Fractured surfaces of a sample pre-treated at 675 °C and subsequently annealed at 575 °C, which also exhibit a characteristic intergranular fracture morphology. The majority (but not all) of the fractured surfaces (GBs) are faceted. **(C)** Fractured surfaces of a sample annealed at 575 °C. The majority of fractured surfaces (GBs) exhibit a characteristic intergranular fracture morphology and are faceted, but some fractured surfaces are not faceted; a few dimples were also observed on a small fraction of the fractured surface, indicating some ductile fracture. **(D)** Fractured surfaces of a sample annealed at 575 °C. Most of fractured surfaces (GBs) still exhibit a characteristic intergranular fracture morphology and are faceted, but dimples and non-faceted surfaces were evident on a larger (yet still small) fraction of the fractured surface than those observed from the specimens annealed at higher temperatures. Statistical analyses of the fractured surfaces in these four specimens are shown in Supplementary Table 1.

Supplementary Note 15: Adsorption and excess disorder *vs.* $\Delta\mu$ for Type A *vs.* Type B GB facets

Notably, (100)//(926) and (110)//(345) GB facets exhibited different adsorption and disordering behavior with an increasing chemical potential difference in sulfur ($\Delta\mu_S \equiv \mu_S - \mu_S^{(0)}$, where $\mu_S^{(0)}$ is the chemical potential of sulfur under equilibrium/saturated conditions), as shown in Supplementary Figs. 9A and 9B.

Noting that we often call the modeled GBs as "GB facets," since they correspond to the Type A and Type B facets observed in experiments, even if they are stand-alone GBs in bicrystals with periodic boundary conditions, instead of faceted GBs in simulations.

The adsorption amount of S at the (100)//(926) GB facet is always larger than that at the (110)//(345) GB facet at the same $\Delta\mu_S$, and it increases more rapidly with increasing $\Delta\mu_S$ at (100)//(926) GB facet than that at (110)//(345) GB facet (Supplementary Fig. 9A).

Moreover, GB excesses in disorder for these two GBs are about the same at $\Delta\mu_S < -0.393$ eV. However, at $\Delta\mu_S > -0.393$ eV, the excess in disorder at the (100)//(926) GB facet increases rapidly with increasing $\Delta\mu$, indicating the occurrence of significant interfacial disordering at the (100)//(926) GB facet (Supplementary Fig. 9A), which is consistent with the formation of an amorphous-like complexion (Supplementary Fig. 22B). In contrast, the excess in disorder at the (110)//(345) GB facet only increases moderately with increasing $\Delta\mu_S$ at $\Delta\mu_S > -0.393$ eV (Supplementary Fig. 9B).

It is interesting to point out that a bilayer-like complexion also forms at the (100)//(926) GB facet at a low S chemical potential when $\Delta\mu_S$ is around -0.393 eV (Supplementary Fig. 22A), which transforms to an amorphous-like structure with substantial interfacial disordering when the $\Delta\mu_S$ increases further to $\Delta\mu_S \sim 0$ (Supplementary Fig. 22B). In other words, the amorphous-like GB complexions at high S chemical potentials (corresponding to our experimental conditions, where the specimens were saturated with S) would also likely transform to bilayer-like complexions at lower S chemical potentials, and eventually to "clean" GBs, with reducing S chemical potential (or bulk S content).

In contrast, the bilayer-like complexion at the (110)//(345) GB shows no significant change in structural disordering when $\Delta\mu_S$ increases from -0.501 eV (Supplementary Fig. 23A) to -0.284 eV (Supplementary Fig. 23B).

Our MC/MD simulations suggest that interfacial phase-like transformations at these two facets – from a "clean" GB to bilayer-like complexion in both cases and from a bilayer-like complexion to an amorphous-like complexion at the (100)//(926) GB facet – are likely continuous (instead of first-order), which is indicated by the continuous changes in both adsorption and excess GB disorder in both cases (Supplementary Figs. 9A and 9B).

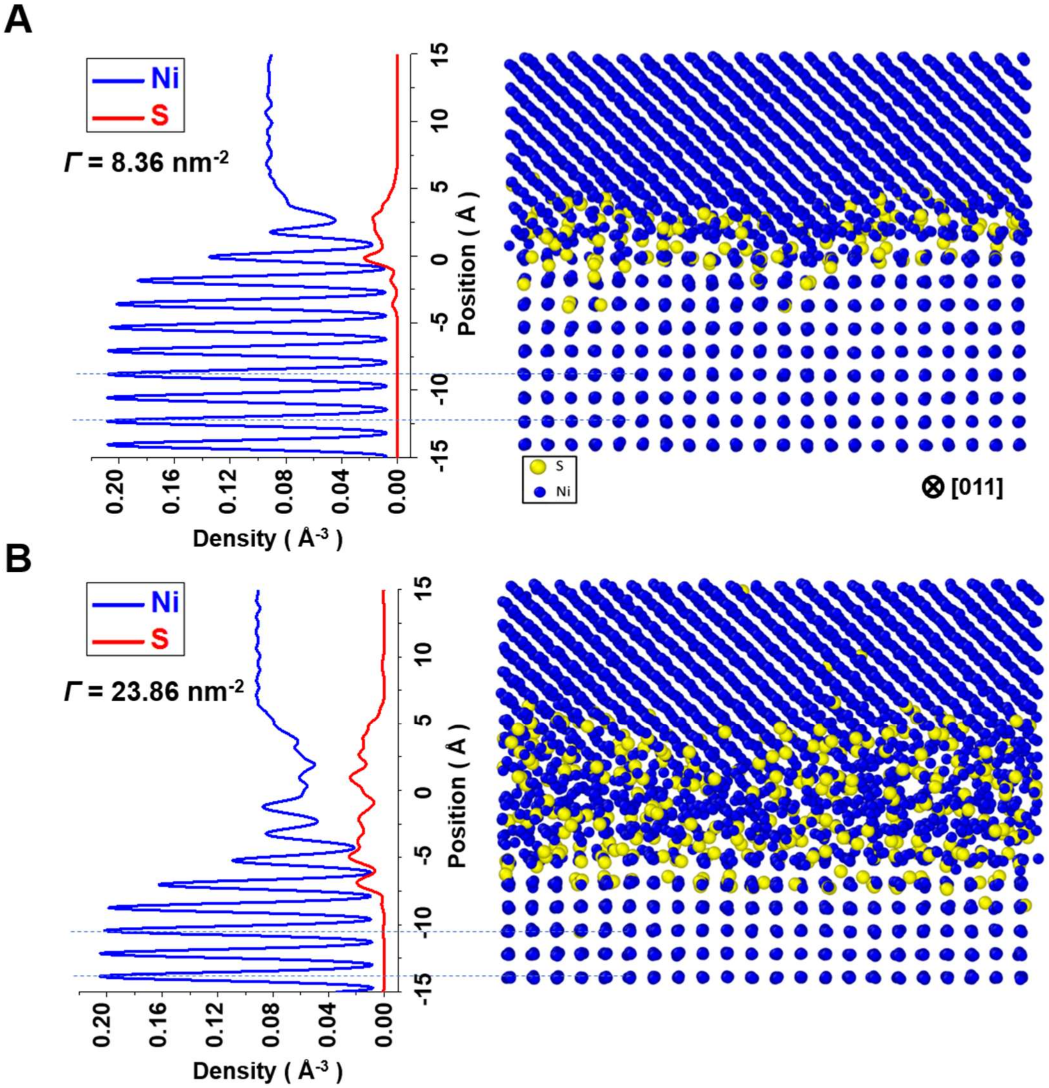

Supplementary Figure 22. Transition from a bilayer-like interfacial complexion to an amorphous-like IGF with increasing GB adsorption of S at the (100)//(926) GB facet from atomistic simulations. This transition is continuous, as shown in Supplementary Fig. 9. The two interfacial structures have a different amount of GB excesses in S: **(A)** 8.36 nm^{-2} (corresponding to the chemical potential difference of sulfur $\Delta\mu_S = -0.393$ eV) and **(B)** 23.86 nm^{-2} ($\Delta\mu_S = 0$ eV). The density profiles of Ni and S from simulated atomic structures are shown on the left.

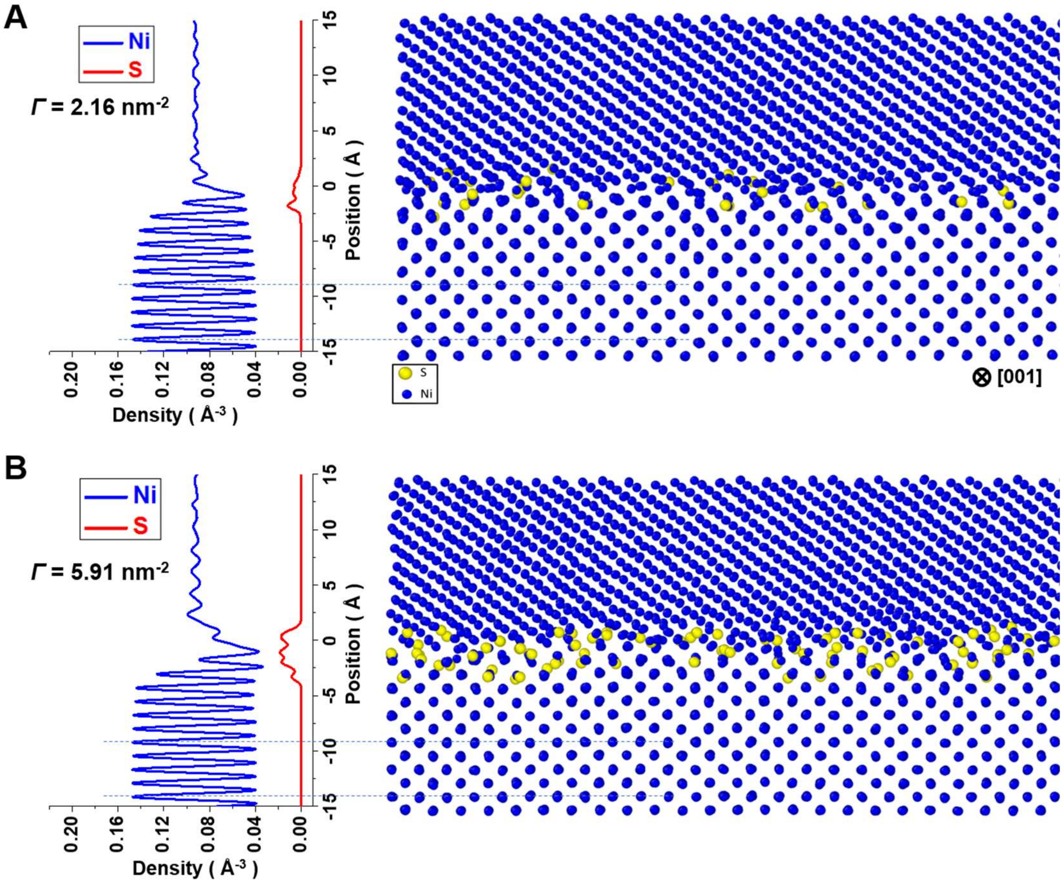

Supplementary Figure 23. Formation of a bilayer-like interfacial structure with increasing GB adsorption of S at the (110)//(345) GB facet from atomistic simulations. The two interfacial structures correspond to a different amount of GB excess in S: **(A)** 2.16 nm^{-2} (corresponding to the chemical potential difference of sulfur $\Delta\mu_S = -0.501$ eV) and **(B)** 5.91 nm^{-2} ($\Delta\mu_S = -0.284$ eV). The density profiles of Ni and S from simulated atomic structures are shown on the left.

Supplementary Note 16: Bond analysis, polar Ni-S structures, and bipolar interfacial structures

A Ni-S bond is considered to form between a S atom and a Ni atom if the distance between them is smaller than a certain cutoff. This cutoff is selected as 2.4 Å, which is larger than the bulk compound bond lengths of 2.05, 2.28, 2.38, and 2.34 Å for α-S, Ni_3S_2, NiS and NiS_2, respectively.

It was observed that S atoms do not in general sit on the lattice positions of Ni in the GBs.

It was found that a large portion of Ni-S structures are polar, where Ni atoms are concentrated at one side of the S atom. Two typical polar Ni-S structures are shown in Supplementary Fig. 6. The polar Ni-S structures exist in both bilayer-like and amorphous-like GBs. The distributions of Ni-S bond angles and bond lengths are similar, as shown in Supplementary Fig. 24(A)-(D).

Furthermore, as shown in Supplementary Fig. 24, the polar Ni-S structures observed in S-doped Ni GBs are different from all known Ni-S compounds, such as those in Ni_3S_2, NiS, NiS_2, and Ni_3S_4. Although the bond angles (*i.e.*, 60°~70° and around 120°) and bond lengths (*i.e.*, around 2.25Å and 2.35Å) found in GBs correspond to the peaks in compounds, their distributions are different.

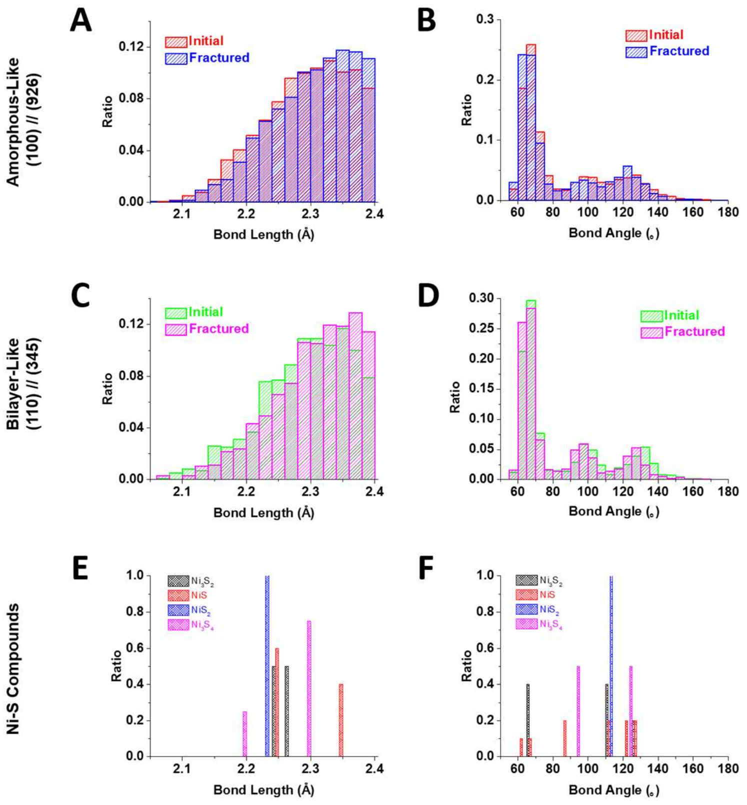

Supplementary Figure 24. Comparison of distributions of Ni-S bond lengths and Ni-S-Ni bond angles in a Type A (100)//(926) GB facet and a Type B (110)//(345) GB facet (from the hybrid MC/MD simulations) with those in known Ni-S compounds: Ni_3S_2, NiS, Ni_3S_2, and Ni_3S_4 (from the Materials Project database). Distributions of Ni-S bond lengths and Ni-S-Ni bond angles in the initial and fractured specimen from the tensile simulation for **(A, B)** Type A (100)//(926) and **(C, D)** for Type B (110)//(345) GB facets, as well as for **(E, F)** of known Ni-S compounds.

Supplementary Note 17: Tensile properties from MD testing

MD simulations of tensile testing were conducted on the GB structures obtained at different chemical potential differences for both the (100)//(926) GB facet (Supplementary Fig. 25) and the (110)//(345) GB facet (Supplementary Fig. 26). A constant strain rate was applied by deforming the simulation cell during the MD simulations. The periodic boundary condition was used in the direction perpendicular to the grain boundary. Due to the periodic boundary condition, there were two GBs in each simulation cell. Due to the constraint of computational time and resource, the strain rate in MD simulations is typically much higher than those used in experiments, which is an intrinsic limitation of all MD simulations. The same strain rate of 10^9 s^{-1} was applied to the pure and S-doped Ni models for a valid comparison.

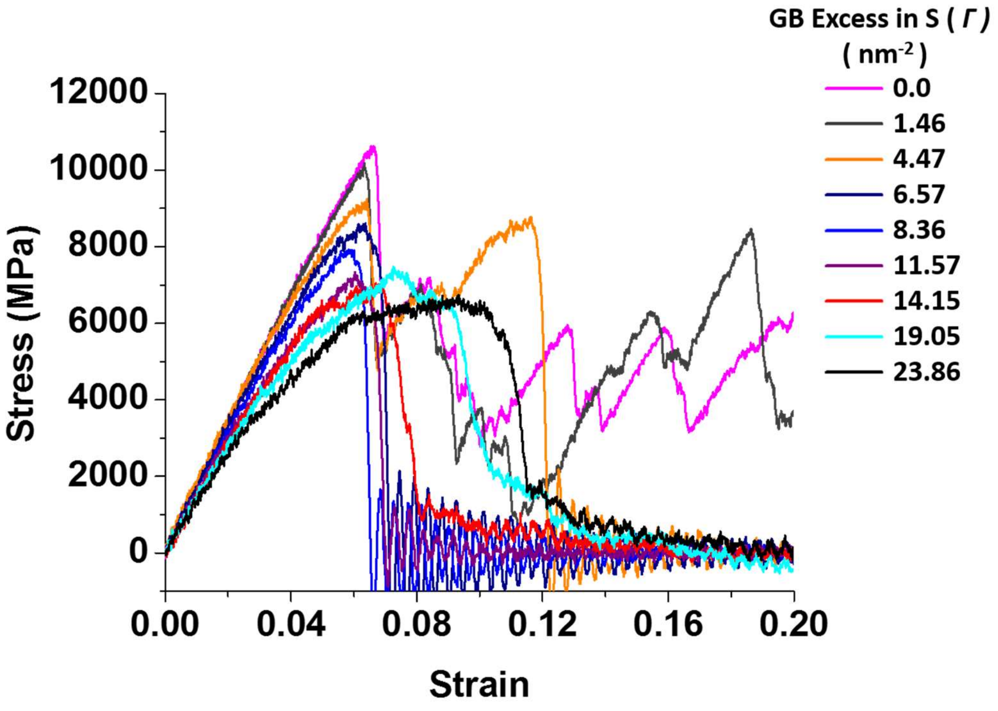

Supplementary Figure 25. Stress-strain curves for tensile simulation of a Type A (100)//(926) GB facet. The different curves are simulated results for GBs with different amounts of GB excesses of S (Γ's) labeled in the graph. The GB is brittle when $\Gamma \geq 6.57$ nm^{-2}.

It is clearly shown that these GBs undergo a ductile-to-brittle transition as the GB excess in S increases. For the (110)//(345) GB facet (Supplementary Fig. 26), this transition occurred when the GB excess in S reached 4.33 nm^{-2}. The toughness of these GB models was obtained by computing the area under the stress-strain curves for an applied strain of up to 0.2 (Supplementary Figs. 9C and 9D). When the chemical potential difference of sulfur $\Delta\mu_S$ reaches the value of ~−0.5 eV, the toughness of both (110)//(345) and (100)//(926) GB facets decreases rapidly, indicating ductile-to-brittle transitions.

Despite the high strain rate, dislocations and strain-induced phase transformation initiated in the pure Ni, as shown in Supplementary Figs. 7B, 7E and 7H. In contrast, no dislocation activity was observed in S-doped Ni and the intergranular fractures were completely brittle, as shown in

Supplementary Figs. 7C, 7F and 7I. This clearly indicated that S caused the intergranular brittle fracture in Ni.

As shown in Supplementary Figs. 9C and 9D, there are strong correlations between the computed tensile toughness values (*i.e.*, the areas beneath the computed stress-strain curves; see the definition and discussion subsequently on P. 41-42) from MD simulations and the bipolar index (β) values calculated from the atomistic interfacial structures (see "Methods") in both cases, indicating that the formation of bipolar interfacial structures is the root cause of the embrittlement.

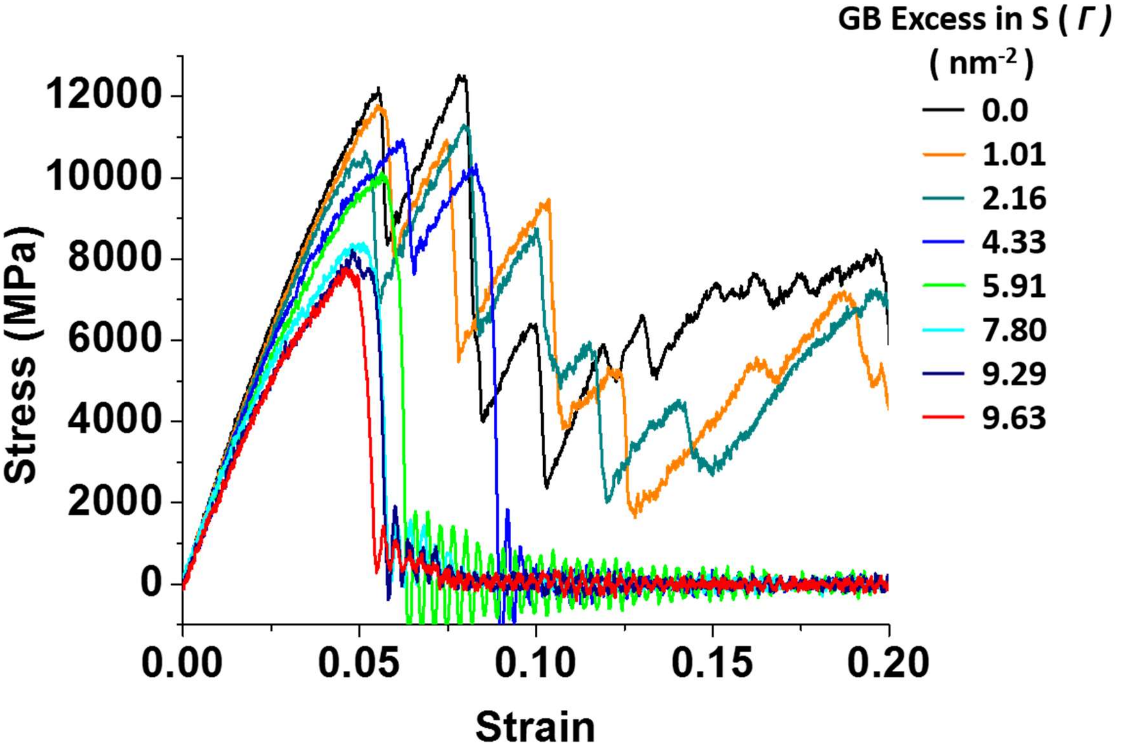

Supplementary Figure 26. Stress-strain curves for tensile simulation of a Type B (110)//(345) GB facet. The different curves are simulated results for GBs with different amounts of GB excesses of S (Γ's) labeled in the graph. The GB is brittle when $\Gamma \geq 4.33$ nm^{-2}, which roughly corresponds to the condition of forming a bilayer-like GB complexion.

Supplementary Note 18: Further discussion of GB embrittlement and computed tensile toughness

GB embrittlement is defined as segregation-induced intergranular brittleness of normally ductile polycrystals following Rice and Wang [14] and others. The GB embrittlement is correlated with (but not identical to) the ideal work of interfacial separation ($\mathbf{2\gamma_{int}}$ in the Rice-Wang model,[14] as discussed below), which can be assessed by theories and DFT calculations. Experimentally, prior studies assessed the GB embrittlement from counting the fraction of intergranular fractures[15,16] or examining the effect of the embrittling element segregation in changing the ductile-to-brittle transition temperature (DBTT)[17]. Yamaguchi and Kameda also showed that the GB embrittlement can be induced by a small amount of solute segregation, if the embrittlement is defined as the reduction of local fracture stress in notched-bar bending tests or fracture toughness in compact tension tests.[18]

In this study, we used a "computed tensile toughness" from MD simulations, which was determined by integrating the stress-strain curve:

$$\text{(Computed) Tensile Toughness} \equiv \int_0^{\varepsilon_f} \sigma \, d\varepsilon \tag{4}$$

where ε is strain, ε_f is the strain upon failure, and σ is the stress. This computed tensile toughness is a fracture stress defined in computation (the MD simulation), which is **not** the fracture toughness. This computed tensile toughness measures the ability of the testing material to absorb energy before fracturing in the MD simulation, including the energies for intragranular defects formation and movement (including dislocations, if they do initiate as in the case of pure Ni) and cracking (the formation of crack tips and subsequent decohesion).

We again emphasize that this computed tensile toughness (that is the energy of mechanical deformation per unit volume prior to fracture in the MD simulation, with a unit of J/m^2 or Pa) is not the "fracture toughness" defined in fracture mechanics (that has a unit of $Pa\sqrt{m}$). The fracture toughness measures a material's ability resist fracture and it is a materials property (independent of the length and sharpness of the existing crack); however, it is difficult to compute the fracture toughness directly for the current case.

In general, the experimentally-measured tensile toughness is not an intrinsic material property for a brittle material since it depends on the size and shape of any preexisting cracks and defects; however, this "computed tensile toughness" can be well defined in computation (for a crack-free material) and conveniently calculated from the MD simulation to represent (at least) the (relative) extent of embrittlement of the testing material.

In a realistic situation, the fracture stress (or an experimentally-measured tensile toughness of a real material) depends on the length and sharpness of the existing crack(s). However, the current simulations do not include preexisting cracks (so that the computed tensile toughness measures the intrinsic material property, including the nucleation of any cracks). More importantly, this computed tensile toughness can be conveniently computed from the MD tensile simulation for a fair relative comparison of pure and doped specimens to (at least qualitatively) assess the GB embrittlement.

The tensile toughness should be correlated with (but not identical to) the work of separation:

$$\int_0^{\varepsilon_f} \sigma \, d\varepsilon \sim \text{the work of separation} \equiv \int_0^{+\infty} \sigma \, d\varepsilon \qquad (5)$$

For a (completely) brittle intergranular fracture, **the <u>ideal</u> work of interfacial separation** (**$2\gamma_{int}$**, as denoted by Rice and Wang[14]) without any energy dissipation from plastic deformation or other defects generation, also known as *the work of adhesion* in the wetting community, can be expressed for a GB as:

$$2\gamma_{int} = 2\gamma_S - \gamma_{GB}, \qquad (6)$$

where γ_S is the surface energy (after the fracture) and γ_{GB} is the GB energy (before the fracture). This <u>ideal</u> work of interfacial separation ($2\gamma_{int}$) is used to characterize the extent of GB embrittlement in the Rice-Wang model[14] and many DFT calculations.

We should further note that MD simulations can also provide further insights of failure modes, *e.g.*, dislocations did initiate in pure Ni with ductile fractures, whereas the brittle intergranular fractures occurred in S-doped Ni, as shown in Supplementary Fig. 7.

Supplementary Note 19: Kikuchi patterns and measured GB disorientation angles

The misorientation of a GB is the rotation required to bring one set of crystal axis (of one of the two abutting grains) into coincidence with the other[19]. One can define a "disorientation angle" as the (smallest) rotation angle to bring two sets of crystal axes (of the two abutting grains) into coincidence within the fundamental zone for cubic symmetries, which should be in the range of 0° to ~62.8° due to the cubic symmetry.

The crystal axes of the adjacent grains were determined from Kikuchi patterns, which were obtained via Ronchigrams in STEM. The GBs were set to the "edge-on" condition, in which the electron beam is parallel to the GB planes. The two abutting grains were set on the same tilting angle. Tilting was eliminated during the Kikuchi patterns acquisition. Supplementary Table 2 listed the disorientation angles of selected GBs determined from Kikuchi patterns.

For example, using the procedure given in Ref. 20, the rotation angle/axis representation for two abutting grains of GB #1A was determined to be 70.59° / $[\overline{0.83}\ 0.37\ 1.47]$ with respect to the left grain. Considering all 24 identical rotation operations given the cubic symmetry, the minimum rotation angle within the fundamental zone (*i.e.*, the disorientation angle of GB #1A) is ~34.4° with approximate rotation axis of $[1.17\ \overline{0.24}\ 0.4]$.

The results (shown in Supplementary Table 2) verified that the misorientation angles of these randomly-selected GBs are not small-angle GBs. Examples of Kikuchi patterns are displayed in Supplementary Fig. 27.

Supplementary Table 2. Disorientation angles of selected GB facets determined by Kikuchi patterns. Noting that GB#1A and GB#2B are two facets of the same GB with identical misorientation (within the measurement errors).

GB Facet	Orientation Relationship	Disorientation Angle (Determined from Kikuchi Patterns)
#1A	(100)//~(532)$_{\sim 1^\circ\ \mathrm{off}}$	~34.4°
#1B	(310)//~(744)$_{\sim 1^\circ\ \mathrm{off}}$	~35.5°
#5B	(110)//~(331)$_{\sim 2^\circ\ \mathrm{off}}$	~49.4°

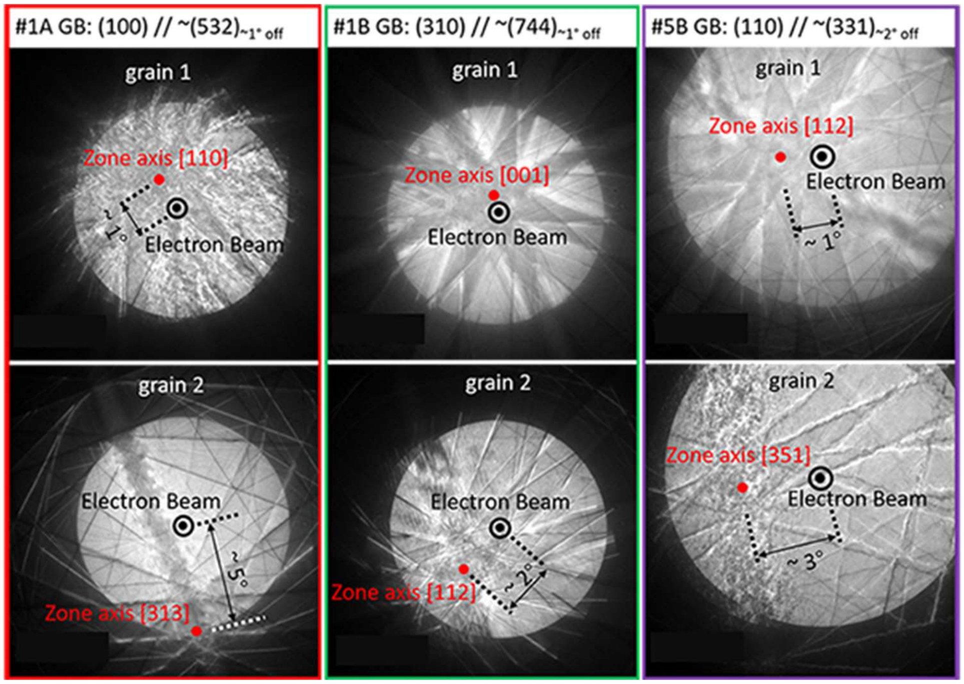

Supplementary Figure 27. Kikuchi patterns obtained from the abutting grains for the three representative GB facets examined in this study. The specific orientation relationships for these three GBs are: GB #1A: (100)//~(532)$_{\sim 1^\circ \text{ off}}$, GB #1B: (310)//~(744)$_{\sim 1^\circ \text{ off}}$, and GB #5B: (110)//~(331)$_{\sim 2^\circ \text{ off}}$. The red dots on the Kikuchi patterns indicate the zone axis of the crystals, while the black dots in circles indicate the position of the electron beam. The tilting angle between the zone axis and the beam center is estimated and indicated in the figures.

Supplementary Note 20: Three types of GBs: critical comparisons of simulations and experiments

The assembly of simulated results of five GB facets (Supplementary Table 3) and experimental observations of 29 independent GB facets (Table 1 in the main article) clearly suggest that the formation of Type A *vs.* Type B facet is dictated by the orientation of the lower-index grain terminal plane, instead of misorientation as commonly believed.

On one hand, we have observed the following (Table 1 in the main article) in the experiments:

(A) 18 independent GB facets have one low-index terminating grain surface of the (100) plane with another high-index matching grain surface; all 18 facets exhibit Type A (amorphous-like) complexions; selected STEM images are shown in Supplementary Fig. 29.

(B) 11 GB facets have one low-index grain surface of the (310), (311), (211), or (110) plane with another high-index grain surface; all exhibit Type B (bilayer-like) complexions; selected STEM images are shown in Supplementary Fig. 30.

On the other hand, simulations of multiple GB facets fully agree with experimental observations and further support the idea that the orientation of the lower-index grain terminal plane, instead of the misorientation, dictates the interfacial structures, where Type A (amorphous-like) complexions form on (100) facets and Type B (bilayer-like) complexions form on all other facets except for low-energy twin, low-angle, and presumably some other special GBs (Supplementary Table 3). In addition to the types of complexions formed, the simulated GB adsorption amounts of S also agree with experimental observations (the computed *vs.* measured Γ's, when comparisons are made for GB facets with the same dictating grain terminal planes, *e.g.*, (100), (110), and (310) as shown in Supplementary Table 3).

As a particular interesting case, both Σ11 (310)//(3$\bar{1}$0) and Σ3 (310)//(457) facets exhibit Type B (bilayer-like) complexions with similar computed *vs.* measured Γ's (12.2 *vs.* 12.1 S atoms per nm^2), which agree very well with the experimental observation of the Type B complexion at (310)//~(744) with measured Γ = 12.6 S atoms per nm^2; see further comparisons in Supplementary Figs. 42 and 43. This strongly implies that interfacial structure and segregation are dictated by the orientation of the lower-index grain terminal plane, *i.e.* (310) in this specific case, instead of by the misorientation (Σ11 *vs.* Σ3).

Here, it is worth noting that the asymmetric Σ3 (310)//(457) GB facet behaves more like a general GB (despite being a Σ3 GB) with a GB energy that is significantly higher than that of the special Σ3 (111)//(111) twin boundary, but more comparable with other more general GBs (Supplementary Table 3).

Furthermore, five normally "clean" or Type C GBs were found (Table 1 in the main article; Supplementary Figs. 39 and 40). Four of the five Type C GBs observed are twin boundaries (Supplementary Table 1 and Fig. 39) and the other is a low-angle GB (Supplementary Fig. 40), and all are low-energy GBs with little driving force for S adsorption. The absence of S adsorption on these GBs is due to the small free volume and low fraction of "broken bonds" at these low-energy GBs, which is a well understood and established phenomenon; *e.g.*, a similar phenomenon is observed in the Ni-Bi system.[21] Our simulation (Supplementary Fig. 44) further supports that the Σ3 (111)//(111) twin boundary is normally "clean" or Type C; although we initially set the GB

dividing plane of this Σ3 to be $(100)//(2\bar{1}2)$, nanoscale faceting occurred, producing "clean" (111)//(111) symmetric twin boundary facets (Supplementary Fig. 44).

Supplementary Table 3. Summary of the complexion formation of simulated GBs of different grain terminating plane orientations. The results clearly suggest the formation of complexions correlated with the orientation of the lower-index grain terminating plane, instead of the misorientation. The simulation results agree well with experimental observations and our hypothesis. The GB energies (γ_{GB}'s) listed in the following table were calculated for pure Ni using an EAM potential that was used by Olmsted *et al.*,[10] who calculated the γ_{GB}'s for general GBs to be in the range of 1-1.5 J/m^2; these results suggest that most of these asymmetric GBs behave more like general GBs with comparable γ_{GB}'s, except for Σ3 (111)//(111) twin boundary, which has a substantially lower γ_{GB}.

GB Facet Simulated		γ_{GB} for Pure Ni (J/m^2)	Complexion Formed	Computed Γ (nm^{-2})	Simulated Results Shown in	Matching Experiments	Measured Γ (nm^{-2})
Σ11	(100)//(926)	1.358	Type A (Amorphous-like)	23.9	Fig. 3 (Main Article)	(100)//~(532)<br>(100)//~(7 11 11)<br>(100)//~(211)<br>(100)//~(320)	15-35 (mean = ~25)
	$(310)//(3\bar{1}0)$	1.308	Type B (Bilayer-like)	12.2	Supplementary Fig. 43	(310)//~(744)	12.6
Σ15	(110)//(345)	1.314	Type B (Bilayer-like)	6.98	Fig. 4 (Main Article)	(110)//~(331)	9.63
Σ3	(310)//(457)	1.345	Type B (Bilayer-like)	12.1	Supplementary Fig. 42	(310)//~(744)	12.6
	Average: $(100)//(2\bar{1}2)$ → Faceting/Rotating to → Type C (111)//(111) Twin Facets	0.00718 (Twin)	Type C ("Clean")	~0	Supplementary Fig. 44	(111)//(111)	~0
		N/A	S Clusters	N/A		N/A	N/A

Supplementary Note 21: Beyond embrittlement: abnormal grain growth in Ni-S

Beyond the GB embrittlement, the current study also provided a significant new insight about the origin of abnormal grain growth in S-doped Ni (including electrodeposited nanocrystalline Ni with S contamination).[22-24]

On one hand, a series of prior studies have demonstrated that the abnormal grains in S-doped Ni are often cubic with (001) terminal surface planes.[22-24]

On the other hand, this current study showed that GB facets with one lower index terminal grain surface of the (100) plane and another high-index matching surface all exhibit Type A amorphous-like IGFs that are more disordered with higher levels of S absorption (than all other GBs examined); in contrast, all other types of GB facets (at least all those that have been examined in this study) are less disordered with lower levels of S adsorption (being Type B bilayer-like or Type C "clean" GBs). See, *e.g.*, Table 1 in the main text and Supplementary Notes 1, 2, and 20 for summaries of relevant experimental observations and modeling results.

Combining the two above observations, we propose/conclude that abnormal grain growth in S-doped Ni, including that in electrodeposited nanocrystalline Ni with S contamination, [22-24] is likely a result of enhanced GB mobility of the more disordered Type A amorphous-like GBs, in comparison with other type of more ordered GBs.

Here, our proposed mechanism is consistent with Dillon *et al.*'s theory of abnormal grain growth mechanisms for doped Al_2O_3 and other systems;[3,25,26] they observed that the abnormal grain boundaries exhibit complexions with higher levels of impurity adsorption and structural disorder than the "normal" boundaries in the same specimens, thereby attributing abnormal grain growth to complexion transitions.[3,25,26] Yet, there may be a difference. The abnormal grain growth in S-doped Ni can (in principle) be resulted from the highly-anisotropic nature of complexion formation in this system (and may depend less on the occurrence of a complexion transition).

Supplementary Note 22: Summary of TEM/STEM observations

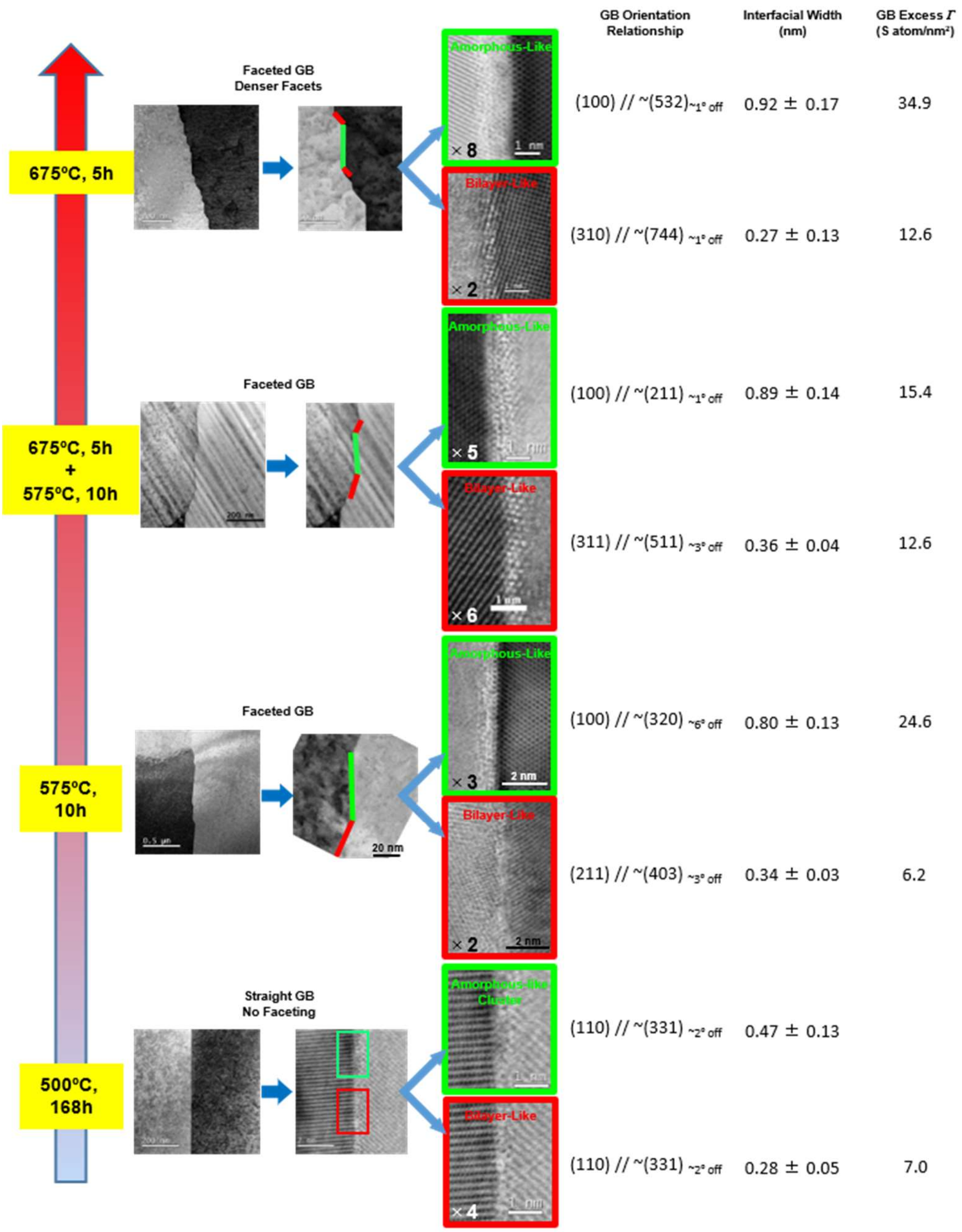

Supplementary Figure 28. Schematic illustration of observed Type A and Type B facets (complexions) formed at different equilibration conditions, along with the orientation relationship, measured interfacial width, and measured GB excess of S (Γ) for each case. Numbers at the bottom-left corner in the STEM ABF images (*e.g.*, “X8” in the first image) indicate that the number of facets (or well-separated locations in one case of a long flat GB) on the same GB that have been examined, all of which show a similar character.

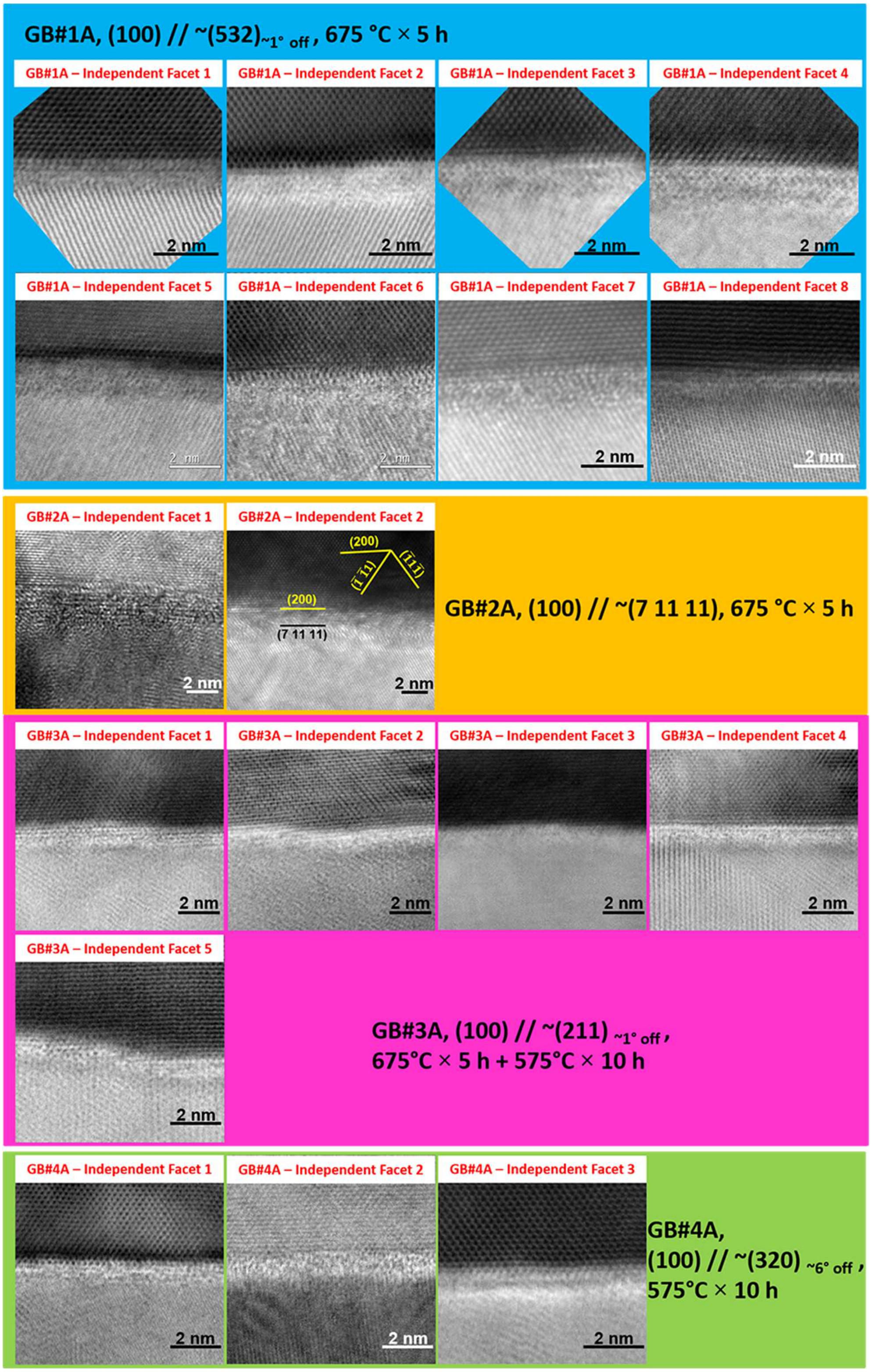

Supplementary Figure 29. Summary of Type A (amorphous-like) complexions observed at 18 independent facets at four different GBs in specimens annealed at three different conditions.

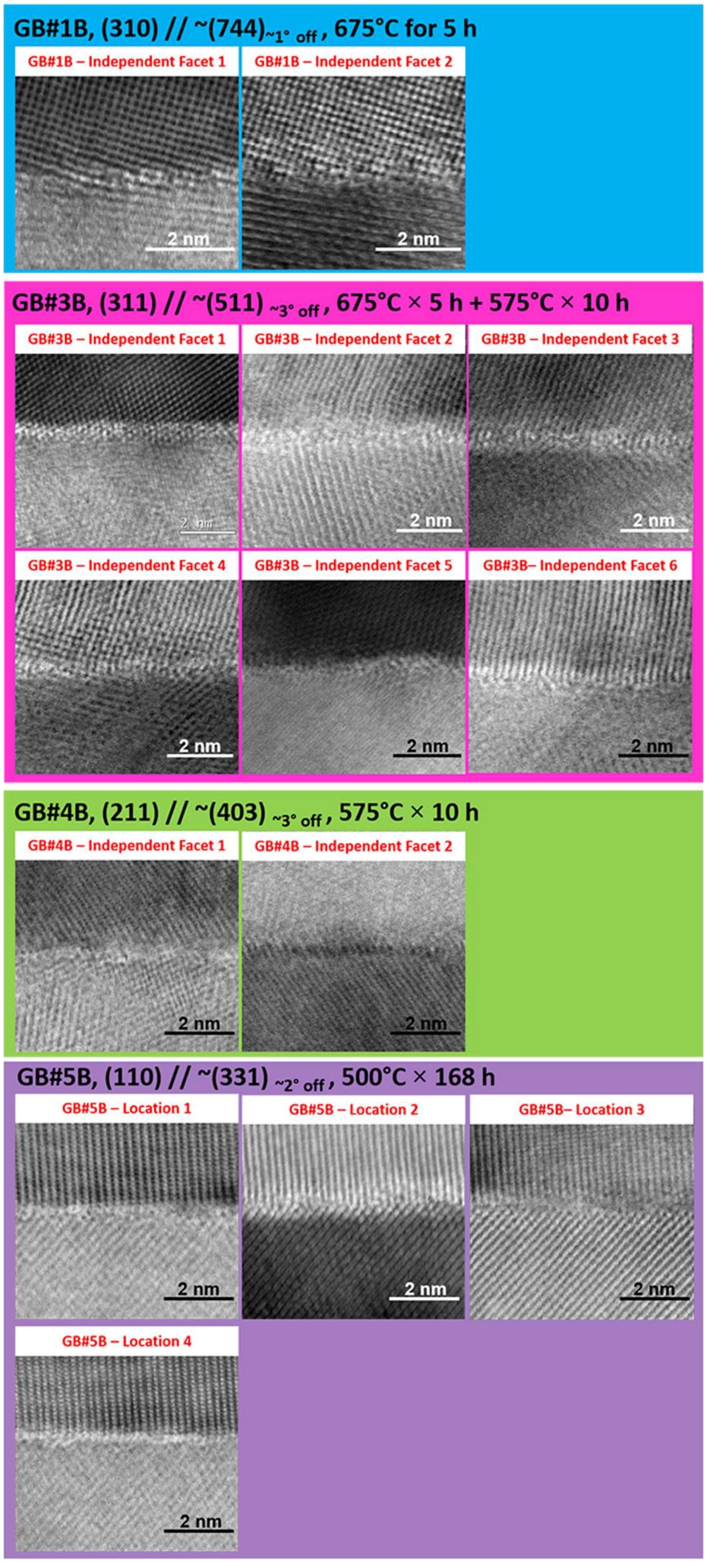

Supplementary Figure 30. **Summary of Type B (bilayer-like) complexions observed at 11 independent facets/locations at four different GBs in specimens annealed at three different conditions.**

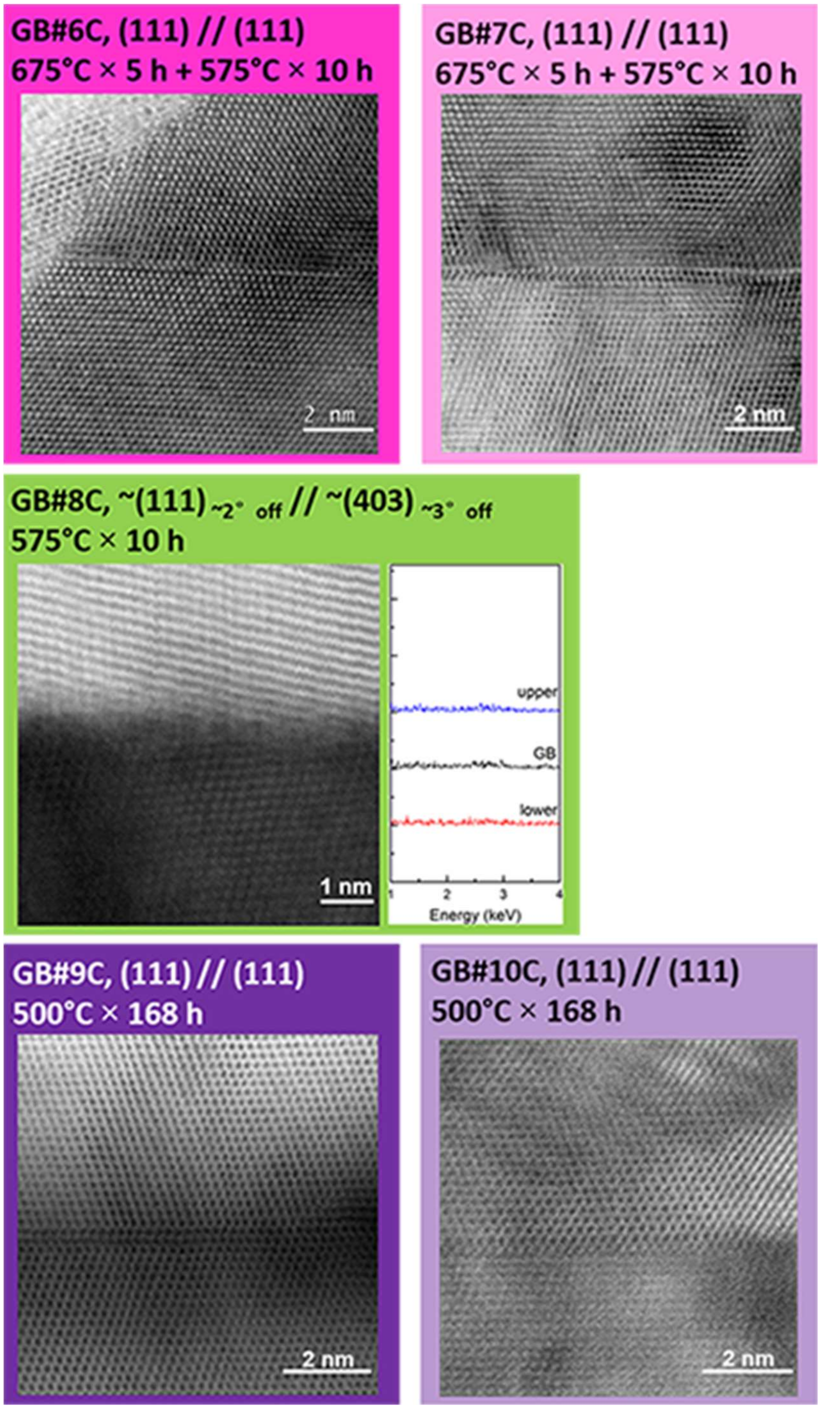

Supplementary Figure 31. Summary of Type C ("clean") complexions observed at four symmetric Σ3 (111)//(111) twin boundaries and one low-angle GB, all with low GB energies and low thermodynamic driving forces for S segregation.

Supplementary Note 23: Additional TEM/STEM images of individual observations: a case-by-case list

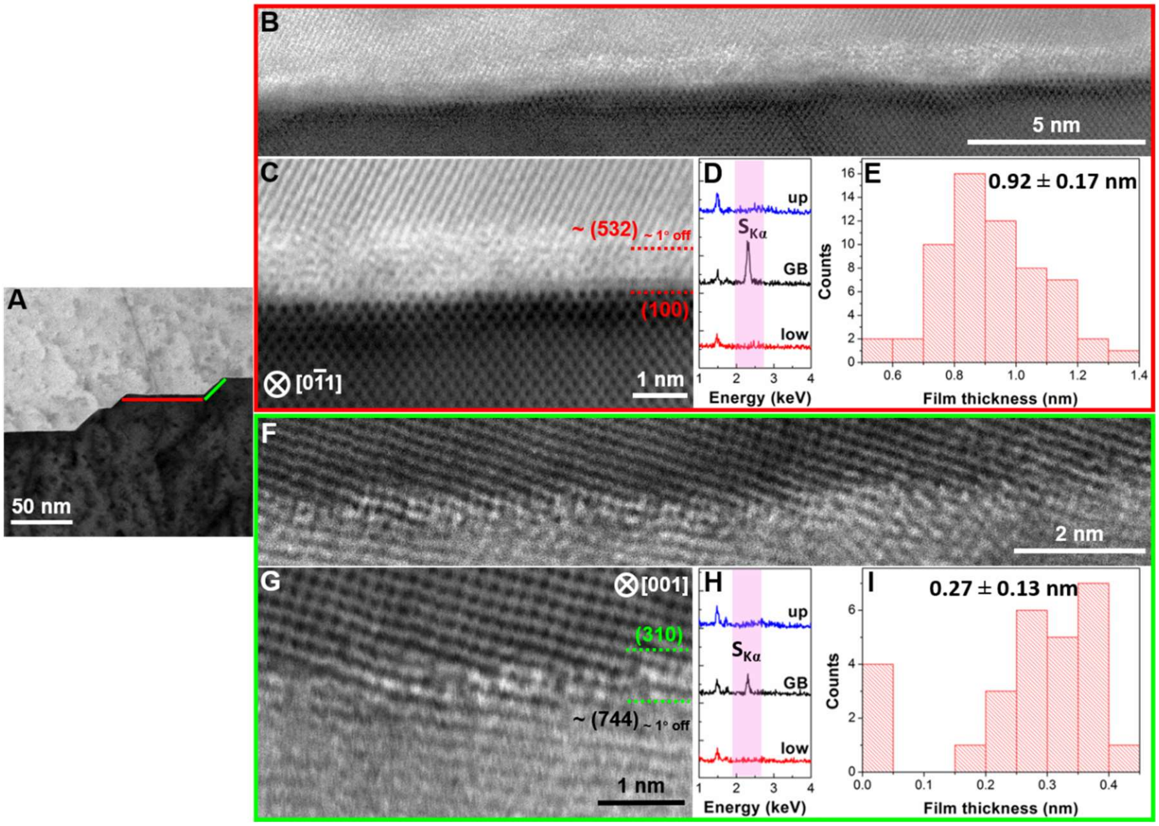

Supplementary Figure 32. (GB #1A and #1B) Type A and Type B facets coexisted at the same GB #1. This specific GB was randomly selected from a S-doped Ni specimen quenched from 675 °C. **(A)** Low-magnification STEM image showing the GB with two different types of facets. **(B)** Intermediate-magnification STEM ABF image showing a Type A facet highlighted by the red line in panel (A). **(C)** STEM ABF image of the Type A GB facet at a higher magnification; the orientation relationship was determined to be $(100)//\sim(532)_{\sim 1^\circ \text{ off}}$. **(D)** The corresponding EDX spectra are obtained from the GB and two adjacent crystals, respectively, which indicate that this Type A GB facet is S-enriched. **(E)** The histogram of the measured thicknesses of the amorphous-like intergranular films formed at parallel (different) Type A facets; *60 independent values of thickness* were measured from *seven different facets* (HRTEM image is excluded). The spacing between two adjacent measured points on the same facet is 1 nm to avoid bias selection; the mean thickness was measured to be 0.92 nm and the corresponding standard deviation is 0.17 nm. **(F)** Intermediate- and **(G)** high-magnification STEM ABF images of a Type B GB facet highlighted by the green line in (a); the orientation relationship is $(310)//\sim(744)_{\sim 4^\circ \text{ off}}$. **(H)** The corresponding EDX spectra obtained from the GB and two adjacent crystals indicate that this Type B GB facet is also S-enrich. **(I)** The histogram of the measured effective interfacial thicknesses of this Type B GB facet formed at parallel (different) facets of the same GB.

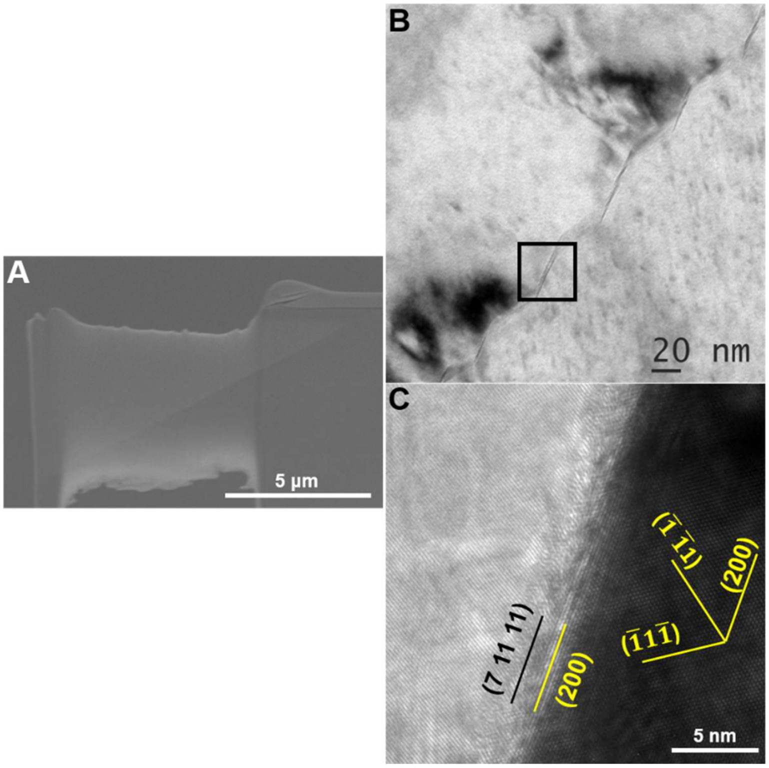

Supplementary Figure 33. (GB#2A) Another example of a Type A GB facet observed in another FIB/TEM specimen in a sample quenched from 675 °C. (A) Image of the TEM specimen made by FIB after low-beam-current thinning and cleaning, where the GB is visible. **(B)** Low-magnification TEM images showing the faceted GB. **(C)** High-magnification HRTEM image showing the disordered interface phase. The orientation relationship is (100)//~(7 11 11).

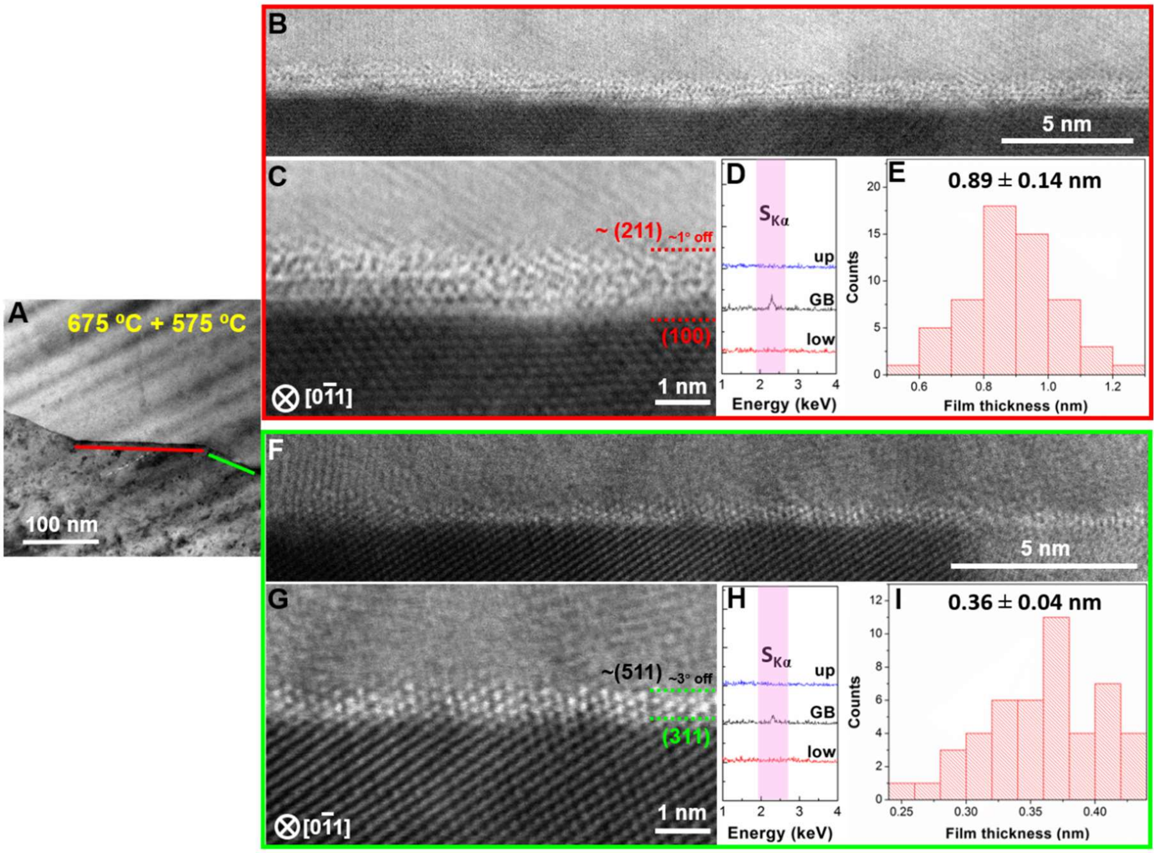

Supplementary Figure 34. (GB #3A and #3B) Type A and Type B facets observed at the same GB #3. This specific GB was randomly selected from a S-doped Ni specimen that was first annealed at 675 °C and subsequently annealed and quenched from 575 °C. **(A)** Low-magnification STEM image showing the GB facets. **(B)** Intermediate and **(C)** high magnification STEM ABF images showing a Type A facet highlighted by the red line in panel (a). The orientation relationship is (100)//~(211)$_{\sim 1^\circ\ \mathrm{off}}$. **(D)** The corresponding EDX spectra obtained from the GB and two adjacent crystals, indicating that this Type A GB facet is S-enriched. **(E)** Histogram showing the statistics of the measured thickness of amorphous-like intergranular films at the Type A facets. The mean thickness is 0.89 ± 0.14 nm. **(F)** Intermediate and **(G)** high magnification STEM ABF images showing a Type B facet highlighted by the green line in panel (a). The orientation relationship is (311)//~(511)$_{\sim 3^\circ\ \mathrm{off}}$. **(H)** The corresponding EDX spectra obtained from the GB and two adjacent crystals, indicating that this Type B GB facet is also S-enriched. **(I)** Histogram showing the statistics of the measured effective interfacial thickness of this Type B complexion.

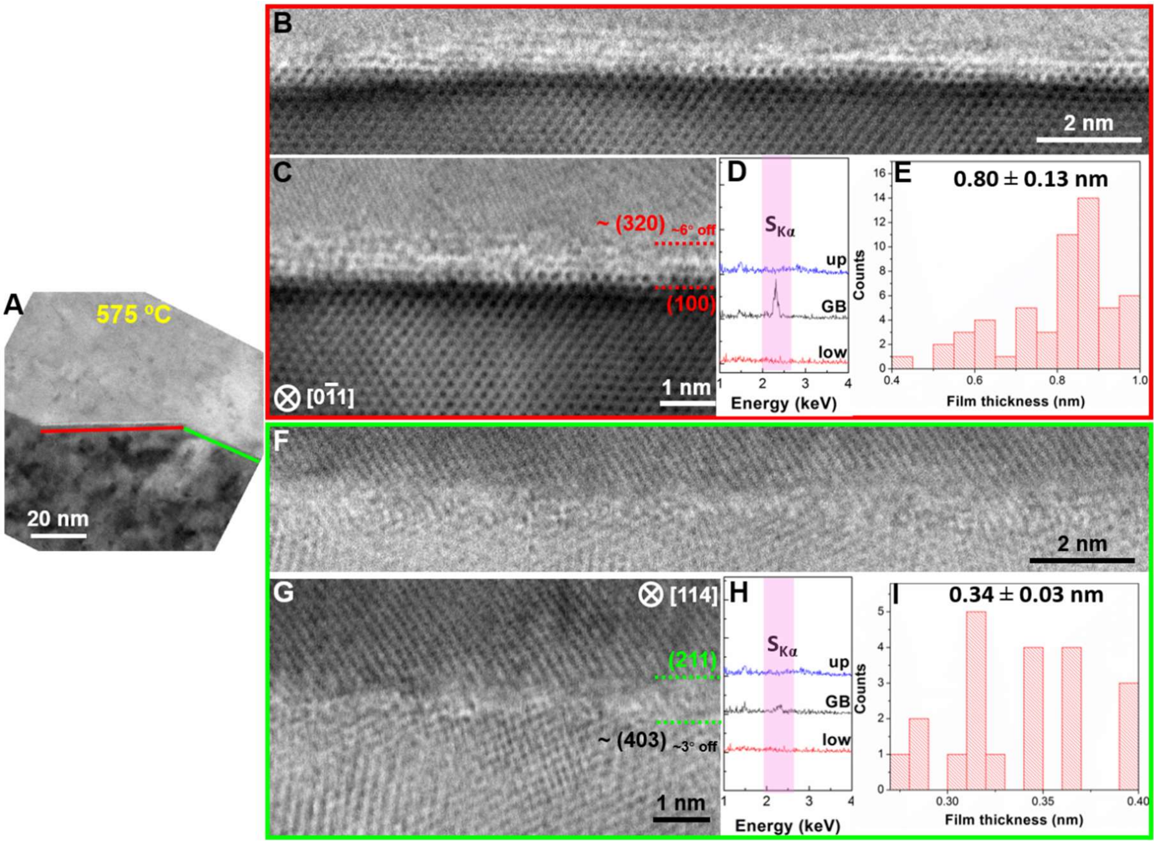

Supplementary Figure 35. (GB #4A and #4B) Type A and Type B facets of the same GB #4. This specific GB was randomly selected from a S-doped Ni specimen annealed and quenched from 575 °C. **(A)** Low-magnification STEM image showing the GB with two different facet types. **(B)** Intermediate-magnification STEM ABF image showing a Type A GB facet highlighted by the red line in panel (a). **(C)** STEM ABF image of the Type A GB facet at a higher magnification; the orientation relationship was determined to be (100)//~$(320)_{\sim 6^\circ\ \mathrm{off}}$. **(D)** The corresponding EDX spectra obtained from the GB and two adjacent crystals, respectively, indicating that this Type A GB facet is S-enriched. **(E)** Histogram showing statistics of the measured thickness of amorphous-like intergranular films at the Type A facets. The mean thickness is 0.80 ± 0.13 nm. **(F)** Intermediate- and **(G)** high-magnification STEM ABF images of a Type B GB facet highlighted by the green line in (a); the orientation relationship is (211)//~$(403)_{\sim 3^\circ\ \mathrm{off}}$. **(H)** The corresponding EDX spectra obtained from the GB and two adjacent crystals, indicating that this Type B GB facet is also S-enrich. **(I)** The histogram of the measured effective interfacial thicknesses of this Type B complexion formed at parallel (different) facets of the same GB.

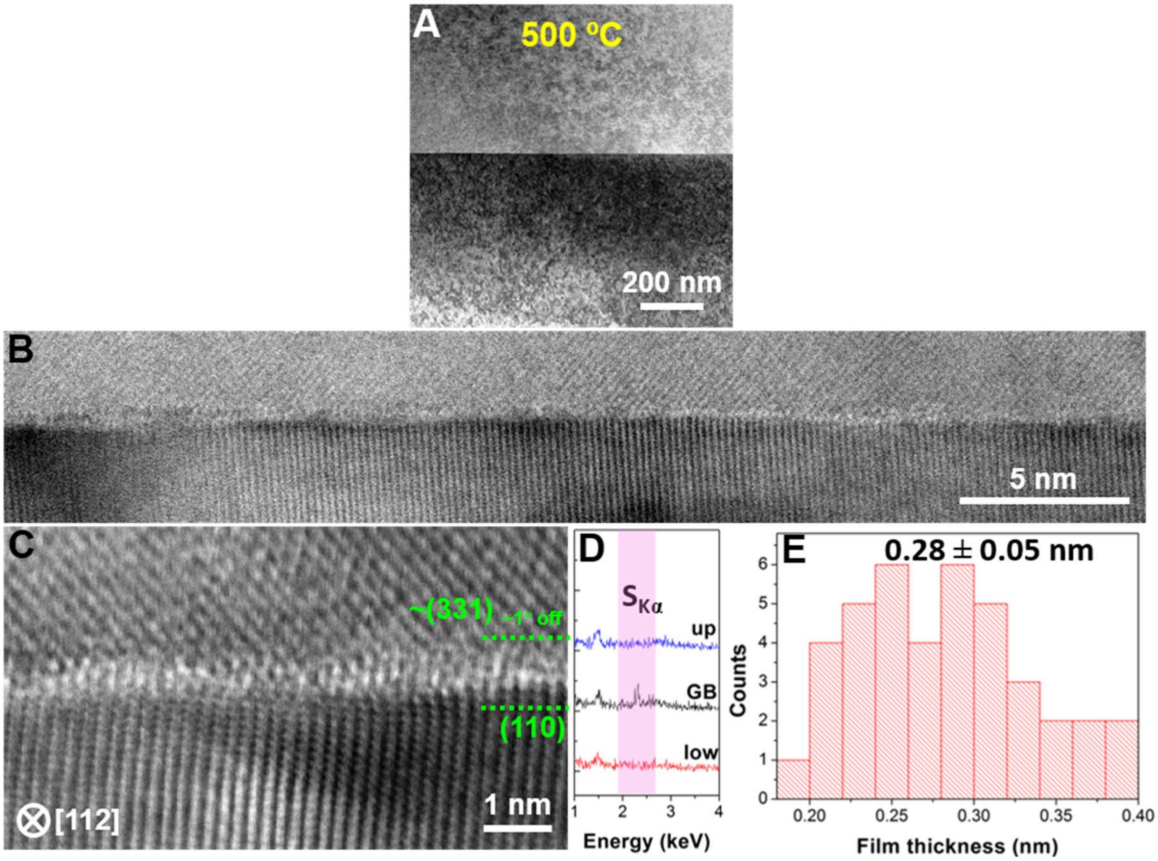

Supplementary Figure 36. (GB #5B) Type B complexion observed on the GB #5. This specific GB was randomly selected from a S-doped Ni specimen annealed and quenched from 500 °C. **(A)** Low-magnification STEM image showing the straight GB. **(B)** Intermediate-magnification STEM ABF image showing Type B GB. **(C)** STEM ABF image of the Type B GB at a higher magnification; the orientation relationship was determined to be (110)//~(331)$_{\sim 1^\circ \text{ off}}$. **(D)** The corresponding EDX spectra obtained from the GB and two adjacent crystals, respectively, which indicate that this the Type B GB is S-enriched. **(E)** Histogram of the measured effective interfacial thicknesses of this Type B complexion.

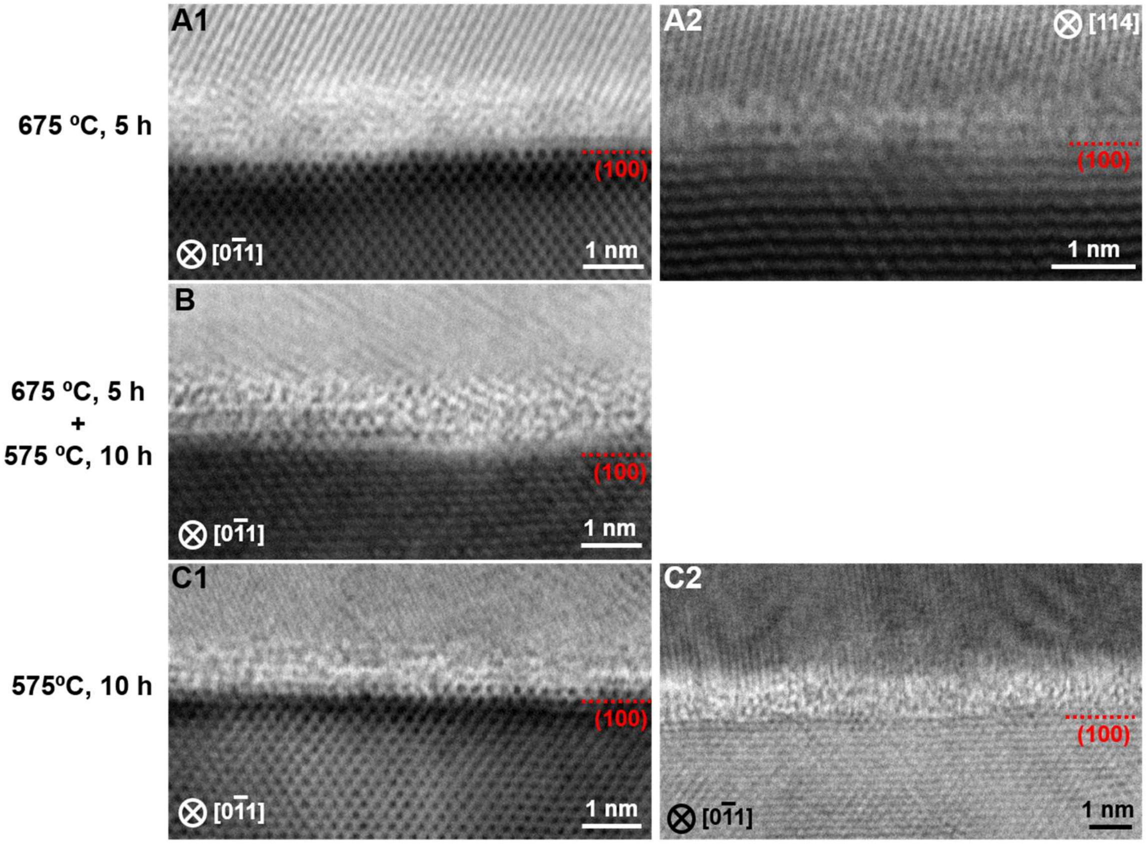

Supplementary Figure 37. Representative STEM ABF images of Type A GB facets observed in five different GBs equilibrated at three different conditions. **(A1)** GB#1A and **(A2)** GB#2A in a sample equilibrated at and quenched from 675 °C. **(B)** GB#3A in a sample that was first annealed at 675 °C and subsequently equilibrated at 575 °C and quenched. **(C1)** and **(C2)** GB#4A in a sample equilibrated at and quenched from 575 °C. In all cases, one of the grain surface exhibits the (100) plane. Furthermore, all 18 independent observations of Type A amorphous-like complexions at 18 independent facets are shown in Supplementary Fig. 29.

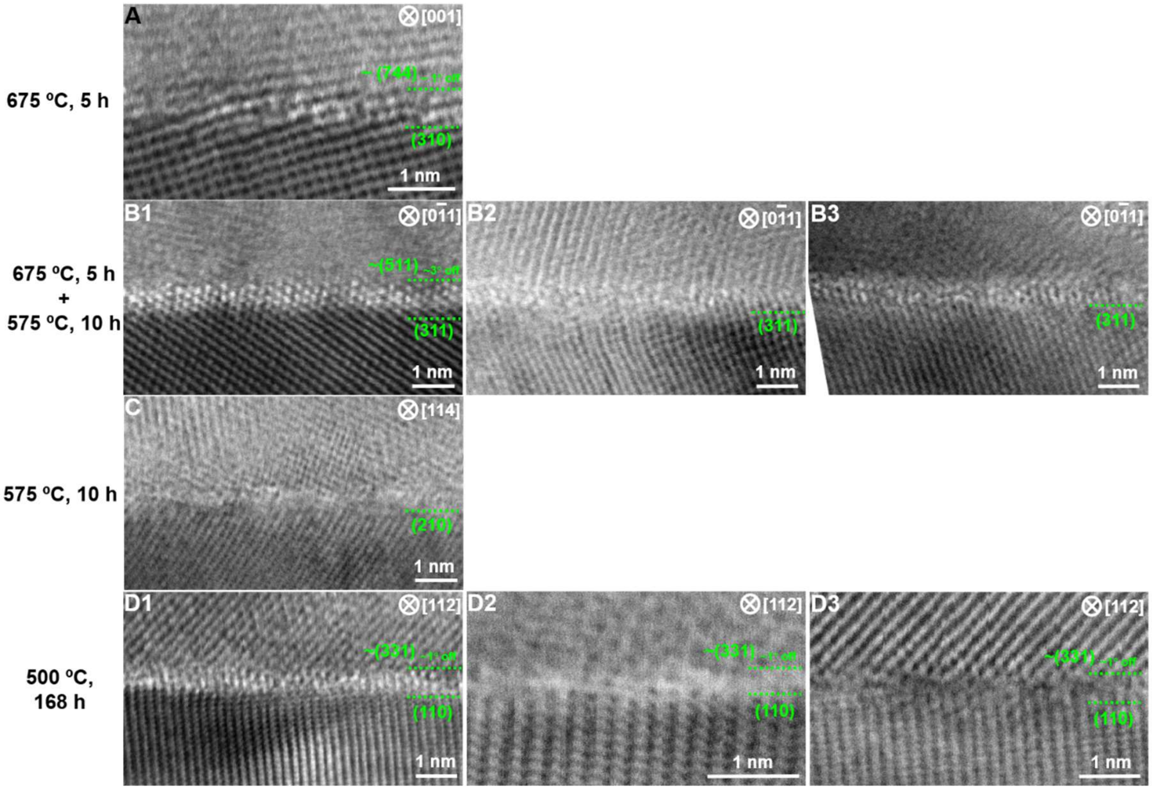

Supplementary Figure 38. Representative STEM ABF images of Type B GB facets observed in four different GBs equilibrated at four different conditions (A) GB#1B in a sample equilibrated at and quenched from 675 °C. **(B1)** to **(B3)** GB#3B in a sample that was first annealed at 675 °C and subsequently equilibrated at 575 °C and quenched. **(C)** GB#4B in a sample equilibrated at and quenched from 575 °C. **(D1)** to **(D3)** GB#5B in a sample equilibrated at and quenched from 500 °C. In all cases, one of grain surfaces have a low Miller index (labeled in the figure) other than (100). Furthermore, all 11 independent observations of Type B bilayer-like complexions at 11 independent facets/locations are shown in Supplementary Fig. 30.

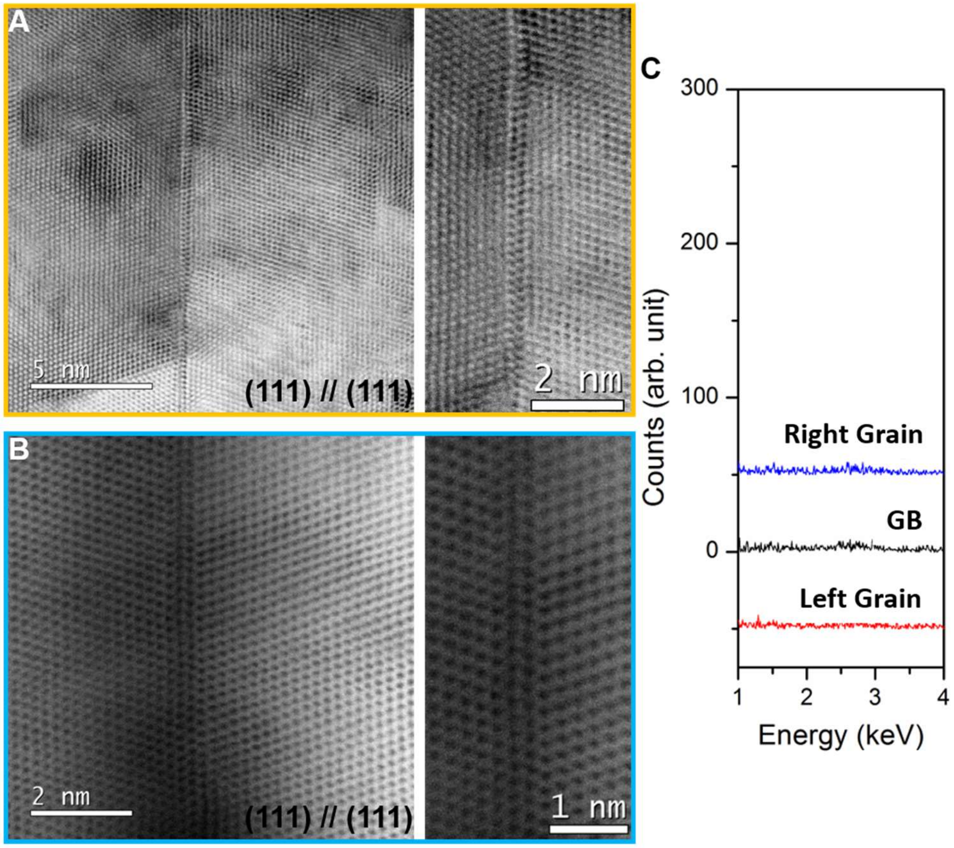

Supplementary Figure 39. Symmetric Σ3 (1110//(111) twin boundaries in S-doped Ni samples without (detectable) S adsorption, which, along with other low-energy GBs (such as low-angle GBs) with little S adsorption, are referred to as Type C or nominally "clean" GBs. (A) A twin boundary from a S-doped Ni sample first annealed at 675 °C and subsequently equilibrated at 575 °C. **(B)** A twin boundary from a S-doped Ni sample equilibrated at 500 °C. **(C)** Typical EDXS spectra obtained from the twin boundary and the adjacent crystals, indicating no detectable S segregation.

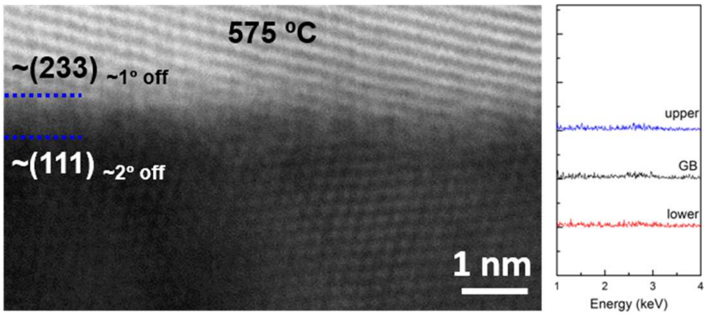

Supplementary Figure 40. Another Type C or "clean" GB with no significant S segregation. This specific GB was observed in the S-doped Ni specimen annealed at 575 °C for 10 hours and subsequently water-quenched. Its orientation relationship was determined to be ~(111)$_{\sim 2^\circ\ \mathrm{off}}$//~(233)$_{\sim 1^\circ\ \mathrm{off}}$; the disorientation angle is about 10° so that it is a low-angle GB with low segregation driving force. The EDX spectra obtained from the GB and the two adjacent crystals are shown at the left side of the figure, and indicate no significant (detectable) S segregation.

Supplementary Note 24: Additional simulation results

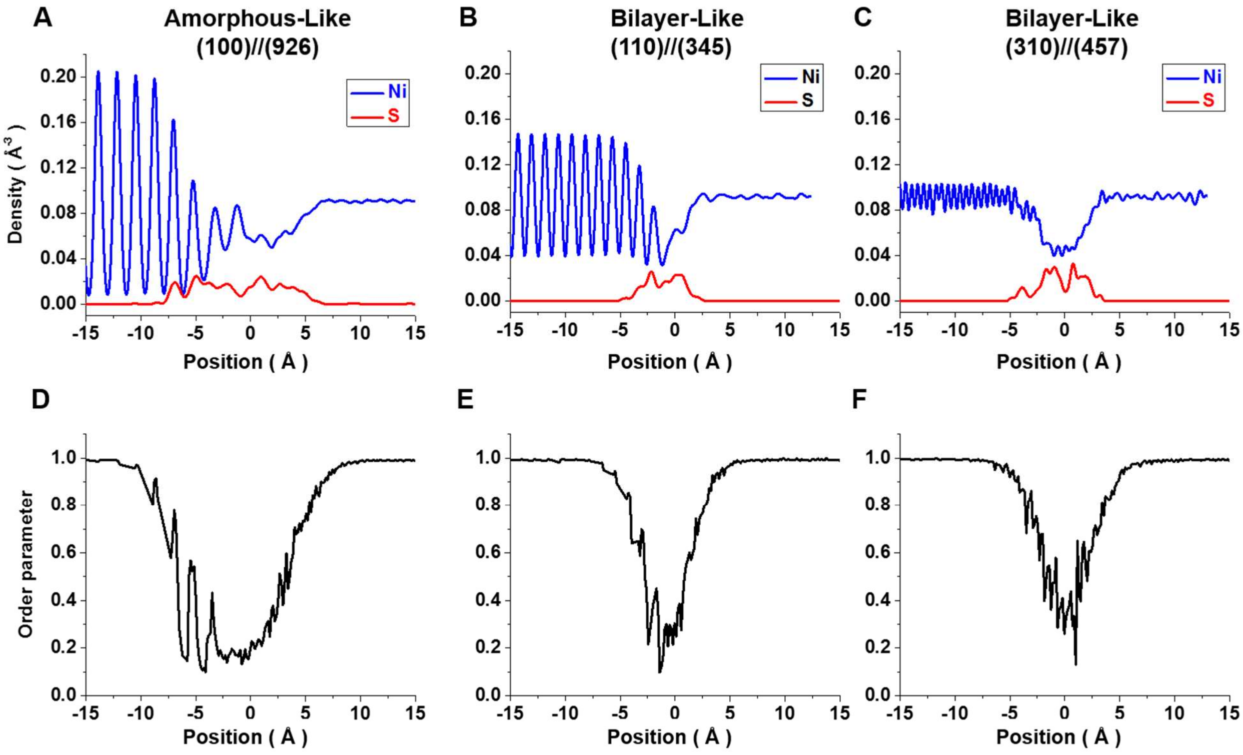

Supplementary Figure 41. Profiles of density and order parameter obtained from atomistic simulations of three GBs. (A), (B), and **(C)** are the profiles of atomic density near the (100)//(926), (110)//(345), and (310)//(457) GB facets, respectively. **(D), (E),** and **(F)** are the profiles of order parameter for these three GBs. On one hand, the disordered region is about 1 nm for the Type A GB complexion, being significantly wider than Type B GB complexions. On the other hand, the profiles of S densities for the Type B GB complexions show two (groups of) major peaks.

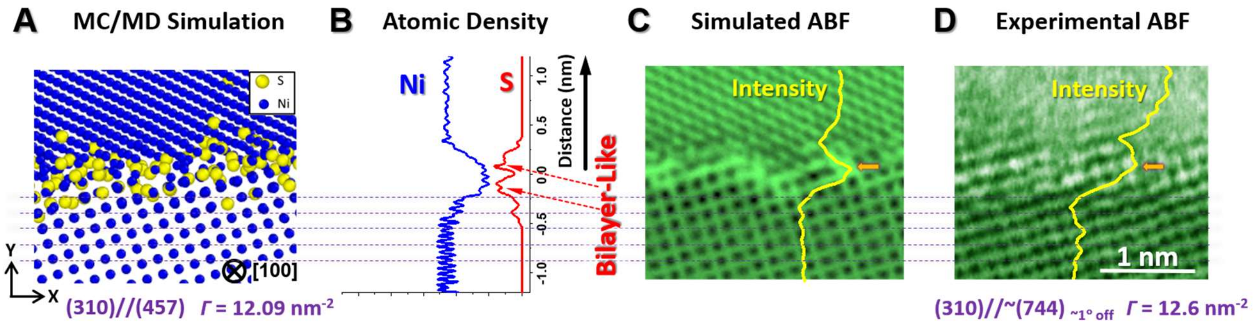

Supplementary Figure 42. Atomic structure of a Type B GB facet (Case II, in addition to the example shown in Fig. 3 of the main article). (A) Hybrid MC/MD simulated (310)//(457) GB facet and associated **(B)** Ni and S density profiles. **(C)** Simulated ABF image using the atomic structure obtained by hybrid MC/MD simulation shown in panel (A). **(D)** Experimental atomic-resolution ABF of the (310)//~(744) GB facet.

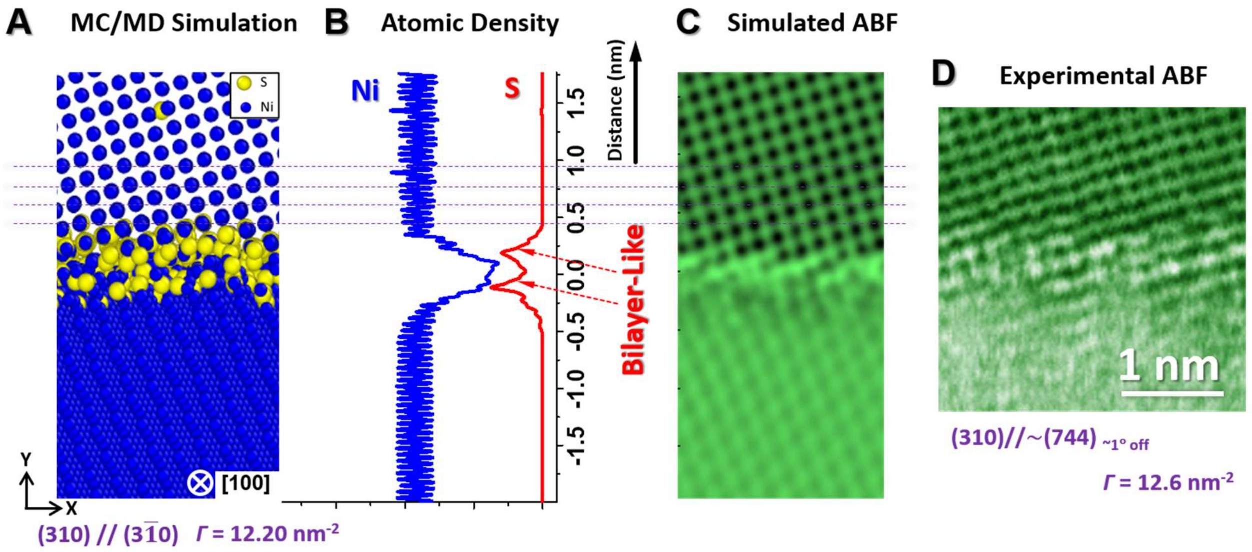

Supplementary Figure 43. Atomic structure of a Type B facet (Case III, in addition to the two examples shown in Fig. 3 of the main article and Supplementary Fig. 42). (A) Hybrid MC/MD the simulated (310)//(3$\bar{1}$0) GB facet and associated **(B)** Ni and S density profiles. **(C)** Simulated ABF image using the atomic structure obtained by hybrid MC/MD simulation shown in panel (A). **(D)** Experimental atomic-resolution ABF of the (310)//~(744) GB facet, which is the same GB as shown in Supplementary Fig. 42D. A comparison of Supplementary Figs. 42 and 43 suggests that the formation of the GB complexion is dictated by the orientation of the lower-index grain terminating surface plane, instead of misorientation, because the Σ11 (310)//(3$\bar{1}$0) GB facet shown here exhibits an almost identical adsorption amount and similar atomic density profile as the Σ3 (310)//(457) GB facet shown in Supplementary Fig. 42D. In both cases, the images and simulated GB excess adsorption amounts of S matches well with the experimental observation of the (310)//~(744) GB facet.

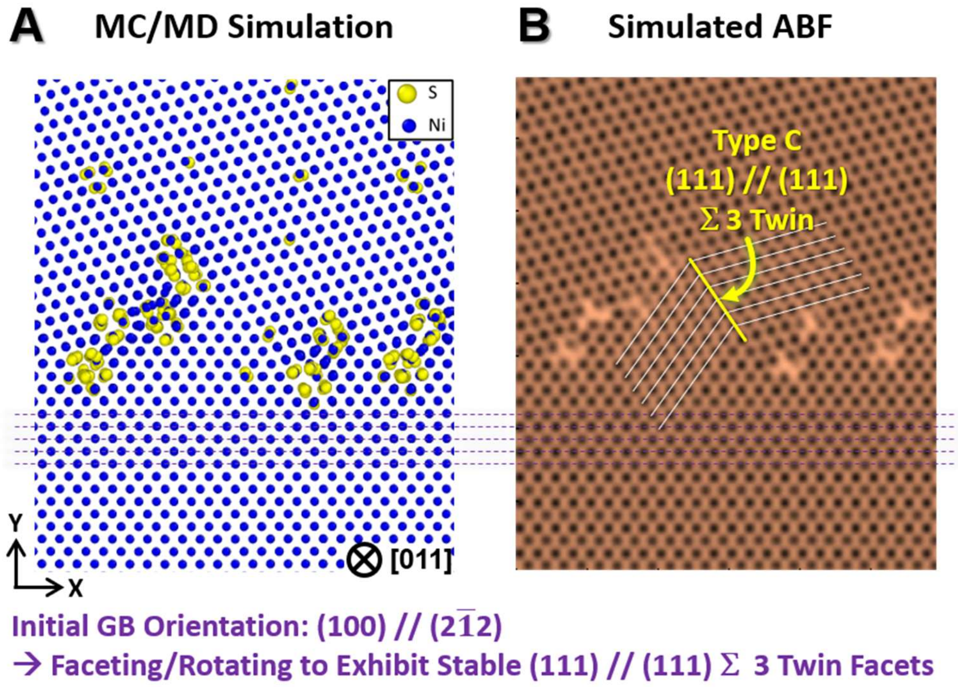

Supplementary Figure 44. Atomic structure of a GB exhibiting nanoscale faceting and Type C symmetric Σ3 (111)//(111) twin facets (with virtually no S adsorption on the twin facets and clusters of S atoms in between). (A) Hybrid MC/MD simulated GB and associated **(B)** simulated ABF image using the atomic structure obtained by hybrid MC/MD simulation shown in (A). The average GB orientation was initially set to be Σ3 (100)//(2$\bar{1}$2), but it faceted during the MC/MD simulation, producing a Type C "clean" facet of the symmetric (111)//(111) twin sections with S enriched clusters in between (where we believe that the simulation time was too short and cell was too small to grow longer, well-developed facets). The formation of a Type C "clean" symmetric (111)//(111) Σ3 twin boundary agrees with our experimental observations shown in Supplementary Figs. 31 and 39.

Supplementary Note 25: SEM micrographs of fractured faceted grain surfaces

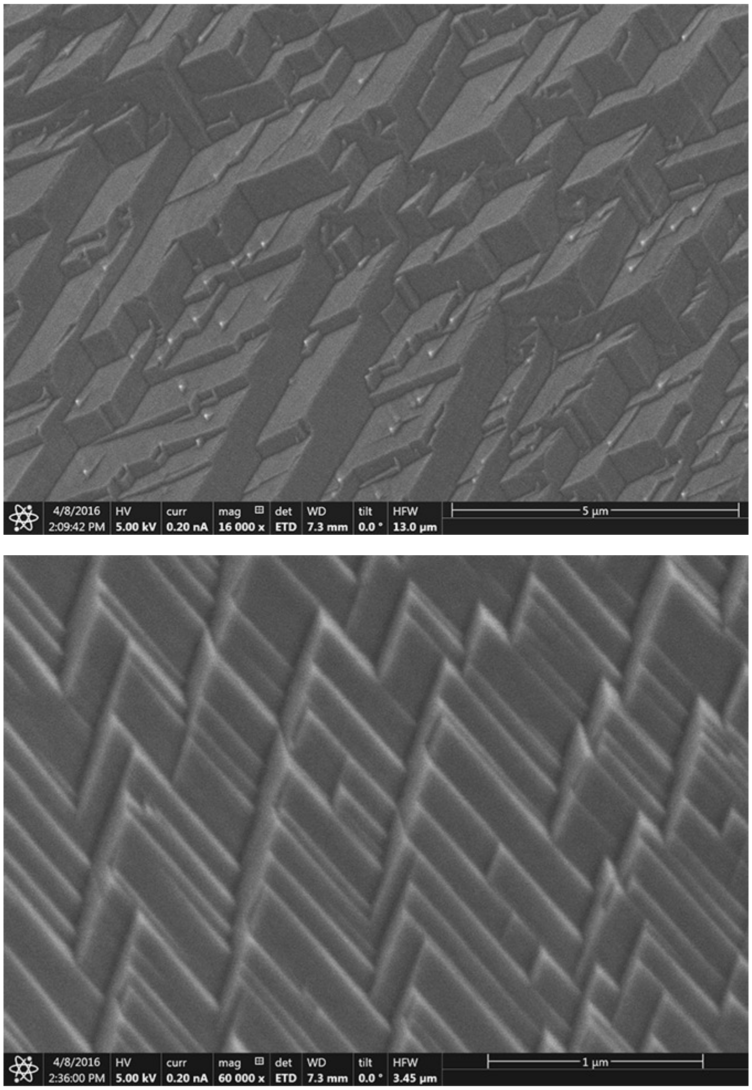

Supplementary Figure 45. Selected SEM micrographs of fractured grain surfaces, showing GB faceting. This specimen was annealed at 675 °C × 5 h and quenched.

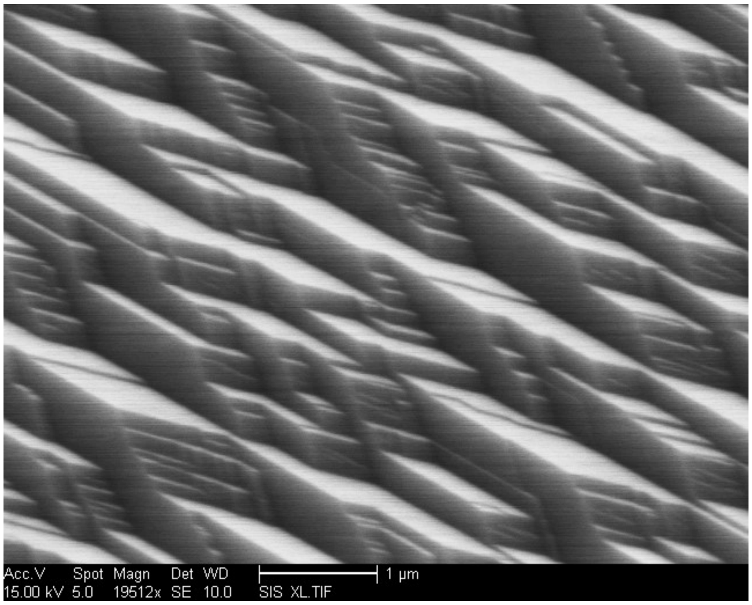

Supplementary Figure 46. Selected SEM micrographs of fractured grain surfaces, showing GB faceting. This specimen was annealed at 675 °C × 5 h + 575 °C × 10 h and quenched.

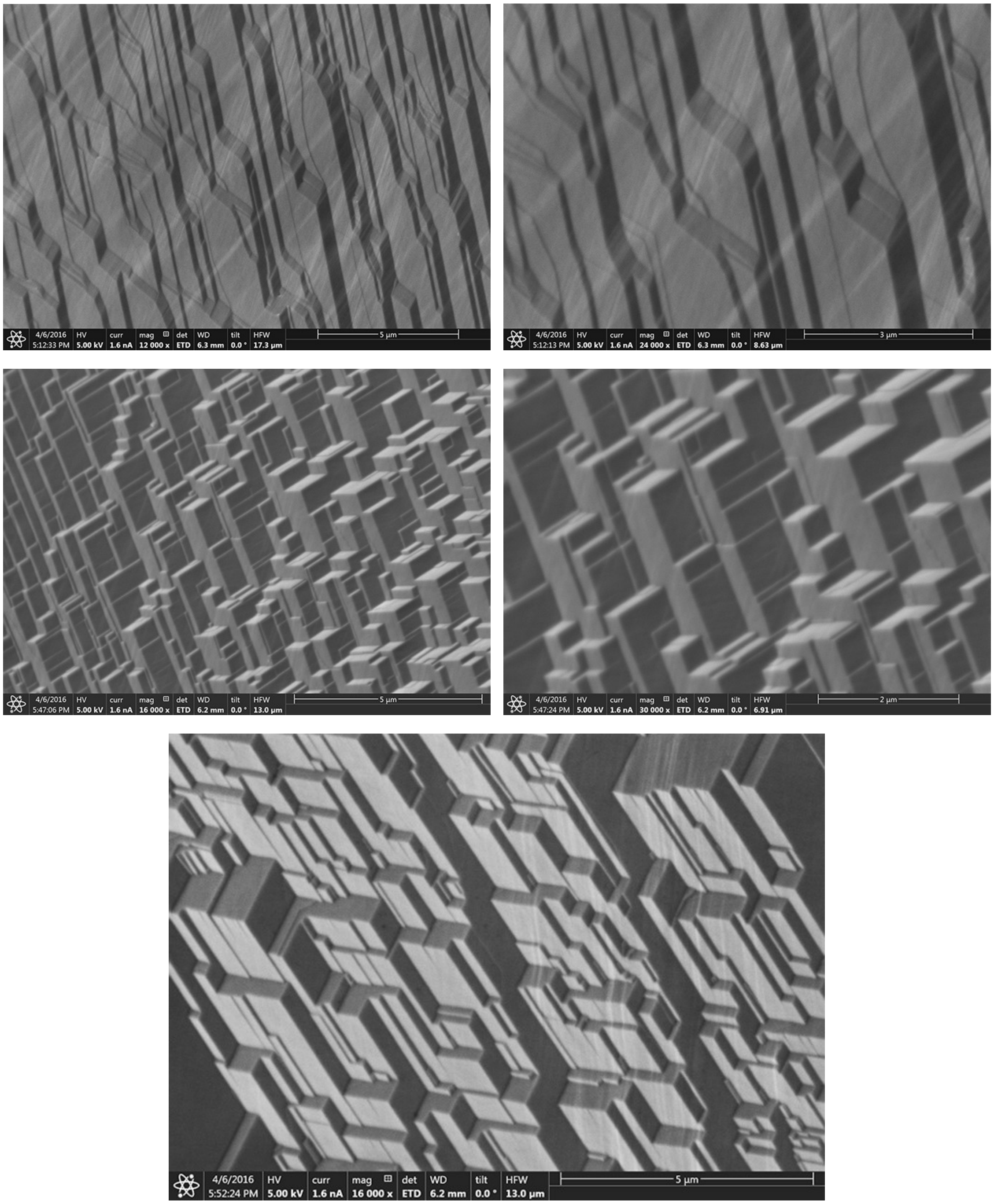

Supplementary Figure 47. Selected SEM micrographs of fractured grain surfaces, showing GB faceting. This specimen was annealed at 575 °C × 10 h and quenched.

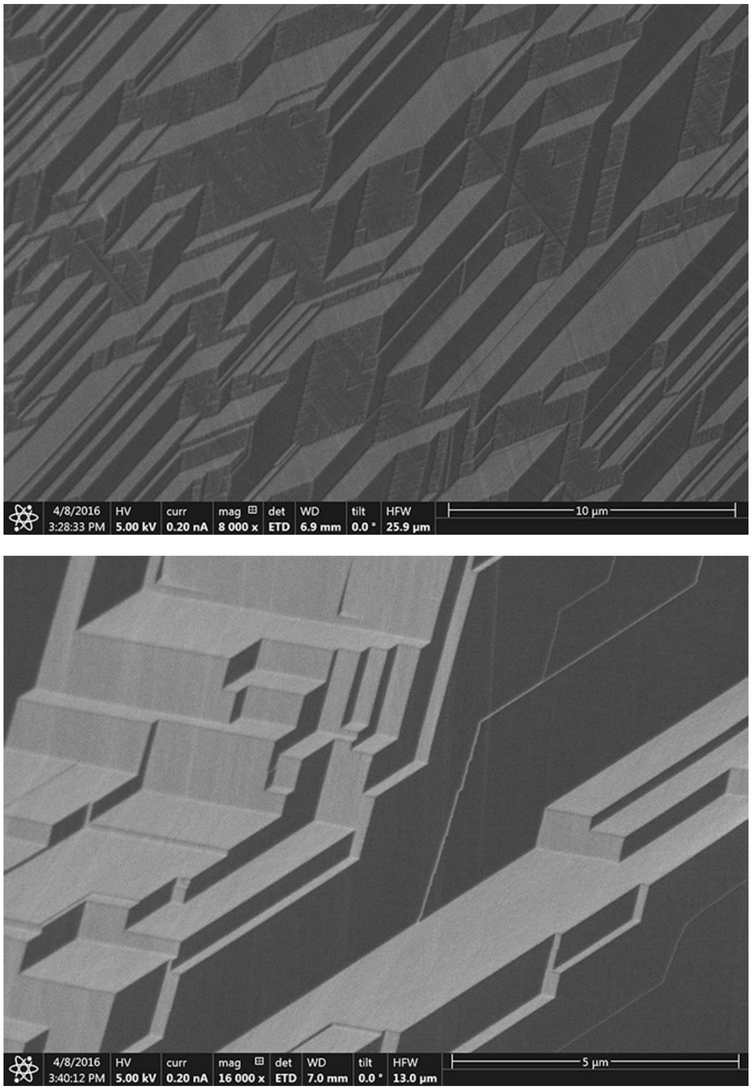

Supplementary Figure 48. Selected SEM micrographs of fractured grain surfaces, showing GB faceting. This specimen was annealed at 500 °C × 168 h and quenched.

Supplementary References

1 Baram, M., Chatain, D. & Kaplan, W. D. Nanometer-thick equilibrium films: the interface between thermodynamics and atomistics. *Science* **332**, 206-209 (2011).

2 Kaplan, W. D., Chatain, D., Wynblatt, P. & Carter, W. C. A review of wetting versus adsorption, complexions, and related phenomena: the rosetta stone of wetting *Journal of Materials Science* **48**, 5681-5717 (2013).

3 Cantwell, P. R. *et al.* Grain boundary complexions. *Acta Mater.* **62**, 1-48 (2014).

4 Findlay, S. *et al.* Dynamics of annular bright field imaging in scanning transmission electron microscopy. *Ultramicroscopy* **110**, 903-923 (2010).

5 Abe, E. *et al.* Direct Imaging of Hydrogen Atoms in a Crystal by Annular Bright-field STEM. *Microsc. Microanal.* **17**, 1278 (2011).

6 Ishizuka, K. & Uyeda, N. A new theoretical and practical approach to the multislice method. *Acta Crystallographica Section A: Crystal Physics, Diffraction, Theoretical and General Crystallography* **33**, 740-749 (1977).

7 Ikeda, J. A. S., Chiang, Y. M., Garratt-Reed, A. J. & Sande, J. B. V. Space Charge Segregation at Grain Boundaries in Titanium Dioxide: II, Model Experiments. *J. Am. Ceram. Soc.* **76**, 2447-2459 (2010).

8 Cliff, G. & Lorimer, G. W. The quantitative analysis of thin specimens. *J. Microscopy* **103**, 203-207 (1975).

9 Chen, H. P. *et al.* Embrittlement of metal by solute segregation-induced amorphization. *Phys. Rev. Lett.* **104**, 155502 (2010).

10 Olmsted, D. L., Foiles, S. M. & Holm, E. A. Survey of computed grain boundary properties in face-centered cubic metals: I. Grain boundary energy. *Acta Mater.* **57**, 3694-3703 (2009).

11 Larere, A., Guttmann, M., Dumoulin, P. & Roques-Carmes, C. Auger electron spectroscopy study of the kinetics of intergranular and surface segregations in nickel during annealing. *Acta Metall.* **30**, 685-693 (1982).

12 Sharma, R. C. & Chang, Y. A. Thermodynamics and phase relationships of transition metal-sulfur systems: IV. thermodynamic properties of the Ni-S liquid phase and the calculation of the Ni-S phase diagram. *Metallurgical and Materials Transactions B* **11**, 139-146 (1980).

13 Kirkland, A. & Saxton, W. Cation segregation in Nb16W18O94 using high angle annular dark field scanning transmission electron microscopy and image processing. *J. Microscopy* **206**, 1-6 (2002).

14 Rice, J. R. & Wang, J. S. Embrittlement of interfaces by solute segregation. *Materials Science & Engineering A* **107**, 23-40 (1989).

15 Heuer, J. K., Okamoto, P. R., Lam, N. Q. & Stubbins, J. F. Disorder-induced melting in nickel: implication to intergranular sulfur embrittlement. *J. Nucl. Mater.* **301**, 129-141 (2002).

16 Heuer, J. K., Okamoto, P. R., Lam, N. Q. & Stubbins, J. F. Relationship between segregation-induced intergranular fracture and melting in the nickel-sulfur system. *Appl. Phys. Lett.* **76**, 3403-3405 (2000).

17 Yamaguchi, M. First-principles study on the grain boundary embrittlement of metals by solute segregation: Part I. iron (Fe)-solute (B, C, P, and S) systems. *Metallurgical and Materials Transactions A* **42**, 319-329 (2011).

18 Yamaguchi, M. & Kameda, J. Multiscale thermodynamic analysis on fracture toughness loss induced by solute segregation in steel. *Philos. Mag.* **94**, 2131-2149 (2014).

19 Zhao, J. & Adams, B. Definition of an asymmetric domain for intercrystalline misorientation in cubic materials in the space of Euler angles. *Acta Crystallogr. Sect. A: Found. Crystallogr.* **44**, 326-336 (1988).

20 Zhu, G.-H., Mao, W.-M. & Yu, Y.-N. Calculation of misorientation distribution between recrystallized grains and deformed matrix. *Scripta Mater.* **42**, 37-41 (1999).

21 Luo, J. & Harmer, M. P. The role of a bilayer interfacial phase on liquid metal embrittlement. *Science* **333**, 1730-1733 (2011).

22 Zhao, B. Abnormal grain growth with {1 0 0} planar interface in the electrodeposited nickel. *Mater. Res. Innovations* **19**, S251-S254 (2015).

23 Cheng, L. & Hibbard, G. Abnormal grain growth via the migration of planar growth interfaces. *Materials Science and Engineering: A* **492**, 128-133 (2008).

24 Tsurekawa, S., Fukino, T. & Matsuzaki, T. In-situ SEM/EBSD observation of abnormal grain growth in electrodeposited nanocrystalline nickel. *International Journal of Materials Research* **100**, 800-805 (2009).

25 Dillon, S. J., Tang, M., Carter, W. C. & Harmer, M. P. Complexion: A new concept for kinetic engineering in materials science. *Acta Mater.* **55**, 6208-6218 (2007).

26 Harmer, M. P. Interfacial Kinetic Engineering: How Far Have We Come Since Kingery's Inaugural Sosman Address? *J. Am. Ceram. Soc.* **93**, 301-317 (2010).
